# Experimental and theoretical studies on the synthesis of 1,4,5-trisubstituted pyrrolidine-2,3-diones

**DOI:** 10.3762/bjoc.18.118

**Published:** 2022-08-31

**Authors:** Nguyen Tran Nguyen, Vo Viet Dai, Nguyen Ngoc Tri, Luc Van Meervelt, Nguyen Tien Trung, Wim Dehaen

**Affiliations:** 1 Department of Chemistry, University of Science and Education, the University of Da Nang, Ton Duc Thang 459, 550000 Da Nang, Viet Nam; 2 Laboratory of Computational Chemistry and Modelling, Faculty of Natural Sciences, Quy Nhon University, An Duong Vuong 170, 820000 Quy Nhon, Viet Nam; 3 Biomolecular Architecture, Department of Chemistry, KU Leuven, Celestijnenlaan 200F, B-3001 Leuven, Belgium; 4 Molecular Design and Synthesis, Department of Chemistry, KU Leuven, Celestijnenlaan 200F, B-3001 Leuven, Belgium

**Keywords:** 4-acetyl-3-hydroxy-3-pyrroline-2-ones, 1,5-dihydro-2*H*-pyrrol-2-ones, pyrrolidine-2,3-dione, 2-pyrrolidinone derivative, 3-pyrroline-2-one

## Abstract

Substituted 4-acetyl-3-hydroxy-3-pyrroline-2-ones have been prepared via three-component reactions and the tautomerism of these 3-pyrroline-2-ones is due to the slight difference of energy, and the significantly large rate constant of transformation between two tautomers. 1,4,5-Trisubstituted pyrrolidine-2,3-dione derivatives were prepared from the above mentioned 2-pyrrolidinone derivatives and aliphatic amines, which exist in enamine form and are stabilized by an intramolecular hydrogen bond. A possible reaction mechanism between 3-pyrroline-2-one and aliphatic amine (CH_3_NH_2_) was proposed based on computational results and the main product is formed favorably following the PES via the lowest Δ*G*^#^ pathway in both the gas-phase and an ethanol solvent model. DFT calculations showed that kinetic selectivity is more significant than thermodynamic selectivity for forming main products.

## Introduction

**Figure 1:**
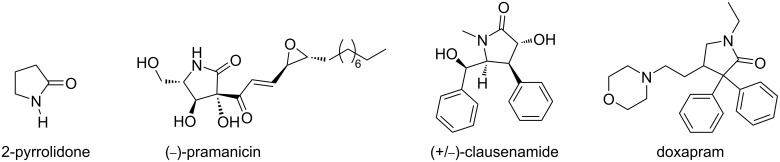
Structure of naturally occurring and synthetic 2-pyrrolidone derivatives.

2-Pyrrolidone, also known as γ-lactam, is a five-membered heterocyclic ring containing four carbon and one nitrogen atoms [1]. This γ-lactam structure is an important scaffold which can be found in many pharmaceutical active natural products and synthetic medicinal compounds [2]. Pramanicin, for example, is a 2-pyrrolidone-containing natural product isolated from a lactose-containg liquid fermentation of a sterile fungus growing in grass which showed interesting antimicrobial and antibacterial activities [2–4]. (−)-Clausenamide was extracted and isolated from *Clausena lansium* which exhibited biological activities to enhance learning and memory capacities in amnesia animal models [5–6]. Doxapram, a non-natural compound, has been used to form doxapram hydrochloride which helps to increase the respiratory rate [7] (Figure 1).

**Figure 2:**
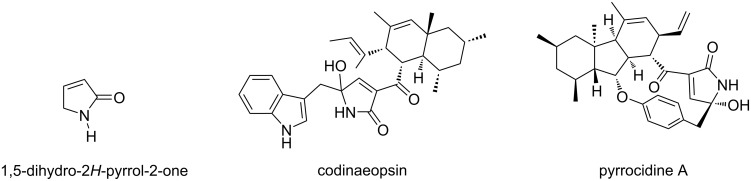
Structure of natural compounds containing a 1,5-dihydro-2*H*-pyrrol-2-one subunit.

Among the 2-pyrrolidinone derivatives, 1,5-dihydro-2*H*-pyrrol-2-ones, also named as 3-pyrrolin-2-ones, are important builiding blocks which can be further modified in organic synthesis and medicinal chemistry [8–10]. In addition, these 2-oxopyrroles are structural subunits of various bioactive natural compounds and synthetic drugs. For example, codinaeopsin has been isolated from a fungal extract of a tree called *Vochysia guatemalensis* which shows antimalarial activity [11]. Pyrrocidine A was isolated from the fungal endophyte *Acremonium zeae* and its chemical structure was elucidated in 2002 (Figure 2). This natural 3-pyrrolin-2-one derivative exhibits potent antivity against Gram-positive bacteria [12]. Moreover, 1,5-dihydro-2*H*-pyrrol-2-ones and especially, 3-hydroxy-1,5-dihydro-2*H*-pyrrol-2-ones are also important substructures in a variety of non-natural compounds and their pharmacological effects against bacteria [13–16], inflammation [17–18], viruses [19], radical [20], and cancer [21–26] have been proven.

It is undoubtedly true that heterocyclic compounds containing a 3-pyrroline-2-one skeleton could be promising drug candidates. Therefore, these nitrogen-containing heterocycles have attracted attention and they have been investigated most intensively via multicomponent reactions (MCRs). This kind of reaction has been proven to be an efficient synthetic pathway to obtain structurally complicated and biologically active products containing substantial portions of all starting materials [27]. Recently, polysubstituted 3-hydroxy-1,5-dihydro-2*H*-pyrrol-2-ones have been synthesized via eco-friendly multicomponent reactions of aromatic aldehydes, amines, and dialkyl acetylenedicarboxylate or sodium diethyl oxalacetate [28–34]. The resulting products have the 4-position locked by the alkoxycarbonyl (–COOR) group and these 2-pyrrolidinone derivatives can be functionalized with amines as nucleophiles via the 3-position [10,35–38].

Acyl or aroyl groups can be introduced into the 4-position of 3-hydroxy-1,5-dihydro-2*H*-pyrrol-2-ones via three-component reactions of esters of acylpyruvic acid or of aroylpyruvic acid, respectively, with aromatic aldehydes, and amines. In addition, the resulting 2-pyrrolidinone derivatives also showed antibacterial and antifungal activities [39–41]. The presence of an acyl group at the 4-position enables these heterocycles to be functionalized via nucleophilic addition reactions between the carbonyl group and nucleophiles like hydroxylamine and semicarbazide [42].

Herein, we report the synthesis of 4-acetyl-3-hydroxy-3-pyrroline-2-ones via multicomponent reactions of ethyl 2,4-dioxovalerate with aromatic aldehydes, and aniline in glacial acetic acid. These 2-pyrrolidinone derivatives were then reacted with aliphatic amines in ethanol to obtain a library of 1,4,5-trisubstituted pyrrolidine-2,3-dione enamine derivatives. As compared to glacial acetic acid [42], ethanol has showed to be the best solvent for the synthesis of these pyrrolidine-2,3-diones and a dramatic increase in the yield of the desired products was also observed. In addition, understanding of the reaction mechanism at the molecular level brings an efficient evaluation and good orientation in experimental work [43]. Density functional theory (DFT) calculations were used to explore the mechanistic aspects of the reaction between 4-acetyl-3-hydroxy-1,5-diphenyl-3-pyrrolin-2-one and methylamine (CH_3_NH_2_) to achieve 4-(1-methylamino)ethylene-1,5-diphenylpyrrolidine-2,3-dione in the present work. To the best of our knowledge, it is the first time the reaction mechanism between 3-pyrrolin-2-one derivative and methylamine was explained in detail via computational studies.

## Results and Discussion

**Scheme 1:**
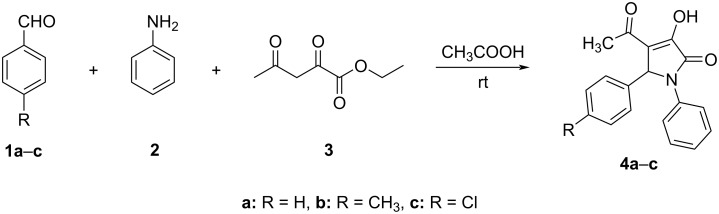
Synthesis of substituted 4-acetyl-3-hydroxy-3-pyrroline-2-ones **4** via three-component reaction.

A model reaction of benzaldehyde (**1a**), aniline (**2**), and ethyl 2,4-dioxovalerate (**3**) in glacial acetic acid was used for optimization (Scheme 1). Reacting equimolar amounts of **1a**, **2**, and **3**, 0.5 M in acetic acid as solvent at room temperature [39], resulted in the formation of 1,5-diphenyl-4-acetyl-3-hydroxy-3-pyrrolin-2-one (**4a**) in a yield of 70%. However, an increase in the yield of product **4a** to 77% could be observed with the ratio of 1:1:1.5 of reactants **1a**, **2**, and **3**, respectively (Table 1).

**Table 1:** Starting materials **1a**:**2**:**3** ratio optimization for the synthesis of product **4a**.

Entry	Ratio **1a**:**2**:**3** (equiv)	Concentration **1a**:**2**:**3** (mmol/mL)	Yield (%)

1	1:1:1	0.5:0.5:0.5	70%
2	1:1:1.5	0.5:0.5:0.75	77%
3	1.5:1:1	0.75:0.5:0.5	80%
4	1:1.5:1	0.5:0.75:0.5	67%

The product **4a** was obtained in 80% yield when the concentration of the aromatic aldehyde **1a** in solvent increased to 0.75 M while remaining the amounts of **2** and **3** unchanged. However, increasing the concentration of aniline (**2**) in acetic acid to 0.75 M while keeping the amounts of **1** and **3** constant at 0.5 M resulted in a slight decrease of the yield (67%) of 2-pyrrolidinone derivative **4a** (Table 1). Therefore, the ratio 1.5:1:1 of reactants, the aromatic aldehyde, aniline, and ethyl 2,4-dioxovalerate, respectively, in acetic acid as solvent was used to synthesize other substituted 4-acetyl-3-hydroxy-3-pyrroline-2-ones.

**Scheme 2:**
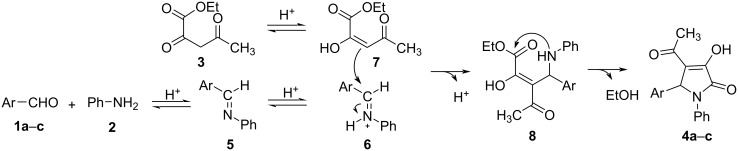
Proposed mechanistic path for the synthesis of substituted 4-acetyl-3-hydroxy-3-pyrroline-2-ones.

It may be surmised that the first step in the three-component reaction to synthesize substituted 4-acetyl-3-hydroxy-3-pyrroline-2-ones **4a**–**c** occurs via the acid-catalyzed condensation of the aromatic aldehyde **1a**–**c** and aniline (**2**) to produce imine intermediate **5** which is then protonated to the iminium species **6**. In addition, ethyl 2,4-dioxovalerate (**3**) containing an activated methylene group is in fast equilibrium with enol derivative **7** in acidic media, the latter playing the role of nucleophilic reagent which attacks to iminium salt **6** to give intermediate **8**. Subsequently, intramolecular nucleophilic attack of the amino moiety to the carboxylate group in the intermediate **8** leads to the formation of the target products **4a**–**c** (Scheme 2).

The reaction of aromatic aldehyde **1a** and aniline (**2**) in acidic environment is reversible [44] and therefore, according to Le Chatelier’s principle [45], the increase in the concentration of **1a** or **2** will shift the equilibrium to the side of the iminium species **6**. As the concentration of **2** was increased to 0.75 M as compared to 0.5 M of aldehyde **1a**, the equilibrium would favor the side of iminium salt **6**. However, the excess amount of the nucleophilic reactant **2** reacted with intermediate **6** and as a consequence, the yield of the product **4a** decreased to 67% (Table 1, Scheme 2).

**Scheme 3:**
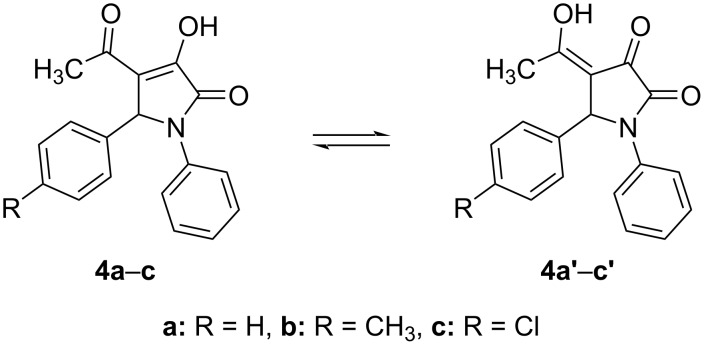
Tautomerism of compounds **4a**–**c** in DMSO.

The ^1^H NMR spectra of compounds **4a**–**c** in DMSO-*d*_6_ showed peaks of aromatic protons in the chemical shift region of 6.97–7.59 ppm, a singlet at 6.00–6.06 ppm for the proton at the 5-position of the 3-pyrrroline-2-one moiety. In addition, along with peaks of carbons at the 2,5-positions of the N-containing heterocyclic ring and signals of the aromatic carbons, the ^13^C NMR spectra of these compounds in DMSO-*d*_6_ also exhibited broad peaks at around 30 ppm and 191 ppm. The NMR spectra of the 2-pyrrolidinone derivative **4c** were also recorded in CDCl_3_ and its ^1^H NMR spectrum is similar to the one observed in DMSO-*d*_6_. However, the ^13^C NMR spectrum of **4c** in CDCl_3_ showed sharp peaks at 29.21 ppm and 194.61 ppm representing the two carbon atoms of the acetyl group attached to the 4-postion of the heterocyclic five-membered ring. Therefore, the broadening of peaks observed in the ^13^C NMR spectra is due to the tautomerism of compounds **4a**–**c** in DMSO [46], a hydrogen-bond accepting solvent (Scheme 3).

To clarify further this statement, DFT calculations were performed at the B3LYP/6-311++G(2d,2p)//B3LYP/6-31+G(d,p) level of theory as given in Table 2 and Table 3. The theoretical results show that structure **4a** is more stable than **4a’** by 1.3 kcal·mol^−1^ in the gas phase or by 0.4 kcal·mol^−1^ in the ethanol solvent model. The transformation of **4a** into **4a’** occurs with a small potential barrier of 0.5 kcal·mol^−1^ (in the gas phase) or 1.0 kcal·mol^−1^ (in ethanol). This process goes through **TS1** by H displacement at the O–H bond of the O–H···O intramolecular hydrogen bond (distance ca. 1.7–1.8 Å) (cf. Figures 4–6). The isomerization of **4a/4a’** is extremely fast due to the high rate constant of about 10^12^ s^−1^ at 298 K, calculated by the transitional state theory method and quantum tunneling effect [47]. It is thus impossible to distinguish **4a** and **4a’** (Scheme 3). Consequently, isomers **4a** and **4a’** coexist during the synthesis of **10ab** from the reaction of **4a**/**4a’** and CH_3_NH_2_, being consistent with our experimental work (cf. Table 5).

**Table 2:** The thermodynamic parameters of possible reaction pathways in the gas phase (kcal·mol^−1^) at the B3LYP/6-311++G(2d,2p)// B3LYP/6-31+G(d,p) level of theory.

Entry	Stage	Δ*E*	Δ*G*	Δ*E*^#^	Δ*G*^#^	I-Freq (cm^−1^)	Note

1	**4a’**→ **4a**	−1.3	−1.0	0.5	0.6	−1156.69	**TS1**
2	**4a’** → **IS5**	10.1	22.9	21.9	34.6	−212.87	**TS2**
3	**4a’** → **IS3**	9.0	21.3	37.5	50.0	−1565.52	**TS3**
4	**4a’** → **IS2**	14.5	27.0	44.8	57.2	−1612.98	**TS4**
5	**4a** → **IS1**	−3.6	5.3				
6	**4a** → **IS3**	10.3	22.3	22.9	34.7	−186.59	**TS5**
7	**4a** → **IS4**	16.7	29.2	46.1	58.3	−1630.70	**TS6**
8	**IS2** → **IS4**	0.9	1.2	1.0	1.3	−1099.78	**TS7**
9	**IS1** → **IS5**	15.0	15.9	41.7	45.2	−1521.85	**TS8**
10	**IS3** → **IS5**	1.1	1.6	58.3	59.0	−335.98	**TS9**
11	**IS3** → **IS6**	−1.6	−1.1	6.7	7.4	−139.56	**TS10**
12	**IS4** → **IS7**	−1.1	−1.3	3.9	4.7	−115.85	**TS11**
13	**IS5** → **IS8**	1.0	0.7	1.7	2.5	−64.84	**TS12**
14	**IS2** → **10ab-v4**	−6.3	15.1	26.7	26.9	−216.31	**TS13**
15	**IS5** → **IS9**	−8.6	−19.4	41.6	31.8	−1083.98	**TS14**
16	**IS6** → **IS10**	−9.3	−20.7	28.8	28.4	−359.26	**TS15**
17	**IS7** → **IS11**	−1.0	−11.8	26.5	26.9	−276.33	**TS16**
18	**IS4** → **10ab-v3**	−1.7	−13.2	33.9	31.8	−314.94	**TS17**
19	**IS8** → **10ab**	−15.7	−26.6	11.1	10.7	−902.87	**TS18**
20	**IS3** → **10ab-v2**	−20.1	−30.7	17.3	17.3	−1078.98	**TS19**
21	**IS9** → **10ab**	−6.1	−6.5				
22	**IS10** → **10ab-v2**	−9.2	−8.9				
23	**IS11** → **10ab-v3**	0.4	−0.1	7.2	7.1	−1366.77	**TS20**

**Table 3:** The thermodynamic parameters of selected pathways (1), (2), (3), and (4) in ethanol (kcal·mol^−1^) at the B3LYP/6-311++G(2d,2p)// B3LYP/6-31+G(d,p) level of theory.

Entry	Stage	Δ*E*	Δ*G*	Δ*E*^#^	Δ*G*^#^	I-Freq (cm^−1^)	Note

1	**4a’** → **4a**	−0.4	−0.01	1.0	1.4	−1169.88	**TS1**
2	**4a’** → **IS5**	10.1	23.0	14.3	27.1	−240.38	**TS2**
3	**4a’** → **IS3**	14.5	26.8	38.4	35.7	−1670.26	**TS3**
4	**4a** → **IS1**	−7.3	2.2				
5	**4a** → **IS3**	14.9	26.8	19.4	31.4	−197.98	**TS5**
6	**IS1** → **IS5**	17.8	20.8	27.0	45.4	−1648.76	**TS8**
7	**IS3** → **IS6**	−2.9	−2.3	6.0	6.6	−135.85	**TS10**
8	**IS5** → **IS8**	1.3	1.3	2.1	2.1	−63.84	**TS12**
9	**IS5** → **IS9**	−11.3	−21.8	34.7	34.4	−272.88	**TS14**
10	**IS6** → **IS10**	−11.7	−22.7	24.5	23.8	−252.49	**TS15**
11	**IS8** → **10ab**	−22.1	−32.8	6.1	5.6	−748.04	**TS18**
12	**IS3** → **10ab-v2**	−24.7	−35.2	13.4	13.4	−831.36	**TS19**
13	**IS9** → **10ab**	−9.5	−9.7				
14	**IS10** → **10ab-v2**	−10.1	−10.2				

Three substituted 3-hydroxy-3-pyrroline-2-ones **4a**–**c** were then reacted with aliphatic amines and the structures of the obtained compounds were elucidated by NMR and mass spectrometry. The reaction between 1,5-diphenyl-4-acetyl-3-hydroxy-3-pyrrolin-2-one (**4a**) and 4-methoxybenzylamine (**9a**) was chosen to optimize the reaction conditions, such as the reactant ratio and solvent. Heating 2-pyrrolidinone derivative **4a** (1 equiv) and aliphatic amine **9a** (2.5 equiv) in glacial acetic acid [42] resulted in the formation of product **10aa** with a yield of only 36%. It is clear that compound **9a** could be protonated in the acidic environment, decreasing its nucleophilicity and therefore, product **10aa** was obtained in low yield. The yield of **10aa** could be increased to 62% when substrate **4a** was reacted with the amine **9a** in DMF at 95 °C (Table 4), meaning that the reaction does not require an acidic medium.

**Table 4:** Optimization of the reaction conditions for the synthesis of compound **10aa**.

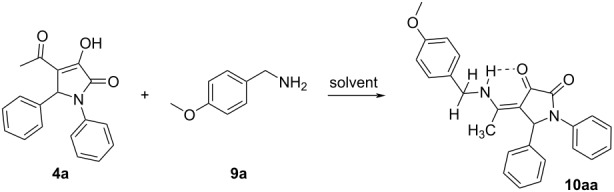						

Entry	Ratio **4a**:**9a** (equiv)	Solvent	Volume (mL)	*T* (°C)	*t* (hour)	Yield (%)

1	1:2.5	DMF	1	95	6	62
2	1:2.5	AcOH	1	95	9.5	36
3	1:1	ethanol	1	80	5	69
4	1:2.5	ethanol	1	80	3	72
5	1:2.5	ethanol	1	80	5	73
6	1:4	ethanol	1	80	5	82
7	1:4	ethanol	0.5	80	5	86
8	1:4	ethanol	0.34	80	5	89

Ethanol was also used as green solvent to test the above model reaction. As compared to CH_3_COOH, heating compounds **4a** (1 equiv, 0.17 mmol) and **9a** (2.5 equiv, 0.43 mmol) in 1 mL of ethanol at 80 °C led to a dramatic increase in the yield of product **10aa**, 72%. In addition, 2-pyrrolidinone derivative **10aa** could be obtained in 82% yield when the amount of the amine **9a** was increased to 4 equiv. Unsurprisingly, the yield of the desired product **10aa** reached 89% when the solvent volume was lowered to 0.34 mL (Table 4). The optimized procedure was then applied to the synthesis of other 1,4,5-trisubstituted pyrrolidine-2,3-dione enamine derivatives with high yields (Table 5).

**Table 5:** Synthesis of a library of 1,4,5-trisubstituted pyrrolidine-2,3-diones.

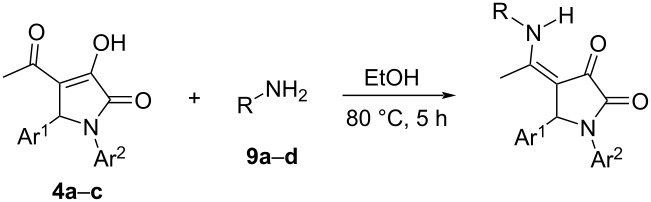					

Entry	Ar^1^	Ar^2^	R	Product	Yield (%)

1	C_6_H_5_	C_6_H_5_	4-MeOC_6_H_4_CH_2_	**10aa**	89
2	C_6_H_5_	C_6_H_5_	CH_3_	**10ab**	88
3	C_6_H_5_	C_6_H_5_	C_6_H_5_CH_2_	**10ac**	90
4	C_6_H_5_	C_6_H_5_	CH_3_CH_2_OCOCH_2_	**10ad**	82
5	4-MeC_6_H_4_	C_6_H_5_	4-MeOC_6_H_4_CH_2_	**10ba**	76
6	4-MeC_6_H_4_	C_6_H_5_	CH_3_	**10bb**	84
7	4-MeC_6_H_4_	C_6_H_5_	C_6_H_5_CH_2_	**10bc**	81
8	4-MeC_6_H_4_	C_6_H_5_	CH_3_CH_2_OCOCH_2_	**10bd**	87
9	4-ClC_6_H_4_	C_6_H_5_	4-MeOC_6_H_4_CH_2_	**10ca**	81
10	4-ClC_6_H_4_	C_6_H_5_	CH_3_	**10cb**	77
11	4-ClC_6_H_4_	C_6_H_5_	C_6_H_5_CH_2_	**10cc**	79
12	4-ClC_6_H_4_	C_6_H_5_	CH_3_CH_2_OCOCH_2_	**10cd**	80

The ^1^H NMR spectrum of product **10aa** showed a broad peak corresponding to the proton of the secondary amino group (NH) at a chemical shift of 11.74 ppm. The intramolecular hydrogen bond led to the appearance of a resonance signal of the amino group at high chemical shift. Also, the coupling between the amino proton and methylene protons was observed. In addition, the methylene protons are diastereotopic and they show geminal coupling. As a consequence, the two methylene protons were observed as two doublets of doublets at 4.40 ppm and 4.49 ppm, respectively. Moreover, the HMBC 2D NMR spectrum of compound **10aa** also exhibited the correlation between the amino proton and the carbon of the methyl group separated by three σ-bonds. Thus, the reaction between 2-pyrrolidinone derivatives **4a–c** and nucleophilic amines **9a–d** is confirmed to occur at the carbonyl group of the acetyl moiety to achieve 1,4,5-trisubstituted pyrrolidine-2,3-dione derivatives present in the enamine form and stabilized by intramolecular hydrogen bonds (Table 5).

**Figure 3:**
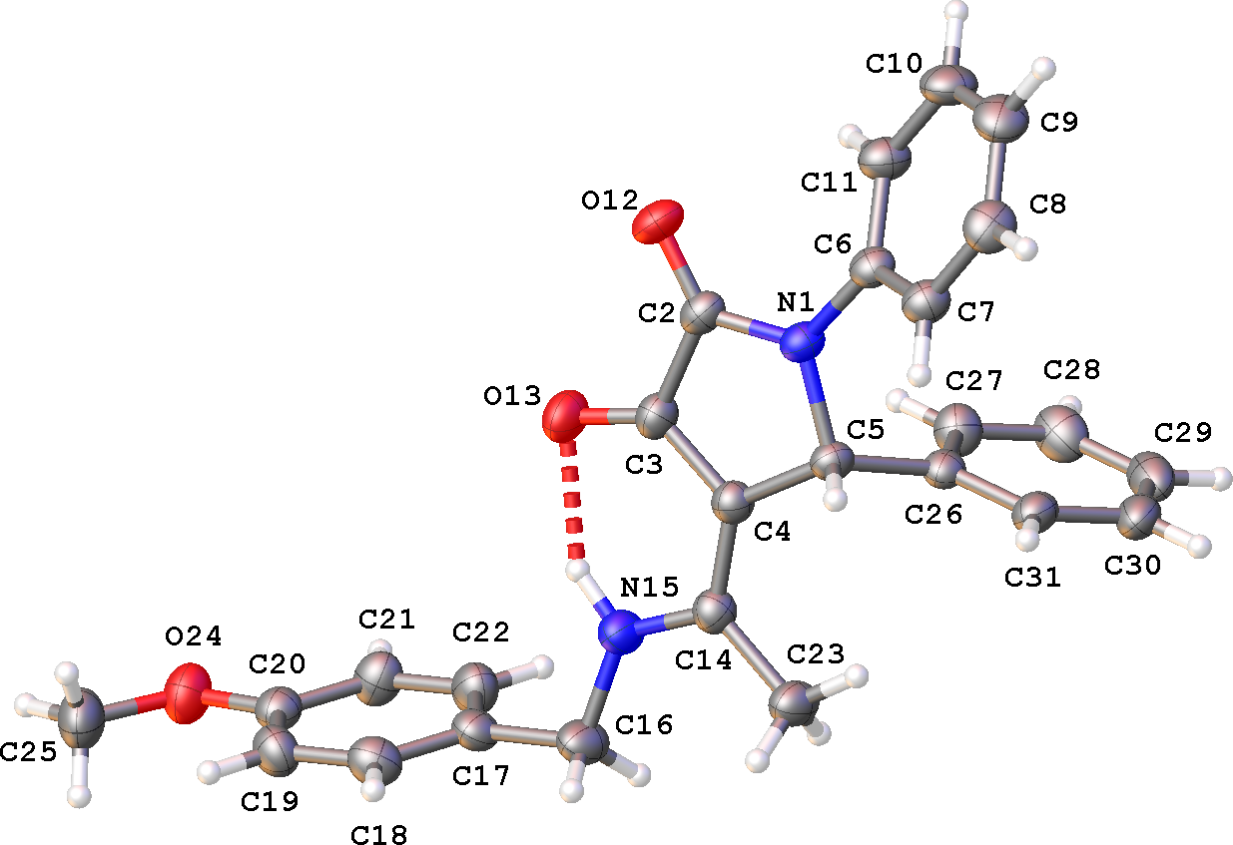
View of the molecular structure of compound **10aa** with atom labeling. Displacement ellipsoids are drawn at the 30% probability level. The intramolecular hydrogen bond N15–H15···O13 is shown as dashed line.

Single-crystal X-ray diffraction confirmed the structure of **10aa** (Figure 3) as well as the presence of the intramolecular N15–H15···O13 hydrogen bond [N15–H15: 0.92(3) Å, H15···O13: 1.90(3) Å, N15···O13: 2.699(3) Å, N15–H15···O13: 145(3)˚]. The pyrrolidine-2,3-dione ring is planar (rms deviation: 0.003 Å) and makes an angle of 40.39(14)˚ with the phenyl ring C6–C11 and of 82.23(15)˚ with the phenyl ring C26–C31. The dihedral angle between these phenyl rings is 74.87(14)˚. The crystal packing of product **10aa** (Figure S1 in Supporting Information File 1) is characterized by C7–H7···O12^i^ hydrogen bonds resulting in chain formation along the *a* direction [C7–H7: 0.93 Å, H7···O12: 2.42 Å, C7···O12: 3.325(3) Å, C7–H7···O12: 164˚; symmetry code: (i) 1 + *x*, *y*, *z*]. Neighboring chains connect by C–H···π interactions between C29–H29 and phenyl ring C6–C11 [H29···Cg^ii^: 2.77 Å, Cg is the centroid of ring C6–C11, symmetry code: (ii) 1 − *x*, 2 − *y*, 1 − *z*].

**Figure 4:**
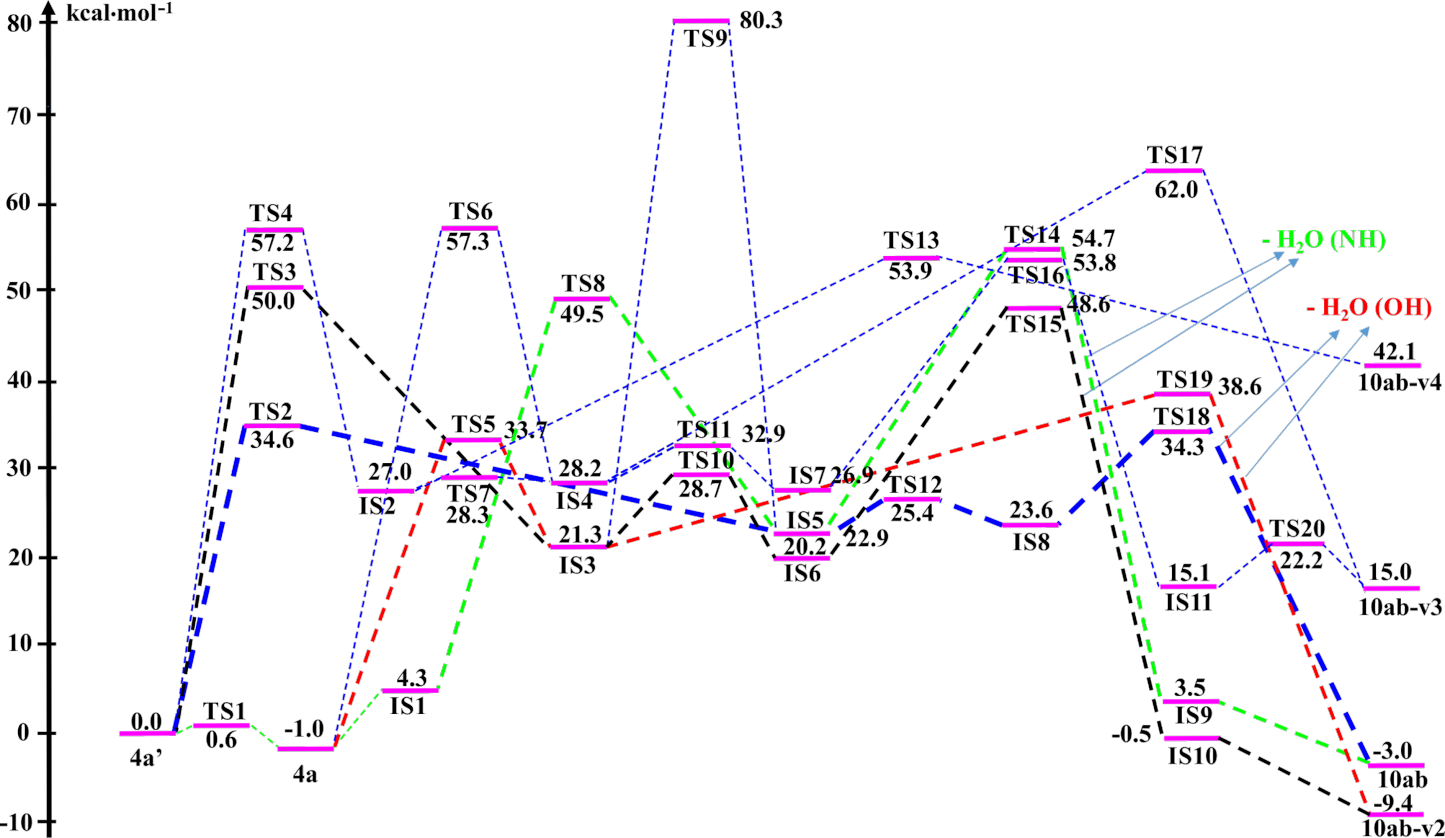
The PES of reaction for the synthesis of 1,4,5-trisubstituted pyrrolidine-2,3-dione **10ab** enamine derivative in the gas phase.

As a model example, the reaction between substrate **4a** and methylamine (**9b**) was used to elucidate the mechanism to obtain product **10ab** via DFT calculations at the B3LYP/6-311++G(2d,2p)// B3LYP/6-31+G(d,p) level of theory. To consider the reaction pathways of **4a**, **4a’** to form product **10ab** synthesized from experimental work, we optimized all possible geometries of **4a**, **4a’**, and **10ab**. The structures and their corresponding energy values are listed in Table S2 and shown in Figures S2, S3, and S4 in Supporting Information File 1. Accordingly, the most stable shapes of **4a**, **4a’**, and **10ab** are considered in the potential energy surface (PES) and displayed in Figure 4 and Figure 6. Fortunately, these structures have similar stereochemical configurations. Further, the structures of **IS5** and **10ab-v2** are also considered with all possible isomers and are listed in Figures S5 and S6, and Table S2 (Supporting Information File 1). It is noticeable that the **IS5** and **10ab-v2** are formed via the reaction between **4a/4a’** and **9b** (CH_3_NH_2_) without any changes in stereochemistry. The reactants, intermediates, transition states, and products were optimized and are illustrated in Figure S7 in Supporting Information File 1. The relative free energy (Δ*G*, Δ*G*^#^) values for all species are presented in Figure 4, the reaction potential energy surface. This figure is a detailed reaction mechanism to form product **10ab** and isomers from **4a/4a’** in the gas phase. The Δ*G*, Δ*G*^#^ values for each stage in the PES at the gas phase are collected in Table 2.

The **4a** and **4a’** tautomers react with CH_3_NH_2_ to yield products, as detailed in Figure 4. All three C (C−O) sites in **4a**, **4a’** are possible for interaction with CH_3_NH_2_ to obtain 2-pyrrolidinone derivative **10ab** and its isomers (**10ab-v2**, **10ab-v3**, **10ab-v4**) (Figure S7 in Supporting Information File 1). It is clear that the attack of CH_3_NH_2_ on the C (C−O) sites to form intermediates (ISs) takes place on the opposite side of the benzene ring (C−C_6_H_5_). This is due to the steric hindrance of the C_6_H_5_-groups attached to the 1- and 5-position of the heterocyclic ring. The 3D-optimized structures of substances on the PES are illustrated in Figures S2, S3, S4, S5, S6 and S7 in Supporting Information File 1.

**Scheme 4:**
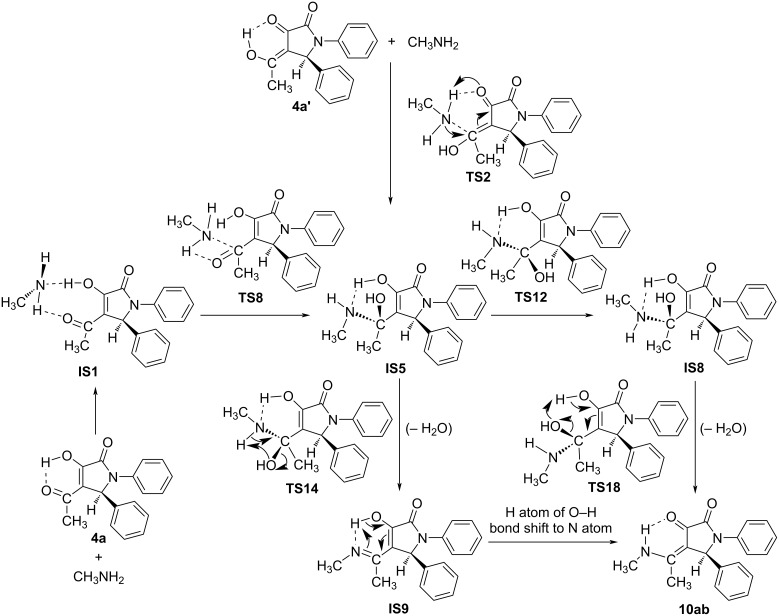
Reaction pathways from **4a**, **4a’** to **10ab** via **IS5** in the gase phase.

For the reaction pathway starting from **4a**, firstly, it interacts with CH_3_NH_2_ to form a stable complex (**IS1**), which is then transfered to the **IS5** intermediate via the **TS8** transition state with a high potential barrier of 49.5 kcal·mol^−1^. From the **IS5** intermediate, there are two possible ways to yield product **10ab** by removing one H_2_O molecule from either two O–H groups or O–H and N–H groups. Before a H_2_O molecule is released from two O–H groups, **IS5** firstly converts to its isomer **IS8** through the **TS12** transition state with a relatively small potential barrier (ca. 2.6 kcal·mol^−1^). **IS8** then removes H_2_O from two close O–H bonds to form the product **10ab** via **TS18** with an activation energy of about 10.7 kcal·mol^−1^. On the other hand, if the removal of one H_2_O molecule occurs from the O–H and N–H groups, **IS5** transforms into **IS9** through **TS14** with a high barrier of 31.8 kcal·mol^−1^. **IS9** is then converted to product **10ab** based on the shifting of H atom from the O–H bond to N atom (Scheme 4). This process is thermodynamically favorable by a Gibbs free energy (Δ*G*) of −6.5 kcal·mol^−1^.

**Scheme 5:**
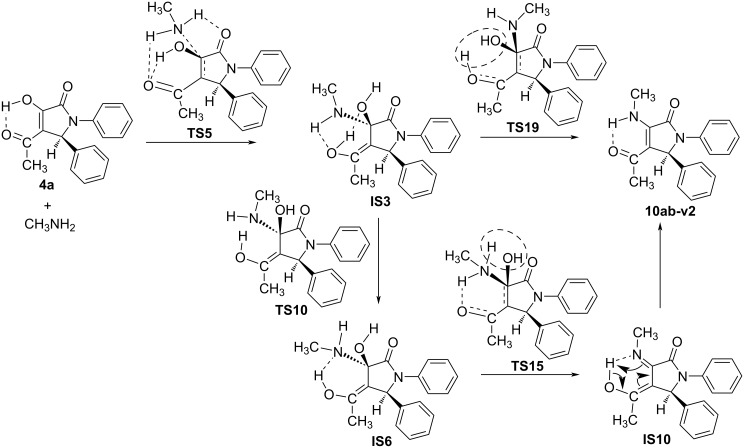
Reaction pathways from **4a** to **10ab-v2** via **IS3** in the gase phase.

In addition, a second way from **4a**, through the **TS5** transition state, to form the **IS3** intermediate requires an activation energy of ca. 33.7 kcal·mol^−1^. **IS3** could release one H_2_O molecule from two O–H groups to obtain **10ab-v2** through the **TS19** transition state with a barrier of 17.3 kcal·mol^−1^. Alternatively, **IS3** will firstly transfer to its isomer **IS6**, and then **IS10** by removing one H_2_O molecule from O–H and N–H groups and finally produce **10ab-v2** via isomerization with H shift (Scheme 5). The first two stages occur through the **TS10**, **TS15** transition states with *E*_a_ values ca. 7.4 and 28.4 kcal·mol^−1^, respectively. The last stage is a thermodynamically favorable H-shift process with Δ*G* of −8.9 kcal·mol^−1^ which is similar to the transformation from **IS9** to **10ab**. In addition, we found a more difficult way to yield **IS5** from **IS3** via **TS9** with a high barrier of 59.0 kcal·mol^−1^.

The most unlikely direction from **4a** is to create an **IS4** intermediate via the **TS6** transition state with a high potential barrier (ca. 58.3 kcal·mol^−1^). From **IS4**, one H_2_O molecule is cleaved from two O–H bonds to produce **10ab-v3**. This process passes through **TS17** with an *E*_a_ of 33.8 kcal·mol^−1^. By performing an isomerization of **IS4** it is easy to obtain **IS7**, then removal of one H_2_O molecule from O–H and N–H groups occurs to yield **IS11**. The isomerization of **IS11** via the shift of a H atom in the O–H group to form an N–H bond leads to **10ab-v3** (Scheme S1, Supporting Information File 1). These processes are carried out through the **TS11**, **TS16**, and **TS20** transition states with Δ*G*^#^ values of 4.7, 26.9, and 7.1 kcal·mol^−1^, respectively.

For the reaction pathway starting from **4a’**, the first steps lead to intermediates **IS5** and **IS3** via transitions states **TS2** and **TS3** with Δ*G*^#^ values of 34.6 and 50.0 kcal·mol^−1^, respectively. Then, as compared to the pathways of **4a**, **IS5** and **IS3** follow the same directions to obtain **10ab**, **10ab-v2**. Particularly, the reaction occurs through **TS4** to give **IS2** with a significant potential barrier of about 57.2 kcal·mol^−1^. The **10ab-v4** product is formed later by the removal of a H_2_O molecule from **IS2** via **TS13** with Δ*G*^#^ about 26.9 kcal·mol^−1^. Furthermore, **IS2** could convert efficiently to the **IS4** isomer by a rapid isomerization with a low barrier of 1.3 kcal·mol^−1^.

In summary, in the gas phase, the reaction pathway starting from **4a** through **IS3** and finally yielding **10ab-v2** is a more favorable one in both thermodynamic (Δ*G*) and kinetic (Δ*G*^#^) aspects (red and black lines) in comparision with going through **IS5** to achieve the **10ab** product. For the reaction originating from **4a’**, passing through **IS5** and finally forming product **10ab** is the most preferred pathway, and even more favorable than both directions from **4a** to yield **10ab-v2**. Hence, the reaction routes to obtain **10ab-v2** are thermodynamically favorable, while yielding **10ab** is under kinetic control.

**Figure 5:**
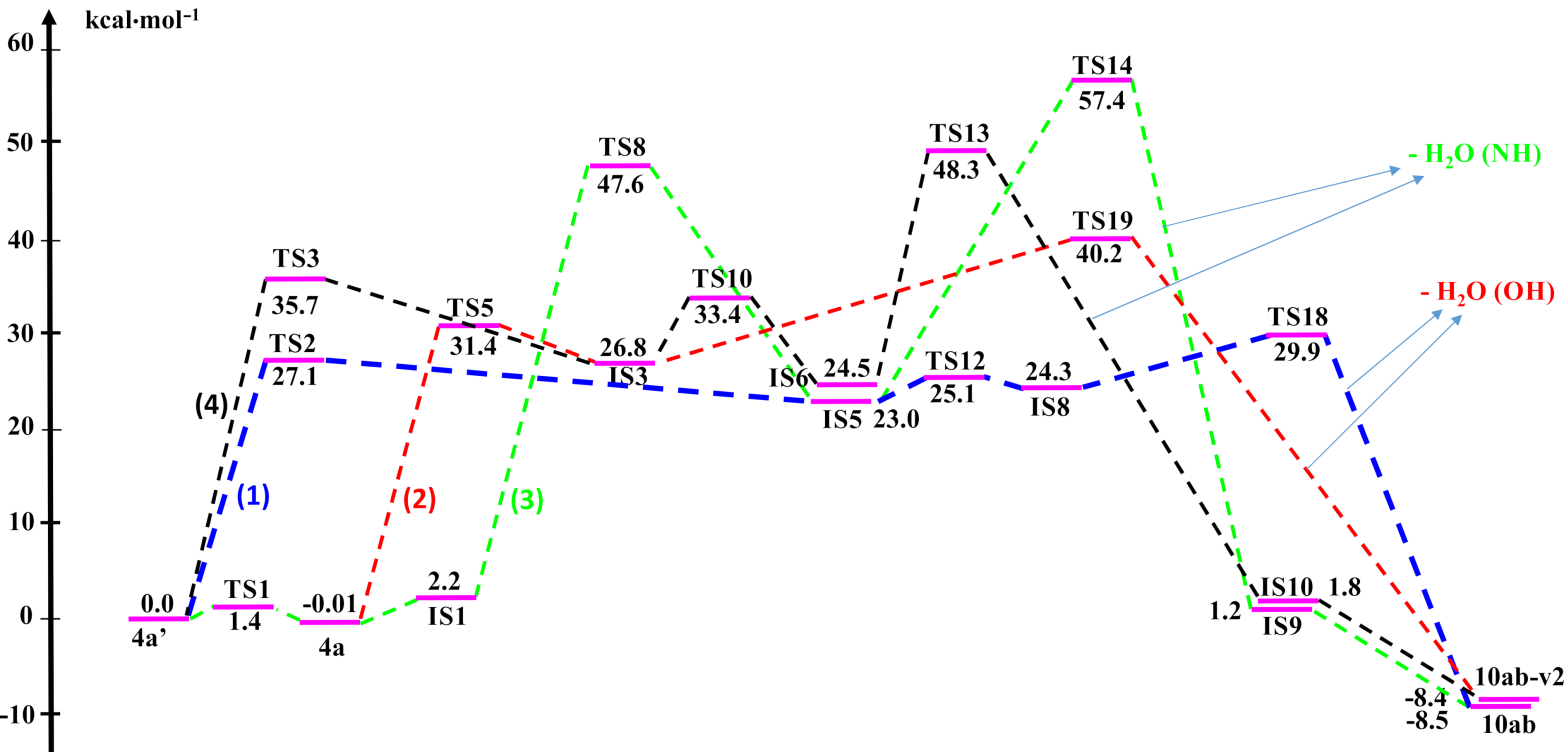
The PES for the possible pathways (1), (2), (3), and (4) in ethanol solvent.

**Figure 6:**
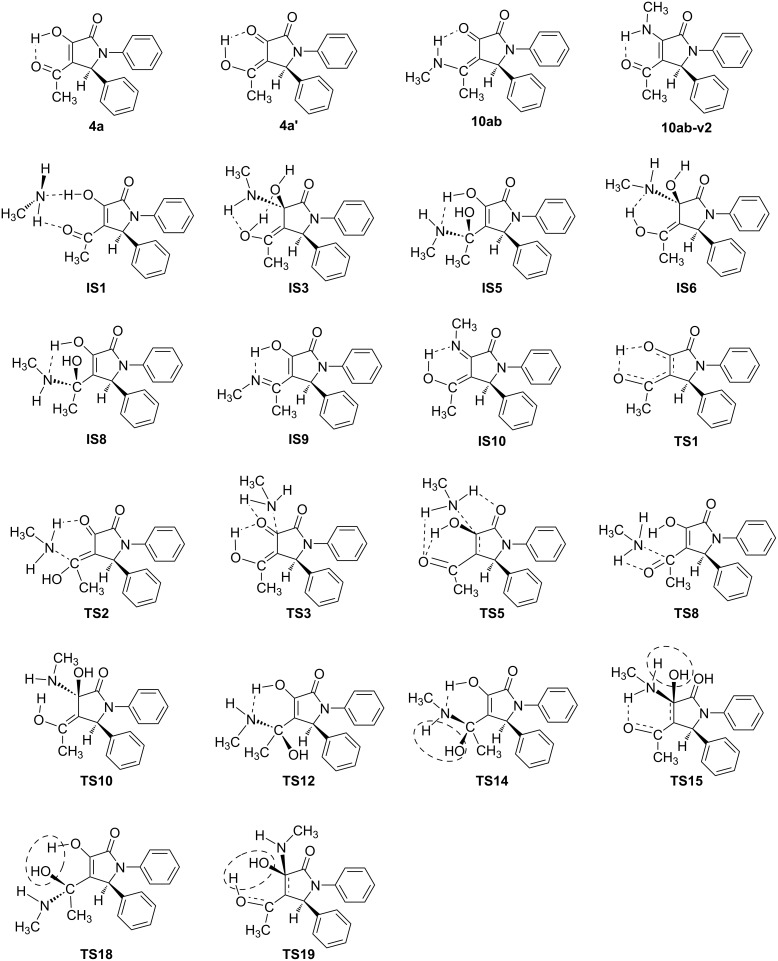
The optimized structures of some reactants, intermediates, transition states, and products in the possible pathways.

In order to better evaluate the reaction mechanism of **10ab** synthesis, we continued to investigate four possible reaction pathways (1–4) corresponding to the green, blue, red, and black lines on the PES in different solvents, including ethanol, chloroform and dimethyl sulfoxide (DMSO) at the same level of theory. Especially, ethanol is the solvent used experimentally for the synthesis of **10ab**. The calculated results in the solvent model are shown in Figure 5, Table 3, and Table S3 in Supporting Information File 1, and the stable structures are further illustrated in Figure 6 and Figure S7 (see Supporting Information File 1).

Notably, comparing the reaction pathways and the energy values illustrated in Figure 5 (ethanol solvent model) with that in Figure 4 (gas phase), most of the directions take place with similar trends. In fact, the most preferred way is from **4a’** to create **IS5** and finally to form **10ab** by removal of one H_2_O molecule from two –OH groups close together (blue line). Another competitive direction is from **4a** to **IS3** and finally to **10ab-v2** by the release of one H_2_O molecule from O–H and N–H groups. Noticeably, in the ethanol solvent model, the formation of **10ab** and **10ab-v2** is thermodynamically similar (the Δ*G* difference is minimal, about 0.1 kcal·mol^−1^). In this case, the kinetic factor mainly determines the predominant product. With a difference of potential barrier of ca. 4.4 kcal·mol^−1^ in the first stage (to form intermediate), or 7.8 kcal·mol^−1^ in the final stage (for H_2_O separation) (**IS5** isomerization to yield **IS8** can be omitted because it is so fast with a small activation energy of 2.1 kcal·mol^−1^), the formation of product **10ab** is superior as compared to its isomer. In terms of the value of the rate constant *k*, the direction of **10ab** formation is in the range of 10^3^–10^6^ times faster than **10ab-v2**. The remaining two reaction routes are to form the intermediates **IS9**, **IS10** with the separation of a H_2_O molecule from the O–H and N–H bonds resulting in **10ab** and **10ab-v2**, respectively, which are evaluated lower than the above two pathways in releasing H_2_O molecule from two –OH groups. Hence, from the calculated results in the solvent model, it could be confirmed that, in terms of thermodynamic and kinetic aspects, compound **10ab** is the most preferred product as compared to others for the reaction between **4a/4a’** with CH_3_NH_2_. In addition, removing one H_2_O molecule from two –OH groups in the intermediates is more favorable than from –OH and –NH groups. Moreover, the reaction mechanism in the chloroform and DMSO solvent model is also considered to confirm the main reaction pathway (Table S3 in Supporting Information File 1). The results indicate that the same trends and slight differences of free energy values are shown in solvent models used in this work.

## Conclusion

4-Acetyl-3-hydroxy-3-pyrrolin-2-ones were synthesized successfully via the three-component reaction of an aromatic aldehyde, aniline, and ethyl 2,4-dioxovalerate with acceptable yields. The broadening of peaks in the ^13^C NMR spectra for the two carbon atoms of the acetyl group is due to the tautomerism of 4-acetyl-3-hydroxy-3-pyrrolin-2-ones in DMSO. The isomers **4a** and **4a’** exist together upon synthesis with the significantly large rate constant of transformation of **4a** into **4a’** of ca. 10^12^ s^−1^ in the gas phase and solvents mode (ethanol, chloroform, DMSO).

The reaction between 4-acetyl-3-hydroxy-3-pyrrolin-2-ones and an aliphatic amine in ethanol occurs at the carbonyl group attached to the 4-position of the heterocyclic ring to obtain 1,4,5-trisubstituted pyrrolidine-2,3-dione enamine derivatives. Remarkably, the theoretical results demonstrate that the possible pathway to form product **10ab** from **4a**/**4a’** is the direction (1) following the PES in the gas phase and the ethanol/CHCl_3_/DMSO solvents model. It can be suggested that **10ab** is the most favorable product as a result of the reaction process via transition state **TS2** and intermediate **IS5**, in good agreement with the experimental results. Besides, the kinetic selectivity is more significant than the thermodynamic one for forming the main product **10ab** with a rate constant ≈ 10^3^–10^6^ times higher (from the first step to the final step) in ethanol solvent (also in chloroform, DMSO) as compared to **10ab-v2**. Moreover, CH_3_NH_2_ approaches **4a**/**4a’** at the opposite side of the C–C_6_H_5_ sites to form **IS3**/**IS5** and finally **10ab-v2**/**10ab** . The reaction mechanisms in the gas phase and in the solvents model are only slightly different.

## Experimental

### General experimental methods

NMR spectra were acquired on commercial instruments (Bruker Avance 300 MHz, Bruker Avance II+ 500 MHz, or Bruker Avance II+ 600 MHz) and chemical shifts (δ) are reported in parts per million (ppm) referenced to tetramethylsilane (TMS) or the internal (NMR) solvent signals. For column chromatography, 70–230 mesh silica 60 (E. M. Merck) was used as the stationary phase. Chemicals received from commercial sources were used without further purification. Melting points (not corrected) were determined using a Büchi Melting Point B-545. Exact mass measurements were acquired on a quadrupole orthogonal acceleration time-of-flight mass spectrometer (Synapt G2 HDMS, Waters, Milford, MA). Samples were infused at 3 μL/min and spectra were obtained in positive (or negative) ionization mode with a resolution of 15000 (FWHM) using leucine enkephalin as lock mass. Besides, exact mass measurements were also recorded on a SCIEX X500 QTOF with electrospray ionization (ESI) source in a positive mode. The temperatures of the source were set at 300 °C. Curtain gas (25 psi) chambers were filled with high-purity nitrogen. The capillary voltage was constantly kept at 5500 V. Collision energies was set at 10 V and zero collision energy spread. IDA mode was used to find mass in range (100 to 1000).

### Computational methods

In this work, DFT calculations were used to investigate the synthesis of 1,4,5-trisubstituted pyrrolidine-2,3-dione (**10ab**) from 2-pyrrolidinone (**4a/4a’**) and methylamine (**9b**, CH_3_NH_2_). The stable structures of reactants, intermediates, transition states, and products were optimized at the B3LYP/6-31+G(d,p) level of theory by Gaussian 09 package [48]. The vibrational frequencies of these structures were also computed at the same level to determine the minima and transition states on the potential energy surface. Further, some highly possible pathways of forming **10ab** were considered in ethanol, chloroform, and dimethyl sulfoxide (DMSO) solvents, in which ethanol was used in the experimental work, based on the Polarizable Continuum Model (PCM) method [49]. Similar to the calculations in the gas phase, the geometrical structures were optimized, and vibrational frequencies were computed at the same level in the solvent model. Especially, the single-point energy values of structures wee examined at the higher level B3LYP/6-311++G(2df,2pd) using the optimized geometries at B3LYP/6-31+G(d,p) in the gas phase and solvents. The intrinsic reaction coordinates (IRCs) were performed at the same level of theory to determine two local minima through transition states in each stepwise [50]. Besides, the rate constant of reaction based on the transitional state theory method and quantum tunneling effect was calculated by the following expression [47]:


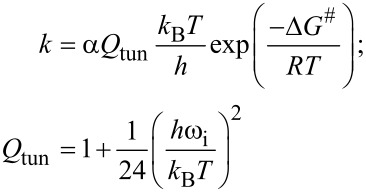


with *Q*_tun_, Δ*G*^#^, ω_i_ are tunneling transmission coefficient, Gibbs free energy values, and imaginary frequencies of transition states, respectively.

## Supporting Information

File 1 Experimental part, additional DFT data and NMR spectra.
