# Supplementary material for: Experimental and theoretical studies on the synthesis of 1,4,5-trisubstituted pyrrolidine-2,3-diones
Source: Beilstein J Org Chem. 2022 Aug 31;18:1140–53. doi: 10.3762/bjoc.18.118 (PMC9443309; doi:10.3762/bjoc.18.118)
Supplement: File 1 — Experimental part, additional DFT data and NMR spectra. [file Beilstein_J_Org_Chem-18-1140-s001.pdf]

BEILSTEIN JOURNAL OF ORGANIC CHEMISTRY

Supporting Information

for

Experimental and theoretical studies on the synthesis of 1,4,5-trisubstituted pyrrolidine-2,3-diones

Nguyen Tran Nguyen, Vo Viet Dai, Nguyen Ngoc Tri, Luc Van Meervelt,
Nguyen Tien Trung and Wim Dehaen

Beilstein J. Org. Chem. **2022,** *18,* 1140–1153. doi:10.3762/bjoc.18.118

Experimental part, additional DFT data and NMR spectra

License and Terms: This is a supporting information file under the terms of the Creative Commons Attribution License (https://creativecommons.org/licenses/by/4.0). Please note that the reuse, redistribution and reproduction in particular requires that the author(s) and source are credited and that individual graphics may be subject to special legal provisions.
The license is subject to the *Beilstein Journal of Organic Chemistry* terms and conditions: (https://www.beilstein-journals.org/bjoc/terms)

Table of contents

1. Experimental procedures and characterization data S1

2. Crystal structure determination of **10aa** S14

3. Reaction pathways from **4a** to **10ab-v3** via **IS4** in gas phase S17

4. Thermodynamic parameters and the structures of reactants, intermediates,

transitional states, products shown in PES S18

5. Copies of NMR spectra and ESI-HRMS spectra of all reported compounds S23

1. Experimental procedures and characterization data

General procedure for the synthesis of 4-acetyl-3-hydroxy-3-pyrrolin-2-ones 4a–c: To a round-bottomed flask equipped with a mechanical stirrer was added benzaldehyde (1.5 equiv), aniline (1.0 equiv), and glacial acetic acid. The mixture was stirred at room temperature for 1 hour under Ar atmosphere and the imine formation was monitored by TLC (hexane/EtOAc 5:1). Then, ethyl 2,4-dioxovalerate (1.0 equiv) was added, the resulting solution was stirred vigorously at room temperture under Ar atmosphere for 2–4 hours, and the formation of 3-hydroxy-3-pyrrolin-2-one derivatives was followed by TLC (CH_2Cl_2/CH_3OH 5:0.2). Then, distilled water was added to the flask and the mixture was stirred for 15 minutes. The precipitate was filtered and then crude product was recrystallized from the solvent mixture of CH_2Cl_2 and toluene to obtain the pure compounds.

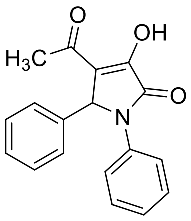

4-Acetyl-3-hydoxy-1,5-diphenyl-3-pyrrolin-2-one (4a): benzaldehyde (0.075 mL, 1.5 equiv, 0. 75 mmol), aniline (0.046 mL, 1.0 equiv, 0.5 mmol), ethyl 2,4-dioxovalerate (0.07 mL, 1.0 equiv, 0.5 mmol), and glacial acetic acid (1.0 mL), under Ar atmosphere. Reaction time is 3 h. The crude was purified *via* recrystallization from a solvent mixture of CH_2Cl_2 and toluene to obtain **4a** (117.6 mg, 80% yield) as a white solid. m.p. 222-223 ˚C.

^{1}H NMR (500 MHz, DMSO-d6) δ 7.58 (d, ^3J(H,H) = 8 Hz, 2H; Ar-H), 7.29 (t, ^3J(H,H) = 7.74 Hz, 2H; Ar-H), 7.23 (d, ^3J(H,H) = 7.42 Hz, 2H; Ar-H), 7.20 (t, ^3J(H,H) = 7.32 Hz, 2H; Ar-H), 7.15 – 7.08 (m, 2H; Ar-H), 2.34 ppm (s, 3H; CH_3). **^{13}C NMR** (100 MHz, DMSO-d6) δ 164.58, 136.93, 136.20, 128.67, 128.10, 127.64, 125.39, 122.49, 120.53, 60.46, 39.11 ppm. **HRMS** (ESI-quadrupole) *m/z* $[M + H]^+$ calcd for $C_{18}H_{15}NO_3$: 294.1130; found: 294.1120

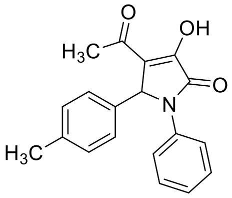

4-Acetyl-3-hydroxy-1-phenyl-5-(*p*-tolyl)-3-pyrrolin-2-one (4b): *p*-tolualdehyde (0.09 mL, 1.5 equiv, 0.75 mmol), aniline (0.046 mL, 1.0 equiv, 0.5 mmol), ethyl 2,4-dioxovalerate (0.07 mL, 1.0 equiv, 0.5 mmol), and glacial acetic acid (1.0 mL), under Ar atmosphere. Reaction time is 3 h. The crude product was purified *via* recrystallization from a solvent mixture of CH_2Cl_2 and toluene to obtain **4b** (106.1 mg, 69% yield) as a white solid. m.p. 206-208 ˚C. **^{1}H NMR** (400 MHz, DMSO-d6) δ 7.56 (dd, ^4J(H,H) = 1.36 Hz, ^3J(H,H) = 8.68 Hz, 2H; Ar-H), 7.28 (t, ^3J(H,H) = 7.36 Hz, 2H; Ar-H), 7.12 – 7.06 (m, 3H; Ar-H), 6.98 (d, ^3J(H,H) = 7.84 Hz, 2H; Ar-H), 2.31 (s, 3H; CH_3), 2.15 ppm (s, 3H; CH_3). **^{13}C NMR** (100 MHz, DMSO-d6) δ 191.83 (*broad peak*), 164.54, 136.82, 136.23, 133.81, 128.72, 128.65, 127.50, 125.32, 122.45, 120.62, 60.20, 30.13 (*broad peak*), 20.60 ppm. **HRMS** (ESI-quadrupole) *m/z* $[M + H]^+$ calcd for $C_{19}H_{17}NO_3$: 308.1287; found: 308.1274

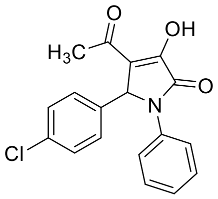

4-Acetyl-3-hydroxy-5-(4-chlorophenyl)-1-phenyl-3-pyrrolin-2-one (4c): From 4-chlorobenzaldehyde (105.4 mg, 1.5 equiv, 0.75 mmol), aniline (0.046 mL, 1.0 equiv, 0.5 mmol), ethyl 2,4-dioxovalerate (0.07 mL, 1.0 equiv, 0.5 mmol), and acetic acid (1.0 mL), under Ar atmosphere. Reaction time is 5 h. The crude product was purified *via* recrystallization from a solvent mixture of CH_2Cl_2 and toluene to obtain **4c** (139.2 mg, 85% yield) as a white solid. m.p. 193-194 ˚C.

^{1}H NMR (500 MHz, DMSO-d6) δ 7.55 (d, ^3J(H,H) = 7.37 Hz, 2H; Ar-H), 7.29 (d, ^3J(H,H) = 8.24 Hz, 2H; Ar-H), 7.27 (s, 2H; Ar-H), 7.23 (d, ^3J(H,H) = 8.59 Hz, 2H; Ar-H), 9.09 (t, ^3J(H,H) = 7.42 Hz, 1H; Ar-H), 2.32 (s, 3H;CH_3). **^{13}C NMR** (125 MHz, DMSO-d6) δ 191.84, 164.49, 136.12, 136, 132.12, 129.57, 128.72, 128.10, 125.50, 122.49, 120.17, 59.70, 30.08 ppm.

^{1}H NMR (500 MHz, $CDCl_3$) δ 7.39 (dd, ^4J(H,H) = 1.46 Hz, ^3J(H,H) = 8.59 Hz, 2H; Ar-H), 7.29 (t, ^3J(H,H) = 7.83 Hz, 2H; Ar-H), 7.23 (d, ^3J(H,H) = 8.48 Hz, 2H; Ar-H), 7.18 – 7.13 (m, 3H, Ar-H), 2.25 ppm (s, 3H, CH_3). **^{13}C NMR** (125 MHz, $CDCl_3$) δ 194.44, 163.78, 135.58, 134.60, 133.68, 129.16, 129.14, 129.01, 128.98, 128.20, 126.53, 125.27, 122.71, 119.75, 61.64, 29.04 ppm. **HRMS** (ESI-TOF MS/MS) *m/z* $[M + H]^+$ calcd for $C_{18}H_{14}ClNO_3$: 328.0740; found: 328.0735

General procedure for the reaction between 4-acetyl-3-hydroxy-3-pyrrolin-2-ones 4a–c and aliphatic amines: To a screw-capped reaction tube equipped with a magnetic stirring bar was added 4-acetyl-3-hydroxy-3-pyrrolin-2-one (1.0 equiv) and aliphatic amine (4 equiv). Subsequently, ethanol was added and the resulting mixture was stirred at 80 °C for 5 h. The consumption of starting materials and the formation of product was monitored by TLC (CH_2Cl_2/CH_3OH 5:0.1). Then, hexane (5 mL) was added to the tube and the mixture was stirred at room temperature for 15 minutes. The precipitate was purified by column chromatography (silica gel, eluent CH_2Cl_2/CH_3OH). In some cases, recrystallization was used for further purification after column chromatography.

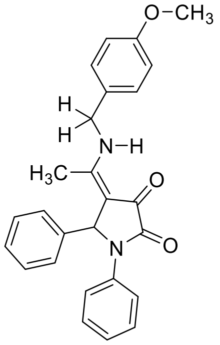

4-[1-(4-Methoxybenzyl)amino]ethylidene-1,5-diphenylpyrrolidine-2,3-dione (10aa): mixture of 4-acetyl-3-hydoxy-1,5-diphenyl-3-pyrrolin-2-one (**4a**, 50.0 mg, 1.0 equiv, 0.17 mmol), 4-methoxybenzylamine (**9a**, 0.091 mL, 4.0 equiv, 0.68 mmol), and ethanol (0.34 mL) was stirred vigorously at 80 °C for 5 h. The crude product was purified by column chromatography (silica gel, eluent CH_2Cl_2/CH_3OH 5:0.03) affording **10aa** (62.6 mg, 89% yield) as an off white solid. m.p. 208-211 ˚C.

^{1}H NMR (400 MHz, $CDCl_3$) δ 11.74 (t_{br}, ^3J(H,H) = 6.11 Hz, 1H; N−H), 7.43 (dd, ^4J(H,H) = 1.44 Hz, ^3J(H,H) = 8.56 Hz, 2H; Ar-H), 7.32 – 7.22 (m, 7H; Ar-H), 7.19 (d, ^3J(H,H) = 8.66 Hz, 2H; Ar-H), 7.14 (t, ^3J(H,H) = 7.48 Hz, 1H; Ar-H), 6.92 (d, ^3J(H,H) = 8.63 Hz, 2H; Ar-H), 5.75 (s, 1H), 4.48 (dd, ^2J(H,H) = 6.11 Hz, ^3J(H,H) = 15.56 Hz, 1H; CH_2), 4.39 (dd, ^2J(H,H) = 6.21 Hz, ^3J(H,H) = 15.56 Hz, 1H; CH_2), 3.85 (s, 3H; OCH_3), 1.87 ppm (s, 3H; CH_3). **^{13}C NMR** (100 MHz, $CDCl_3$) δ 177.24, 164.34, 163.15, 159.42, 138.69, 136.81, 128.87, 128.83, 128.40, 128.35, 128.28, 128.12, 128.09, 126.21, 124.01, 114.54, 107.37, 61.55, 55.45, 46.77, 29.81, 15.61 ppm. **HRMS** (ESI-quadrupole) *m/z* $[M + H]^+$ calcd for $C_{26}H_{24}N_2O_3$: 413.1865; found: 413.1856

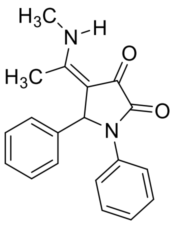

4-(1-Methylamino)ethylidene-1,5-diphenylpyrrolidine-2,3-dione (10ab): a mixture of 4-acetyl-3-hydoxy-1,5-diphenyl-3-pyrrolin-2-one (**4a**, 50.0 mg, 1.0 equiv, 0.17 mmol), methylamine (**9b**, 0.24 mL, 4.0 equiv, 0.68 mmol), and ethanol (0.34 mL) was stirred vigorously at 80 °C for 5 h. The precipitate was recrystallized from a solvent mixture of CH_2Cl_2 and hexane to obtain pure product **10ab** (46.0 mg, 88%) as an off white solid. m.p. 284-286 ˚C. **^{1}H NMR (**400 MHz, $CDCl_3$) δ 11.31 (d_{br}, ^3J(H,H) = 7.02 Hz, 1H; N−H), 7.48 (dd, ^4J(H,H) = 1.37 Hz, ^3J(H,H) = 8.59 Hz, 2H; Ar-H), 7.34 – 7.22 (m, 7H; Ar-H), 7.14 (t, ^3J(H,H) = 7.34 Hz, 1H; Ar-H), 5.78 (s, 1H), 2.90 (d, ^3J(H,H) = 5.32 Hz, 3H; CH_3), 1.86 ppm (s, 3H; CH_3). **^{13}C NMR** (100 MHz, $CDCl_3$) δ 176.66, 165.40, 164.60, 138.77, 136.86, 128.87, 128.80, 128.36, 128.14, 126.10, 124.02, 107.14, 61.39, 30.00, 15.35 ppm. **HRMS** (ESI-quadrupole) *m/z* $[M + H]^+$ calcd for $C_{19}H_{18}N_2O_2$: 307.1447; found: 307.1436

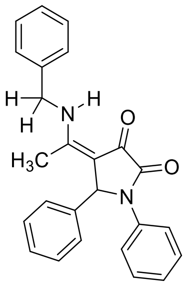

4-(1-Benzylamino)ethylidene-1,5-diphenylpyrrolidine-2,3-dione (10ac): a mixture of 4-acetyl-3-hydoxy-1,5-diphenyl-3-pyrrolin-2-one (**4a**, 50.0 mg, 1.0 equiv, 0.17 mmol), benzylamine (**9c**, 0.074 mL, 4.0 equiv, 0.68 mmol), and ethanol (0.34 mL) was stirred vigorously at 80 °C for 5 h. The crude product was purified by column chromatography (silica gel, eluent CH_2Cl_2/CH_3OH 5:0.07) affording **10ac** (58.7 mg, 90% yield) as an off white solid. m.p. 235-237 ˚C.

¹H NMR (500 MHz, $CDCl_3$) δ 11.66 (s_{br}, 1H; N−H), 7.28 (t, ^3J(H,H) = 7.79 Hz, 2H; Ar-H), 7.23 – 7.10 (m, 11H; Ar-H), 7.01 (t, ^3J(H,H) = 7.36 Hz, 1H; Ar-H), 5.64 (s, 1H), 4.44 (dd, ^2J(H,H) = 5.91 Hz, ^3J(H,H) = 15.89 Hz, 1H; CH_2), 4.37 (dd, ^2J(H,H) = 6.16 Hz, ^3J(H,H) = 15.87 Hz, 1H; CH_2), 1.73 ppm (s, 3H; CH_3). **¹³C NMR** (125 MHz, $CDCl_3$) δ 177.54, 164.28, 164.19, 138.71, 136.83, 136.24, 129.20, 128.92, 128.88, 128.47, 128.12, 126.88, 126.32, 124.11, 107.47, 61.67, 47.28, 15.62 ppm. **HRMS** (ESI-TOF MS/MS) *m/z* $[M + H]^+$ calcd for $C_{25}H_{22}N_2O_2$: 383.1760; found: 383.1756

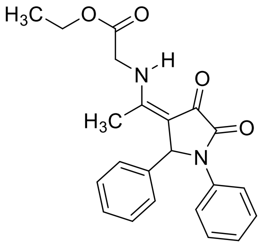

4-[1-((Ethoxycarbonylmethyl)amino)]ethylidene-1,5-diphenylpyrrolidine-2,3-dione (10ad): a mixture of 4-acetyl-3-hydoxy-1,5-diphenyl-3-pyrrolin-2-one (**4a**, 50.0 mg, 1.0 equiv, 0.17 mmol), glycine ethyl ester hydrochloride (**9d**, 94.9 mg, 4.0 equiv, 0.68 mmol), and ethanol (0.34 mL) was stirred vigorously at 80 °C for 5 h. The crude product was purified by column chromatography (silica gel, eluent CH_2Cl_2/CH_3OH 5:0.07) affording **10ad** (53.1 mg, 82% yield) as an off white solid. m.p. 215-217 ˚C.

¹H NMR (500 MHz, $CDCl_3$) δ 11.34 (s_{br}, 1H; N−H), 7.29 (dd, ^4J(H,H) = 1.34 Hz, ^3J(H,H) = 8.44 Hz, 1H; Ar-H), 7.19 – 7.11 (m, 8H; Ar-H), 7.03 (t, ^3J(H,H) = 7.47 Hz, 1H; Ar-H), 5.62 (s, 1H), 4.15 (q, ^3J(H,H) = 7.08 Hz, 2H; O−CH_2), 3.97 (dd, ^2J(H,H) = 6.05 Hz, ^3J(H,H) = 18.11 Hz, 1H; CH_2), 3.89 (dd, ^2J(H,H) = 6 Hz, ^3J(H,H) = 18.1 Hz, 1H; CH_2), 1.70 (s, 3H; CH_3), 1.21 ppm (t, ^3J(H,H) = 7.13 Hz, 3H; CH_3). **¹³C NMR** (125 MHz, $CDCl_3$) δ 178.42, 167.86, 163.92, 163.60, 138.58, 136.77, 128.95, 128.91, 128.53, 128.17, 126.43, 124.23, 107.59, 62.27, 61.66, 44.99, 15.60, 14.25 ppm. **HRMS** (ESI-TOF MS/MS) *m/z* $[M + H]^+$ calcd for $C_{22}H_{22}N_2O_4$: 379.1658; found: 379.1661

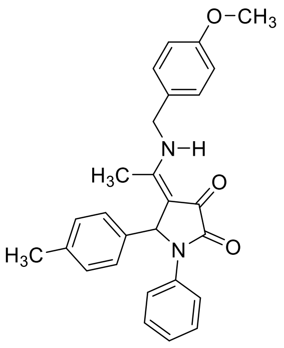

5-(4-Methylphenyl)-4-[1-(4-methoxybenzyl)amino]ethylidene-1-phenylpyrrolidine-2,3-dione (10ba): 4-acetyl-3-hydroxy-1-phenyl-5-(*p*-tolyl)-3-pyrrolin-2-one (**4b**, 50.0 mg, 1.0 equiv, 0.163 mmol), 4-methoxybenzylamine (**9a**, 0.087 mL, 4.0 equiv, 0.652 mmol), and ethanol (0.34 mL) was stirred vigorously at 80 °C for 4 h. The crude product was purified by column chromatography (silica gel, eluent CH_2Cl_2/CH_3OH 5:0.08) affording **10ba** (52.9 mg, 76% yield) as an off white solid. m.p. 209-211 ˚C.

^{1}H NMR (600 MHz, $CDCl_3$) δ 11.66 (t_{br}, ^3J(H,H) = 6.31 Hz, 1H; N−H), 7.37 (dd, ^4J(H,H) = 1.12 Hz, ^3J(H,H) = 8.62 Hz, 2H; Ar-H), 7.23 (t, ^3J(H,H) = 7.44 Hz, 2H; Ar-H), 7.12 (d, ^3J(H,H) = 8.61 Hz, 2H; Ar-H), 7.09 – 7.05 (m, 3H; Ar-H), 6.99 (d, ^3J(H,H) = 7.60 Hz, 2H; Ar-H), 6.86 99 (d, ^3J(H,H) = 8.67 Hz, 2H; Ar-H), 5.66 (s, 1H), 4.42 (dd, ^2J(H,H) = 6.09 Hz, ^3J(H,H) = 15.54 Hz, 1H; CH_2), 4.34 (dd, ^2J(H,H) = 6.21 Hz, ^3J(H,H) = 15.54 Hz, 1H; CH_2), 3.78 (s, 3H; O−CH_3), 2.22 (s, 3H; CH_3), 1.80 ppm (s, 3H; CH_3). **^{13}C NMR** (150 MHz, $CDCl_3$) δ 177.31, 164.35, 164.07, 159.43, 138.14, 136.90, 135.57, 129.53, 128.84, 128.29, 128.18, 127.97, 126.14, 124.00, 114.55, 107.45, 61.28, 55.46, 46.77, 21.21, 15.58 ppm. **HRMS** (ESI-quadrupole) *m/z* $[M + H]^+$ calcd for $C_{27}H_{26}N_2O_3$: 427.2022; found: 427.2019

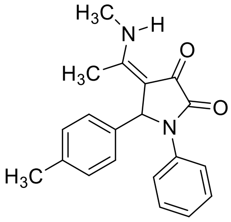

5-(4-Methylphenyl)-4-(1-methylamino)ethylidene-1-phenylpyrrolidine-2,3-dione (10bb): 4-acetyl-3-hydroxy-1-phenyl-5-(*p*-tolyl)-3-pyrrolin-2-one (**4b**, 50.0 mg, 1.0 equiv, 0.163 mmol), methylamine (**9b**, 0.23 mL, 4.0 equiv, 0.65 mmol), and ethanol (0.34 mL) was stirred vigorously at 80 °C for 4 h. The crude product was recrystallized from a solvent mixture of CH_2Cl_2 and hexane to afford **10bb** (44.0 mg, 84% yield) as an off white solid. m.p. 272-274 ˚C. **^{1}H NMR (**400 MHz, $CDCl_3$) δ 11.19 (d_{br}, ^3J(H,H) = 7.06 Hz, 1H; N−H), 7.45 (d, ^3J(H,H) = 7.40 Hz, 2H; Ar-H), 7.29 – 7.23 (m, 2H; Ar-H), 7.07 – 7.05 (m, 3H; Ar-H), 6.98 (d, ^3J(H,H) = 7.76 Hz, 2H; Ar-H), 5.71 (s, 1H), 2.75 (d, ^3J(H,H) = 5.19 Hz, 3H; CH_3), 2.22 (s, 3H; CH_3), 1.78 ppm (s, 3H; CH_3). **^{13}C NMR** (100 MHz, $CDCl_3$) δ 176.44, 165.64, 164.63, 137.99, 136.90, 135.57, 129.43, 128.78, 128.00, 125.89, 123.90, 107.19, 60.91, 29.85, 21.18, 15.35 ppm. **HRMS** (ESI-quadrupole) *m/z* $[M + H]^+$ calcd for $C_{20}H_{20}N_2O_2$: 321.1603; found: 321.1588

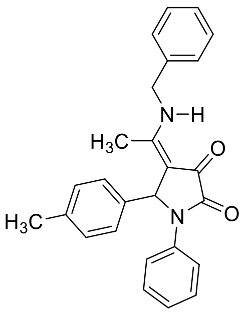

5-(4-Methylphenyl)-4-(1-benzylamino)ethylidene-1-phenylpyrrolidine-2,3-dione (10bc): 4-acetyl-3-hydroxy-1-phenyl-5-(*p*-tolyl)-3-pyrrolin-2-one (**4b**, 50.0 mg, 1.0 equiv, 0.163 mmol), benzylamine (**9c**, 0.071 mL, 4.0 equiv, 0.652 mmol), and ethanol (0.34 mL) was stirred vigorously at 80 °C for 4 h. The crude product was purified by column chromatography (silica

gel, eluent CH_2Cl_2/CH_3OH 5:0.04) affording **10bc** (52.1 mg, 81% yield) as an off white solid. m.p. 250-251 ˚C.

^{1}H NMR (500 MHz, $CDCl_3$) δ 11.73 (t_{br}, ^{3}J(H,H) = 6.26 Hz, 1H; N−H), 7.38 (dd, ^{4}J(H,H) = 1.37 Hz, ^{3}J(H,H) = 8.66 Hz, 2H; Ar-H), 7.33 (d, ^{3}J(H,H) = 7.72 Hz, 2H; Ar-H), 7.30 – 7.20 (m, 5H; Ar-H), 7.10 – 7.06 (m, 3H; Ar-H), 7.00 (d, ^{3}J(H,H) = 7.86 Hz, 2H; Ar-H), 5.68 (s, 1H), 4.50 (dd, ^{2}J(H,H) = 6.13 Hz, ^{3}J(H,H) = 15.88 Hz, 1H; CH_2), 4.43 (dd, ^{2}J(H,H) = 6.36 Hz, ^{3}J(H,H) = 15.89 Hz, 1H; CH_2), 2.23 (s, 3H; CH_3), 1.80 ppm (s, 3H; CH_3). **^{13}C NMR** (125 MHz, $CDCl_3$) δ 177.59, 164.27, 164.15, 138.19, 136.90, 136.30, 135.57, 129.56, 129.18, 128.87, 128.09, 127.98, 126.86, 126.19, 124.04, 107.52, 61.32, 47.22, 21.23, 15.57 ppm. **HRMS** (ESI-TOF MS/MS) *m/z* $[M + H]^+$ calcd for $C_{26}H_{24}N_2O_2$: 397.1916; found: 397.1916

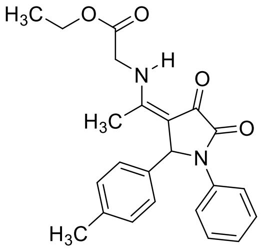

5-(4-Methylphenyl)-4-[1-((ethoxycarbonylmethyl)amino)]ethylidene-1-phenylpyrrolidine-2,3-dione (10bd): 4-acetyl-3-hydroxy-1-phenyl-5-(*p*-tolyl)-3-pyrrolin-2-one (**4b**, 50.0 mg, 1.0 equiv, 0.163 mmol), glycine ethyl ester hydrochloride (**9d**, 91.0 mg, 4.0 equiv, 0.652 mmol), and ethanol (0.34 mL) was stirred vigorously at 80 °C for 4 h. The crude product was purified by column chromatography (silica gel, eluent CH_2Cl_2/CH_3OH 5:0.1) affording **10bd** (56.0 mg, 87% yield) as an off white solid. m.p. 248-250 ˚C.

^{1}H NMR (500 MHz, $CDCl_3$) δ 11.38 (t_{br}, ^{3}J(H,H) = 6.09 Hz, 1H; N−H), 7.38 (dd, ^{4}J(H,H) = 1.3 Hz, ^{3}J(H,H) = 8.44 Hz, 2H; Ar-H), 7.26 – 7.23 (m, 2H; Ar-H), 7.09 (t, ^{3}J(H,H) = 7.45 Hz, 1H; Ar-H), 7.06 (d, ^{3}J(H,H) = 7.94 Hz, 2H; Ar-H), 6.99 (d, ^{3}J(H,H) = 7.80 Hz, 2H; Ar-H), 5.69 (s, 1H), 4.20 (q, (d, ^{3}J(H,H) = 7.13 Hz, 2H; OCH_2), 4.00 (dd, ^{2}J(H,H) = 6.22 Hz, ^{3}J(H,H) = 18.13 Hz, 1H; CH_2), 3.88 (dd, ^{2}J(H,H) = 5.94 Hz, ^{3}J(H,H) = 18.18 Hz, 1H; CH_2), 2.22 (s, 3H;

CH_3), 1.76 (s, 3H; CH_3), 1.27 ppm (t, ^3J(H,H) = 7.13 Hz, 3H, CH_3). **^{13}C NMR** (125 MHz, $CDCl_3$) δ 178.33, 167.90, 163.96, 163.71, 138.22, 136.82, 135.42, 129.56, 128.89, 128.04, 126.25, 124.11, 107.66, 62.21, 61.24, 44.90, 21.23, 15.61, 14.23 ppm. **HRMS** (ESI-TOF MS/MS) *m/z* [M + H]$^+$ calcd for $C_{23}H_{24}N_2O_4$: 393.1814; found: 393.1806

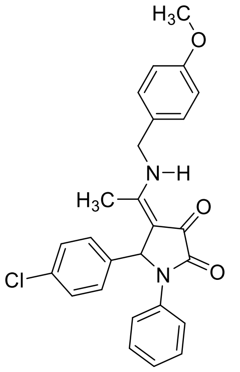

5-(4-Chlorophenyl)-4-[1-(4-methoxybenzyl)amino]ethylidene-1-phenylpyrrolidine-2,3-dione (10ca): 4-acetyl-3-hydroxy-5-(4-chlorophenyl)-1-phenyl-3-pyrrolin-2-one (**4c**, 50.0 mg, 1.0 equiv, 0.153 mmol), 4-methoxybenzylamine (**9a**, 0.081 mL, 4.0 equiv, 0.612 mmol), and ethanol (0.34 mL) was stirred vigorously at 80 °C for 7 h. The crude product was purified by column chromatography (silica gel, eluent CH_2Cl_2/CH_3OH 5:0.1) affording **10ca** (55.0 mg, 81% yield) as a light yellow solid. m.p. 224-227 ˚C.

^{1}H NMR (500 MHz, $CDCl_3$) δ 11.66 (t_{br}, ^3J(H,H) = 6.13 Hz, 1H; N−H), 7.34 (dd, ^4J(H,H) = 1.30 Hz, ^3J(H,H) = 8.39 Hz, 2H; Ar-H), 7.27 – 7.25 (m, 2H; Ar-H), 7.18 (d, ^3J(H,H) = 8.5 Hz, 2H; Ar-H), 7.13 – 7.09 (m, 5H; Ar-H), 6.87 (d, ^3J(H,H) = 7.23 Hz, 2H; Ar-H), 5.69 (s, 1H), 4.43 (dd, ^2J(H,H) = 6.09 Hz, ^3J(H,H) = 15.49 Hz, 1H; CH_2), 4.34 (dd, ^2J(H,H) = 6.19 Hz, ^3J(H,H) = 15.49 Hz, 1H; CH_2), 3.79 (s, 3H; CH_3), 1.80 ppm (s, 3H; CH_3). **^{13}C NMR** (125 MHz, $CDCl_3$) δ 177.20, 164.18, 164.04, 159.53, 137.43, 136.57, 134.24, 129.40, 129.16, 129.05, 128.33, 127.99, 126.48, 124.05, 114.63, 106.96, 60.88, 55.49, 46.88, 15.70 ppm. **HRMS** (ESI-TOF MS/MS) *m/z* [M + H]$^+$ calcd for $C_{26}H_{23}ClN_2O_3$: 447.1475; found: 447.1470

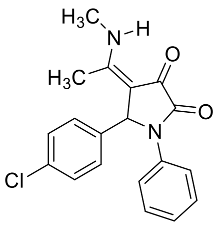

5-(4-Chlorophenyl)-4-(1-methylamino)ethylidene-1-phenylpyrrolidine-2,3-dione (10cb): 4-acetyl-3-hydroxy-5-(4-chlorophenyl)-1-phenyl-3-pyrrolin-2-one (**4c**, 50.0 mg, 1.0 equiv, 0.153 mmol), methylamine (**9b**, 0.22 mL, 4.0 equiv, 0.61 mmol), and ethanol (0.34 mL) was stirred vigorously at 80 °C for 8 h. The crude product was purified by column chromatography (silica gel, eluent CH_2Cl_2/CH_3OH 5:0.15) affording **10cb** (40.3 mg, 77% yield) as a light yellow solid. m.p. 277-280 ˚C.

^{1}H NMR (500 MHz, $CDCl_3$) δ 11.18 (d_{br}, ^{3}J(H,H) = 7.10 Hz, 1H; N−H), 7.42 (d, ^{3}J(H,H) = 7.21 Hz, 2H; Ar-H), 7.28 (t, ^{3}J(H,H) = 7.70 Hz, 2H; Ar-H), 7.17 (d, ^{3}J(H,H) = 8.48 Hz, 2H; Ar-H), 7.12 (d, ^{3}J(H,H) = 8.47 Hz, 2H; Ar-H), 7.10 (t, ^{3}J(H,H) = 7.53 Hz, 2H; Ar-H), 5.73 (s, 3H; CH_3), 2.75 (d, ^{3}J(H,H) = 5.27 Hz, 3H; CH_3), 1.78 ppm (s, 3H; CH_3). **^{13}C NMR** (125 MHz, $CDCl_3$) δ 176.44, 165.57, 165.55, 164.44, 137.47, 136.61, 134.11, 129.46, 129.07, 128.99, 126.23, 123.96, 106.68, 60.53, 29.90, 15.46 ppm. **HRMS** (ESI-TOF MS/MS) *m/z* $[M + H]^+$ calcd for $C_{19}H_{17}ClN_2O_2$: 341.1057; found: 341.1055

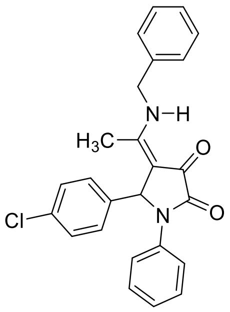

5-(4-Chlorophenyl)-4-(1-benzylamino)ethylidene-1-phenylpyrrolidine-2,3-dione (10cc): 4-acetyl-3-hydroxy-5-(4-chlorophenyl)-1-phenyl-3-pyrrolin-2-one (**4c**, 50.0 mg, 1.0 equiv, 0.153 mmol), benzylamine (**9c**, 0.066 mL, 4.0 equiv, 0.612 mmol), and ethanol (0.34 mL) was

stirred vigorously at 80 °C for 8 h. The crude product was purified by column chromatography (silica gel, eluent CH_2Cl_2/CH_3OH 5:0.1) affording **10cc** (49.7 mg, 79% yield) as an off white solid. m.p. 248-251 ˚C.

^{1}H NMR (600 MHz, $CDCl_3$) δ 11.70 (t_{br}, ^3J(H,H) = 3.03 Hz, 1H; N−H), 7.36 (dd, ^4J(H,H) = 1.18 Hz, ^3J(H,H) = 8.62 Hz, 2H; Ar-H), 7.34 (d, ^3J(H,H) = 7.53 Hz, 2H; Ar-H), 7.30 – 7.24 (m, 3H; Ar-H), 7.19 – 7.09 (m, 7H; Ar-H), 5.71 (s, 1H), 4.48 (dd, ^2J(H,H) = 6.25 Hz, ^3J(H,H) = 15.86 Hz, 1H; CH_2), 4.36 (dd, ^2J(H,H) = 6.25 Hz, ^3J(H,H) = 15.87 Hz, 1H; CH_2), 1.80 ppm (s, 3H; CH_3). **^{13}C NMR** (150 MHz, $CDCl_3$) δ 177.34, 164.28, 164.10, 137.37, 136.54, 136.10, 134.24, 129.40, 129.21, 129.16, 129.05, 128.17, 126.86, 126.48, 124.04, 107.02, 60.84, 47.25, 15.70 ppm. **HRMS** (ESI-TOF MS/MS) *m/z* $[M + H]^+$ calcd for $C_{25}H_{21}ClN_2O_2$: 417.1370; found: 417.1365

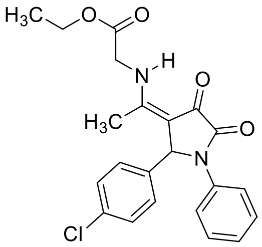

5-(4-Chlorophenyl)-4-[1-((ethoxycarbonylmethyl)amino)]ethylidene-1-phenylpyrrolidine-2,3-dione (10cd): 4-acetyl-3-hydroxy-5-(4-chlorophenyl)-1-phenyl-3-pyrrolin-2-one (**4c**, 50.0 mg, 1.0 equiv, 0.153 mmol), glycine ethyl ester hydrochloride (**9d**, 86.0 mg, 4.0 equiv, 0.618 mmol) was stirred vigorously at 80 °C for 7 h. The crude product was purified by column chromatography (silica gel, eluent CH_2Cl_2/CH_3OH 5:0.06) affording **10cd** (50.4 mg, 80% yield) as a light yellow solid. m.p. 200-203 ˚C.

^{1}H NMR (500 MHz, $CDCl_3$) δ 11.33 (t_{br}, ^3J(H,H) = 6.13 Hz, 1H; N−H), 7.38 (dd, ^4J(H,H) = 1.45 Hz, ^3J(H,H) = 8.47 Hz, 2H; Ar-H), 7.26 (t, ^3J(H,H) = 7.75 Hz, 2H; Ar-H), 7.17 (d, ^3J(H,H) = 8.45 Hz, 2H; Ar-H), 7.13 – 7.09 (m, 3H; Ar-H), 5.73 (s, 1H), 4.19 (q, ^3J(H,H) = 7.12 Hz, 2H; O−CH_2), 3.95 (dd, ^2J(H,H) = 6.37 Hz, ^3J(H,H) = 18.21 Hz, 1H; N−CH_2), 3.74 (dd, ^2J(H,H) =

5.62 Hz, ^3J(H,H) = 18.22 Hz, 1H; N−CH_2), 1.75 (s, 3H; CH_3), 1.26 ppm (t, ^3J(H,H) = 7.13 Hz, 3H; CH_3). **^{13}C NMR** (125 MHz, $CDCl_3$) *δ* 177.86, 167.77, 164.18, 163.83, 137.15, 136.42, 134.26, 129.49, 129.14, 129.05, 126.45, 124.05, 107.19, 66.22, 60.55, 15.82, 14.21. **HRMS** (ESI-TOF MS/MS) *m/z* $[M + H]^+$ calcd for $C_{22}H_{21}ClN_2O_4$: 413.1268; found: 413.1268

2. Crystal structure determination of 10aa

Single crystals of **10aa** suitable for X-ray diffraction were obtained by evaporating a solution of the compound in dichloromethane/*n*-hexane mixture at room temperature. X-ray intensity data were collected at 293(2)K on an Agilent SuperNova diffractometer, equipped with an Eos CCD detector, using Mo Kα radiation (λ = 0.71073 Å). The images were interpreted and integrated with the CrysAlisPro and the implemented absorption correction was applied [1]. Using Olex2 [2], the structure was solved with the ShelXT [3] structure solution program using Intrinsic Phasing and refined with the ShelXL [4] refinement package using full-matrix least-squares minimization on F^2. Non hydrogen atoms were anisotropically refined and the hydrogen atoms in the riding mode with isotropic temperature factors were fixed at 1.2 times U_{eq} of the parent atoms (1.5 for methyl groups). Hydrogen atom H15 was located from a difference electron density map and refined freely. Deposition number CCDC 2174310 contains the supplementary crystallographic data for this paper. These data are provided free of charge by the joint Cambridge Crystallographic Data Centre and Fachinformationszentrum Karlsruhe Access Structures service www.ccdc.cam.ac.uk/structures.

References

1. *CrysAlis PRO*; Rigaku Oxford Diffraction, Yarnton, UK, 2018

2. Dolomanov, O. V.; Bourhis, L. J.; Gildea, R. J.; Howard, J. A. K.; Puschmann, H. *J. Appl. Cryst.* **2009,** *42*, 339-341. doi: 10.1107/S0021889808042726

3. Sheldrick, G. M. *Acta Cryst. A.* **2015,** *71*, 3-8. doi: 10.1107/S2053273314026370

4. Sheldrick, G. M. *Acta Cryst. C*, **2015,** *71*, 3-8. doi: 10.1107/S2053229614024218

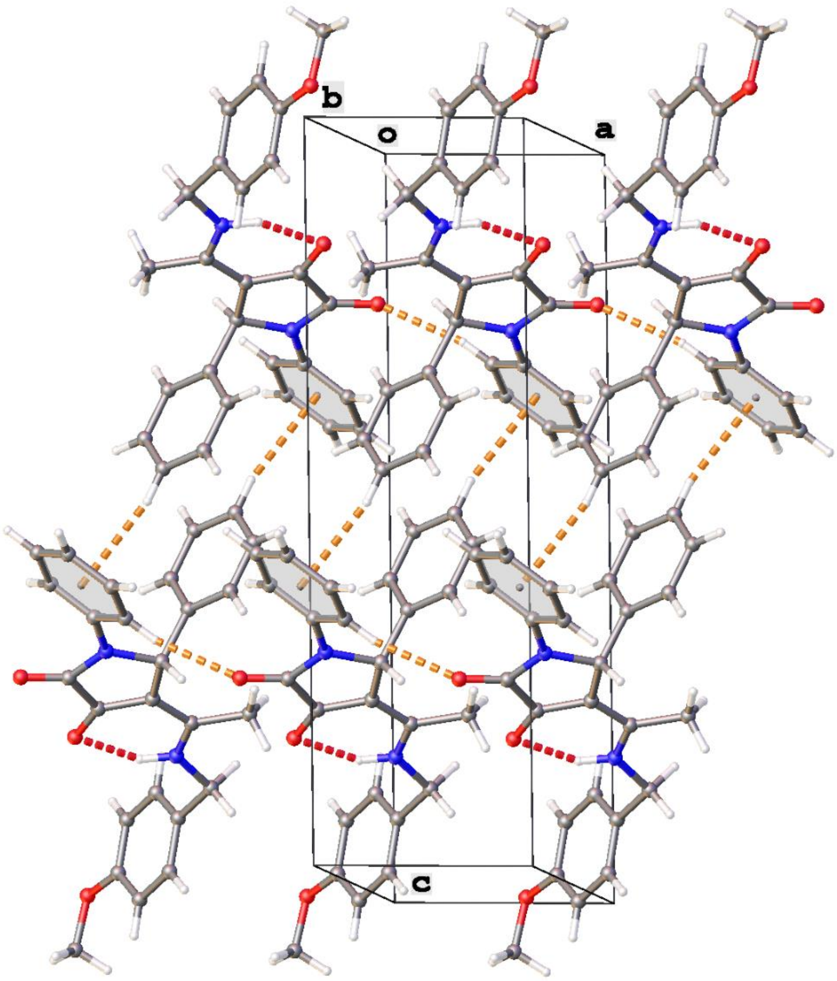

Figure S1. Partial view of the crystal packing of **10aa** showing the chain formation along the *a* direction by C-H···O hydrogen bonds. Neighboring chains connect by C–H···π interactions.

Table S1. Crystal data and structure refinement for **10aa**

Empirical formula	$C_{26}H_{24}N_2O_3$
Formula weight	412.47
Temperature/K	293(2)
Crystal system	triclinic
Space group	P-1
a/Å	6.5031(3)
b/Å	9.1229(3)
c/Å	19.5749(9)
α/°	81.426(3)

β/°	81.347(4)
γ/°	71.352(4)
Volume/$Å^3$	1081.48(8)
Z	2
ρ_{calc}g/cm^3	1.267
μ/mm^{-1}	0.083
F(000)	436.0
Crystal size/mm^3	0.5 × 0.25 × 0.25
Radiation	MoKα (λ = 0.71073)
2Θ range for data collection/°	4.978 to 52.742
Index ranges	-8 ≤ h ≤ 8, -11 ≤ k ≤ 11, -24 ≤ l ≤ 24
Reflections collected	21854
Independent reflections	4424 [R_{int} = 0.0486, R_{sigma} = 0.0347]
Data/restraints/parameters	4424/0/286
Goodness-of-fit on F^2	1.094
Final R indexes [I>=2σ (I)]	R_1 = 0.0697, wR_2 = 0.1731
Final R indexes [all data]	R_1 = 0.0898, wR_2 = 0.1871
Largest diff. peak/hole / e $Å^{-3}$	0.23/-0.23

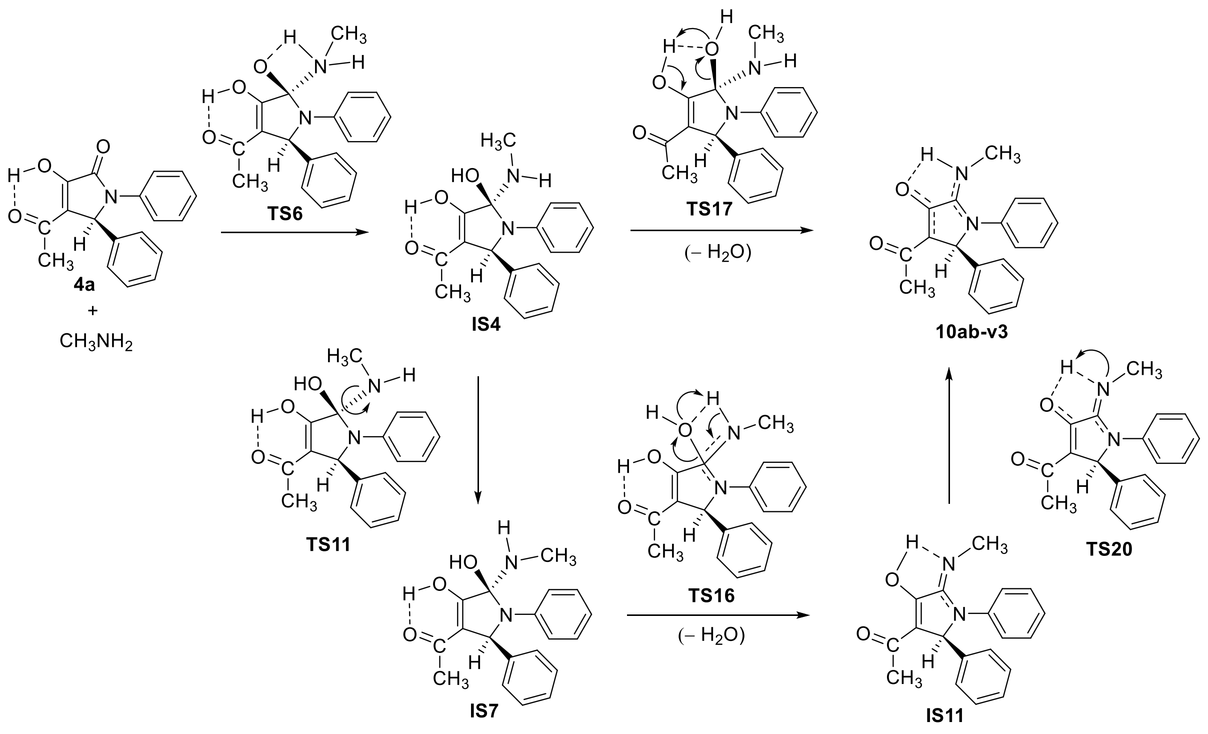

Scheme S1: Reaction pathways from **4a** to **10ab-v3** via **IS4**

Table S2. The relative energies at B3LYP/6-31+G(d,p) (kcal·mol^{-1})

	ΔE		ΔE		ΔE		ΔE
**4a-1**	0.17	**4a'-1**	**0.00**	**10ab-v2-1**	4.09	**IS5-1**	**0.00**
**4a-2**	**0.00**	**4a'-2**	6.88	**10ab-v2-2**	6.21	**IS5-2**	5.42
**4a-3**	0.17	**4a'-3**	6.88	**10ab-v2-3**	6.64	**IS5-3**	1.53
**4a-4**	6.27	**4a'-4**	**0.00**	**10ab-v2-4**	**0.00**	**IS5-4**	4.51
**4a-5**	7.92	**10ab-1**	10.12	**10ab-v2-5**	1.19	**IS5-5**	0.69
**4a-6**	1.30	**10ab-2**	**0.00**	**10ab-v2-6**	3.73	**IS5-6**	1.33
**4a-7**	5.09	**10ab-3**	1.25	**10ab-v2-7**	6.72	**IS5-7**	1.47
**4a-8**	7.92	**10ab-4**	10.12	**10ab-v2-8**	6.64	**IS5-8**	1.66

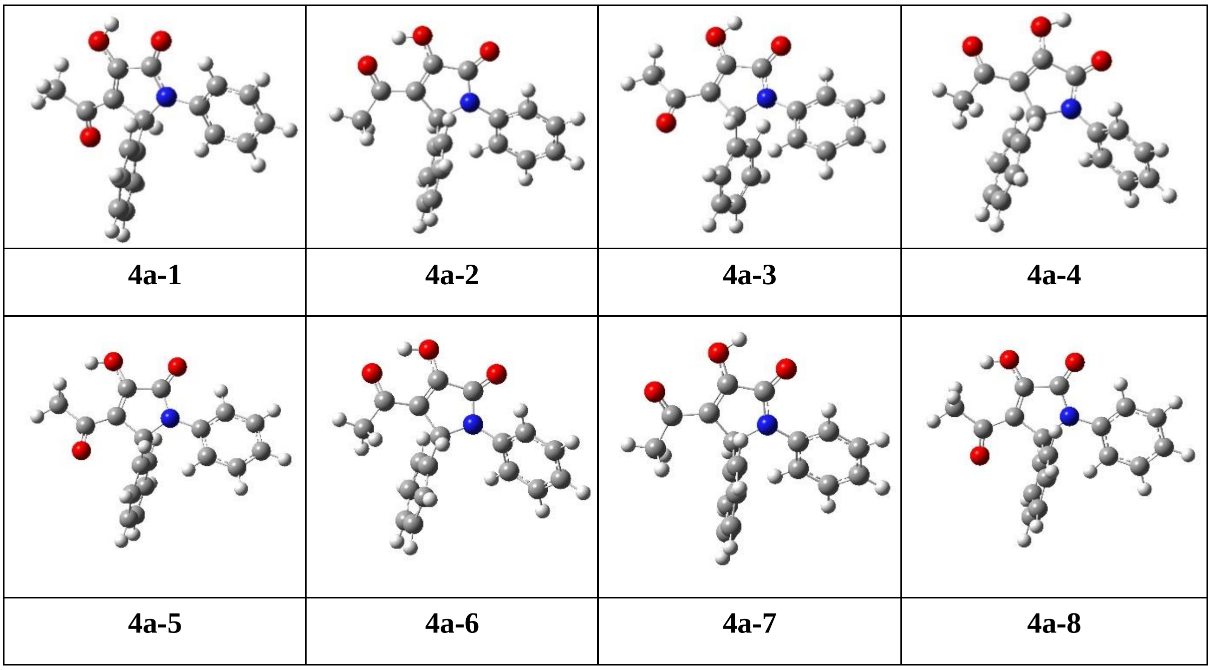

Figure S2. The isomers of **4a**

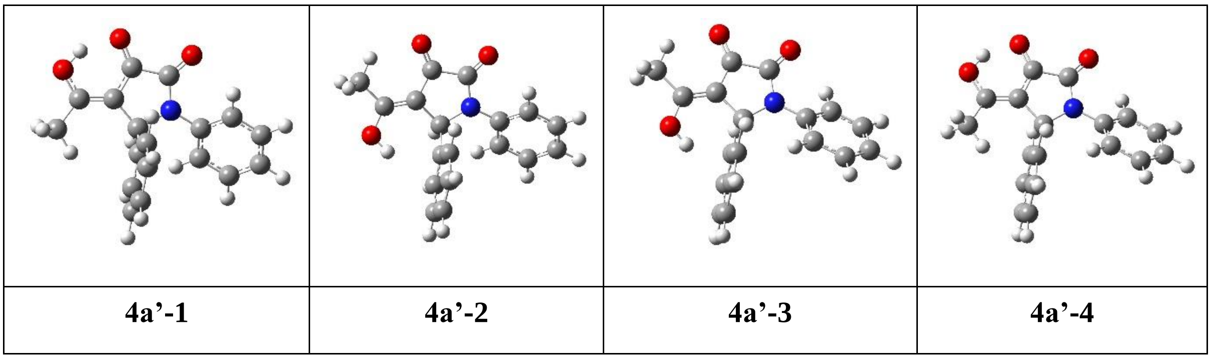

Figure S3. The isomers of **4a'**

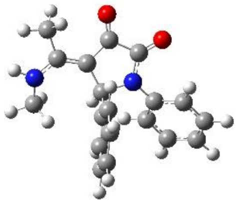	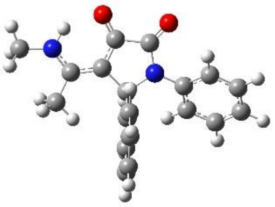	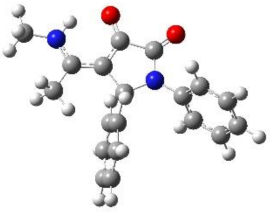	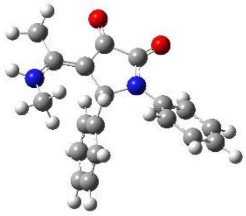
**10ab-1**	**10ab-2**	**10ab-3**	**10ab-4**

Figure S4. The isomers of **10ab**

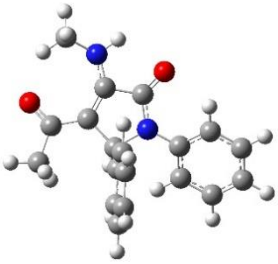	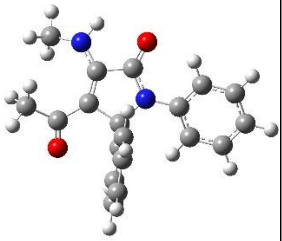	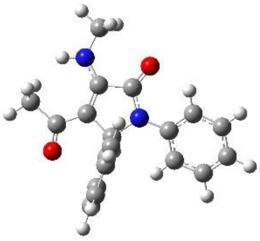	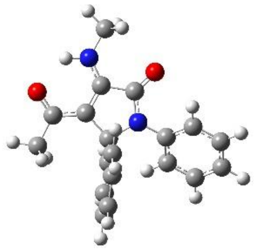
**10ab-v2-1**	**10ab-v2-2**	**10ab-v2-3**	**10ab-v2-4**
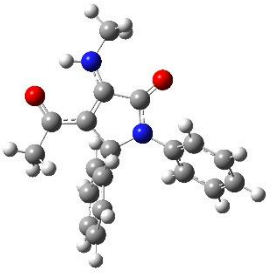	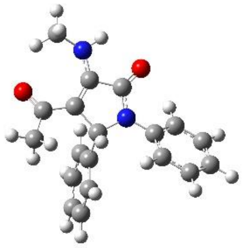	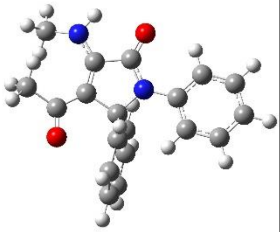	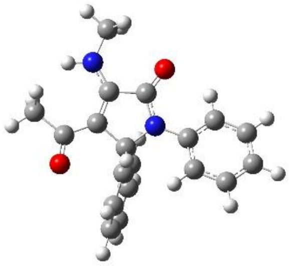
**10ab-v2-5**	**10ab-v2-6**	**10ab-v2-7**	**10ab-v2-8**

Figure S5. The isomers of **10ab-v2**

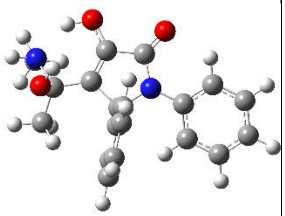	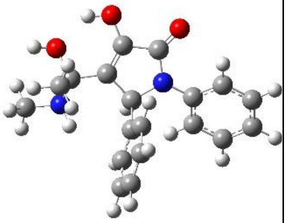	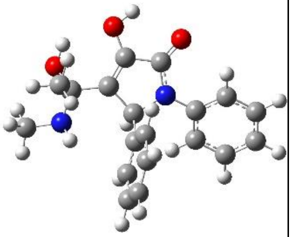	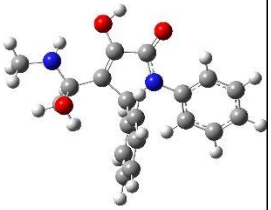
**IS5-1**	**IS5-2**	**IS5-3**	**IS5-4**
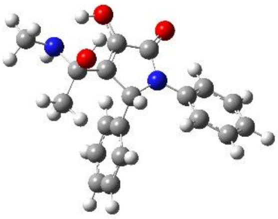	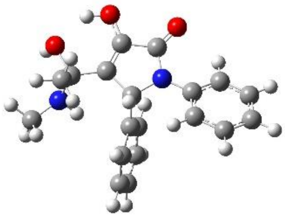	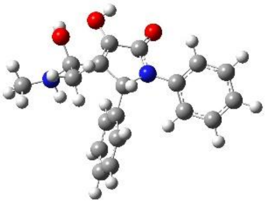	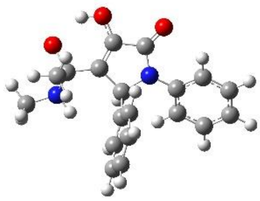
**IS5-5**	**IS5-6**	**IS5-7**	**IS5-8**

Figure S6. The isomers of **IS5**

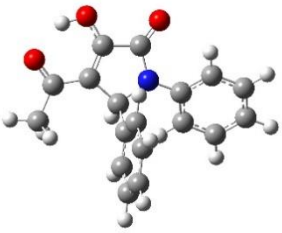	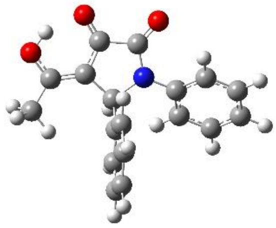	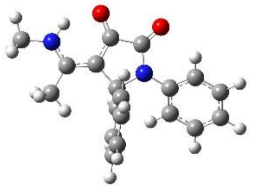	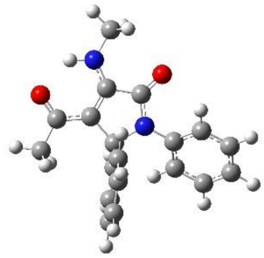
**4a**	**4a’**	**10ab**	**10ab-v2**
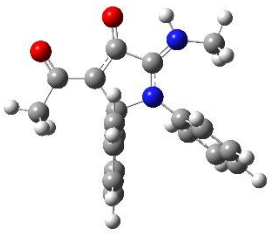	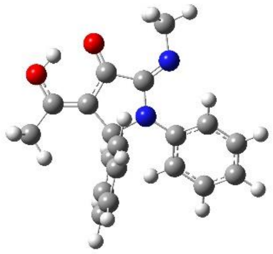	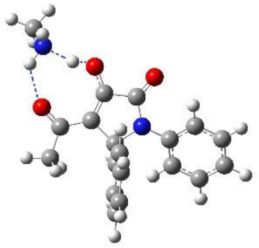	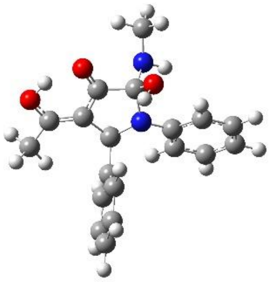
**10ab-v3**	**10ab-v4**	**IS1**	**IS2**
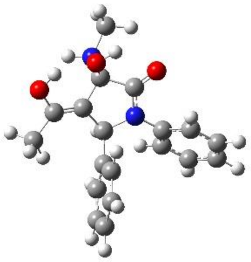	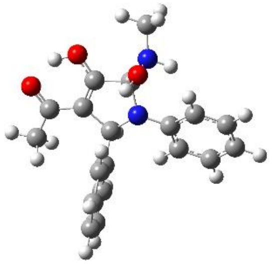	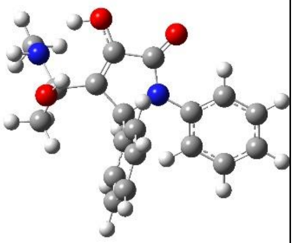	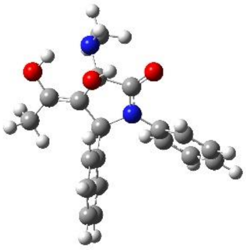
**IS3**	**IS4**	**IS5**	**IS6**
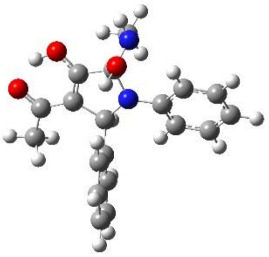	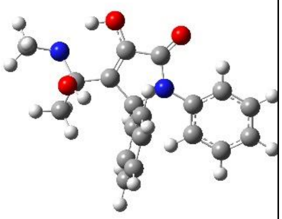	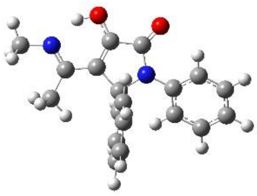	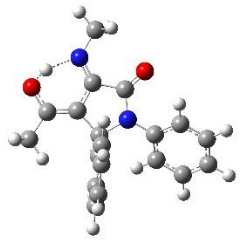
**IS7**	**IS8**	**IS9**	**IS10**

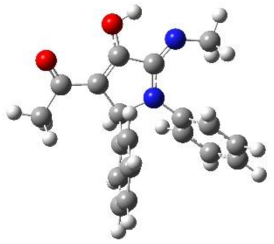	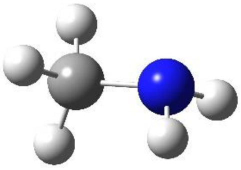	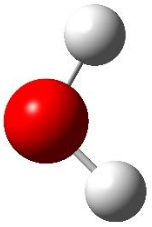	
**IS11**	**9b**	**H_2O**	
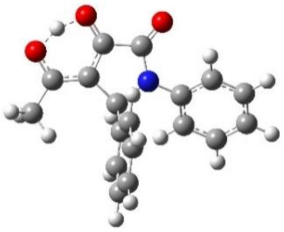	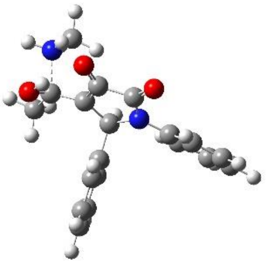	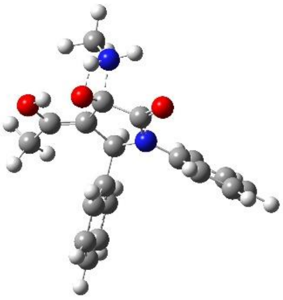	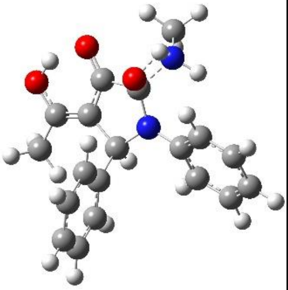
**TS1**	**TS2**	**TS3**	**TS4**
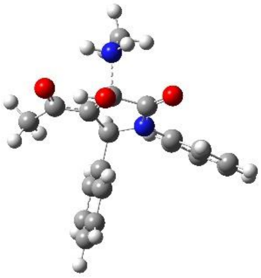	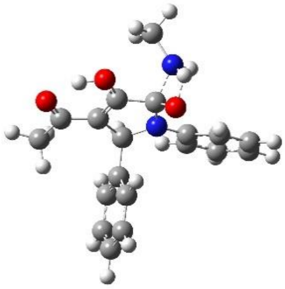	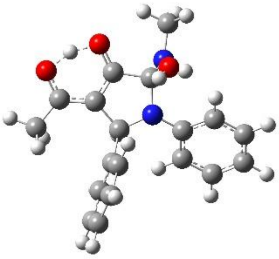	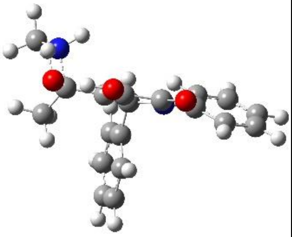
**TS5**	**TS6**	**TS7**	**TS8**
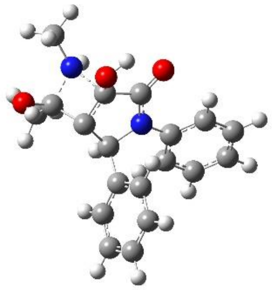	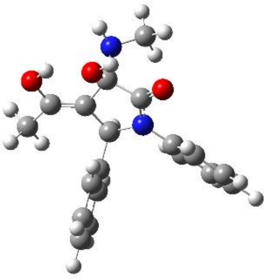	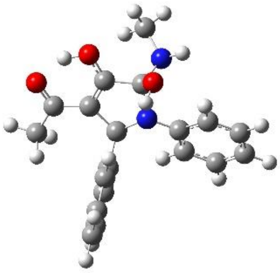	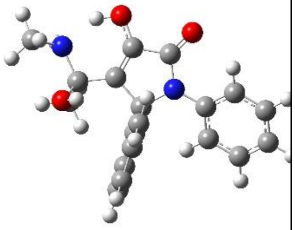
**TS9**	**TS10**	**TS11**	**TS12**
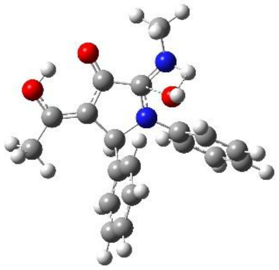	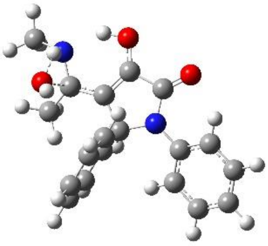	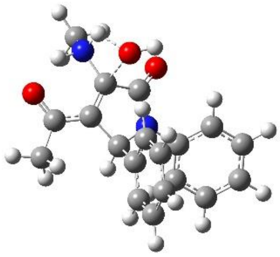	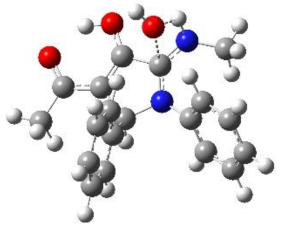

TS13	TS14	TS15	TS16
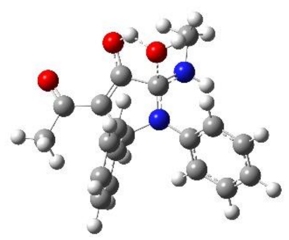	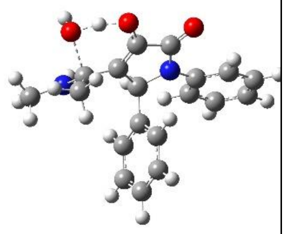	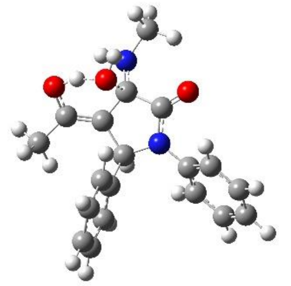	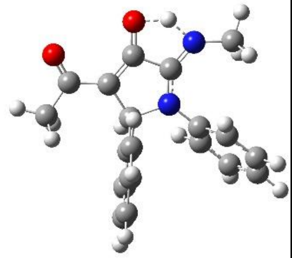
TS17	TS18	TS19	TS20

Figure S7. The structures of reactants, intermediates, transitional states, products shown in PES

Table S3. The thermodynamic parameters of selected pathways (1), (2), (3) and (4) in chloroform ($CHCl_3$) and DMSO (kcal·mol^{-1}) (B3LYP/6-311++g(2d,2p)//B3LYP/6-31+G(d,p))

		**$CHCl_3$**					**DMSO**					**Note**
	**Stage**	**ΔE**	**ΔG**	**ΔE$^{\#}$**	**ΔG$^{\#}$**	**i-Freq**	**ΔE**	**ΔG**	**ΔE$^{\#}$**	**ΔG$^{\#}$**	**i-Freq**	
1	**4a' → 4a**	-0.6	-0.4	1.0	1.5	-1167.79	-0.3	-0.1	1.0	1.2	-1170.31	TS1
2	**4a' → IS5**	23.4	15.8	16.9	30.2	-140.24	25.8	18.8	14.0	26.7	-243.13	TS2
3	**4a' → IS3**	14.1	25.9	38.3	51.2	-1639.37	15.4	27.2	38.4	51.0	-1674.23	TS3
4	**4a → IS1**	-6.1	4.0				-7.5	2.3				
5	**4a → IS3**	13.5	25.5	20.3	32.4	-148.19	15.1	27.1	19.3	31.2	-207.29	TS5
6	**IS1 → IS5**	16.7	19.4	42.3	44.9	-1611.97	18.0	21.0	42.4	45.3	-1653.21	TS8
7	**IS3 → IS6**	-2.4	-1.8	6.3	7.2	-136.62	-2.9	-2.5	5.9	6.5	-135.72	TS10
8	**IS5 → IS8**	1.2	1.2	1.4	2.2	-68.90	1.2	1.0	1.2	1.8	-64.09	TS12
9	**IS5 → IS9**	-10.7	-21.4	35.9	34.9	-465.86	-11.4	-22.2	34.4	33.7	-252.04	TS14
10	**IS6 → IS10**	-11.1	-22.3	26.0	25.5	-273.95	-11.7	-22.7	24.3	23.5	-250.62	TS15
11	**IS8 → 10ab**	-20.6	-31.2	7.5	7.1	-822.59	-22.2	-32.9	6.0	5.6	-730.72	TS18
12	**IS3 → 10ab-v2**	-23.4	-33.8	14.2	14.1	-998.02	-24.8	-35.4	13.3	13.3	-794.97	TS19
13	**IS9 → 10ab**	-8.5	-8.7				-9.6	-9.7				
14	**IS10 → 10ab-v2**	-10.3	-10.3				-10.2	-10.2				

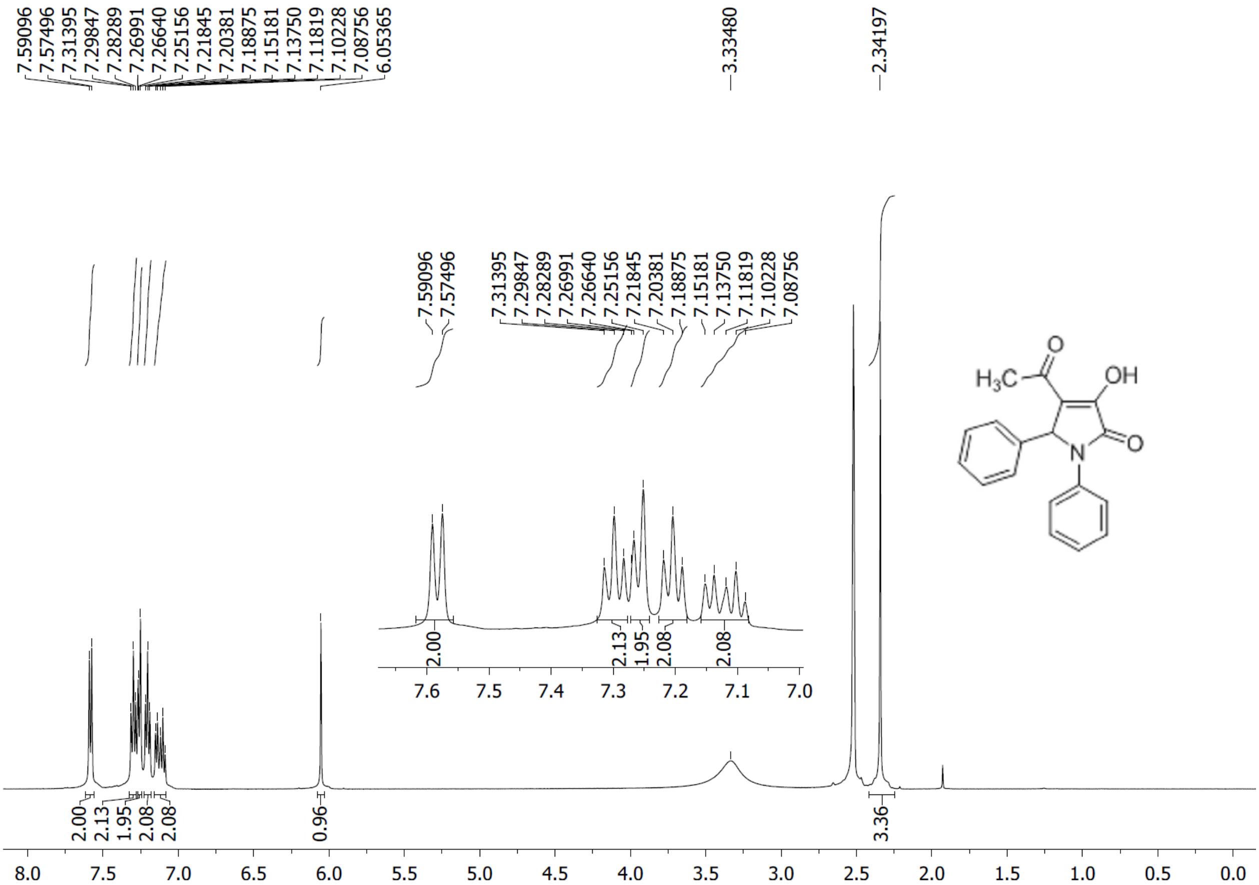
7.59096
7.57496
7.31395
7.29847
7.28289
7.26991
7.26640
7.25156
7.21845
7.20381
7.18875
7.15181
7.13750
7.11819
7.10228
7.08756
6.05365
3.33480
2.34197
2.00
2.13
1.95
2.08
2.08
0.96
3.36
7.6
7.5
7.4
7.3
7.2
7.1
7.0
8.0
7.5
7.0
6.5
6.0
5.5
5.0
4.5
4.0
3.5
3.0
2.5
2.0
1.5
1.0
0.5
0.0
H3C
O
OH
O
N

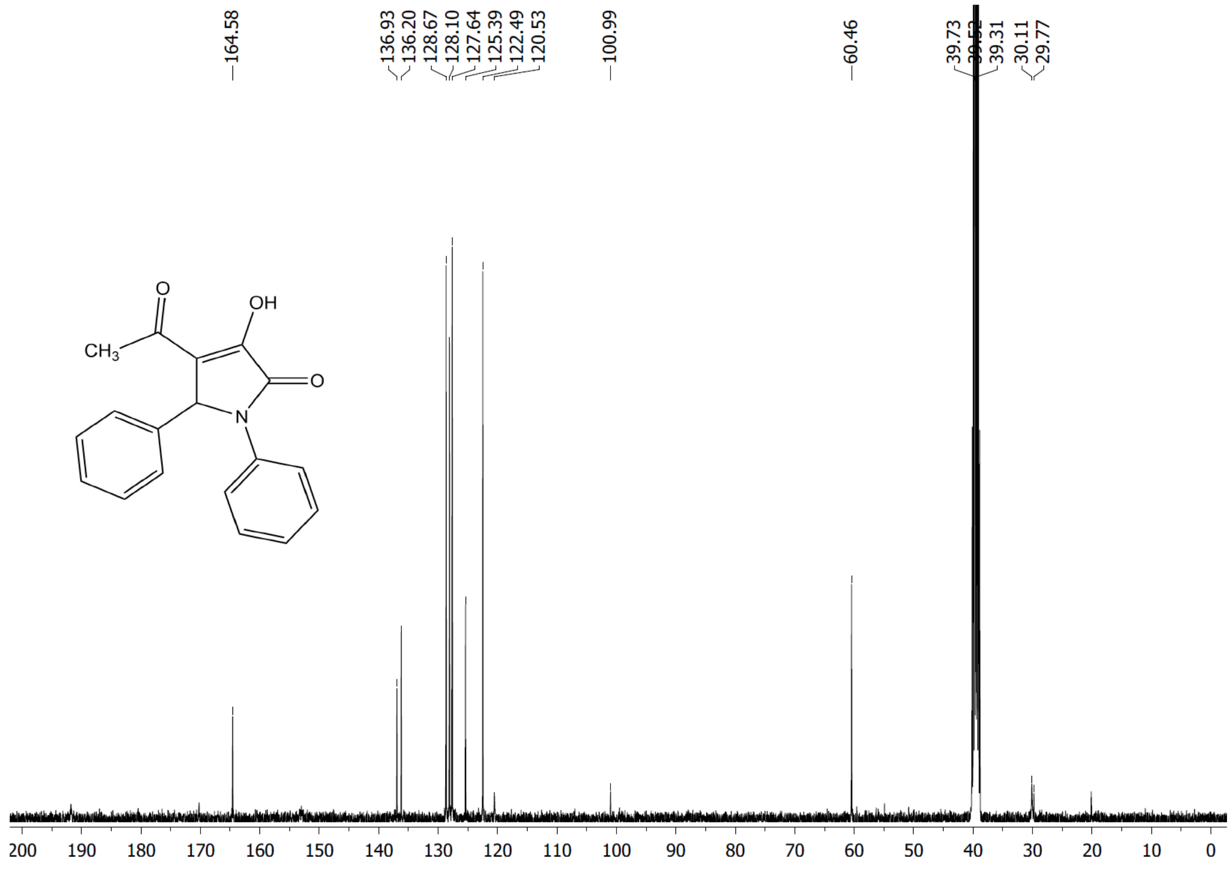
164.58
136.93
136.20
128.67
128.10
127.64
125.39
122.49
120.53
100.99
60.46
39.73
39.31
30.11
29.77
O
OH
CH3
O
N
200
190
180
170
160
150
140
130
120
110
100
90
80
70
60
50
40
30
20
10
0

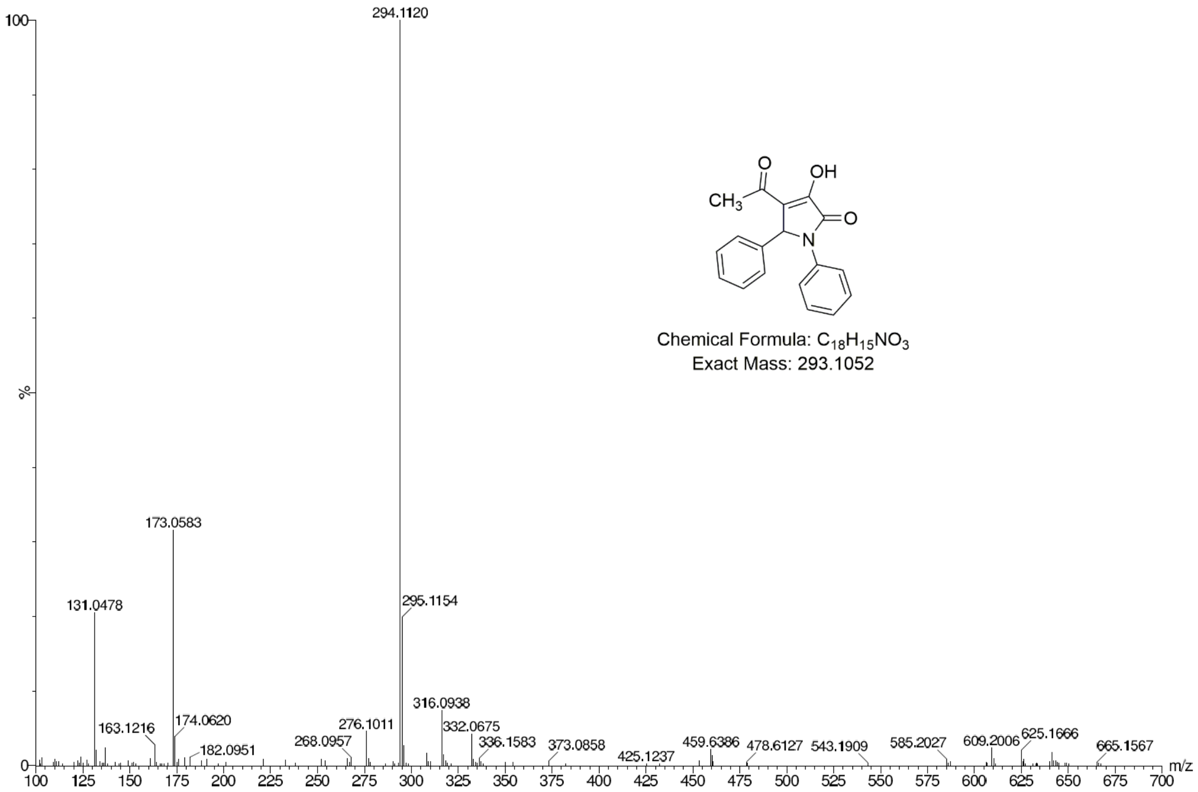

1H NMR spectrum of 4b in DMSO-d6

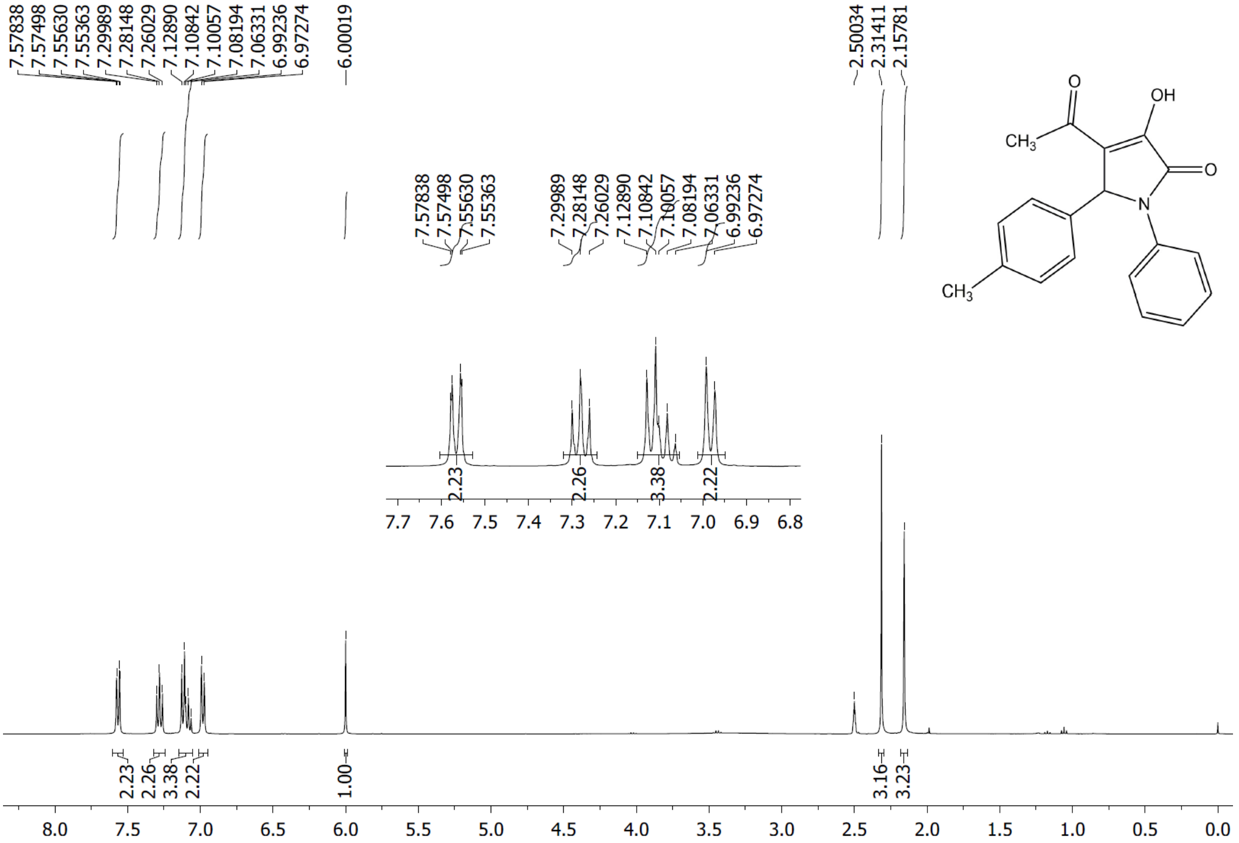

13C NMR spectrum of 4b in DMSO-d6

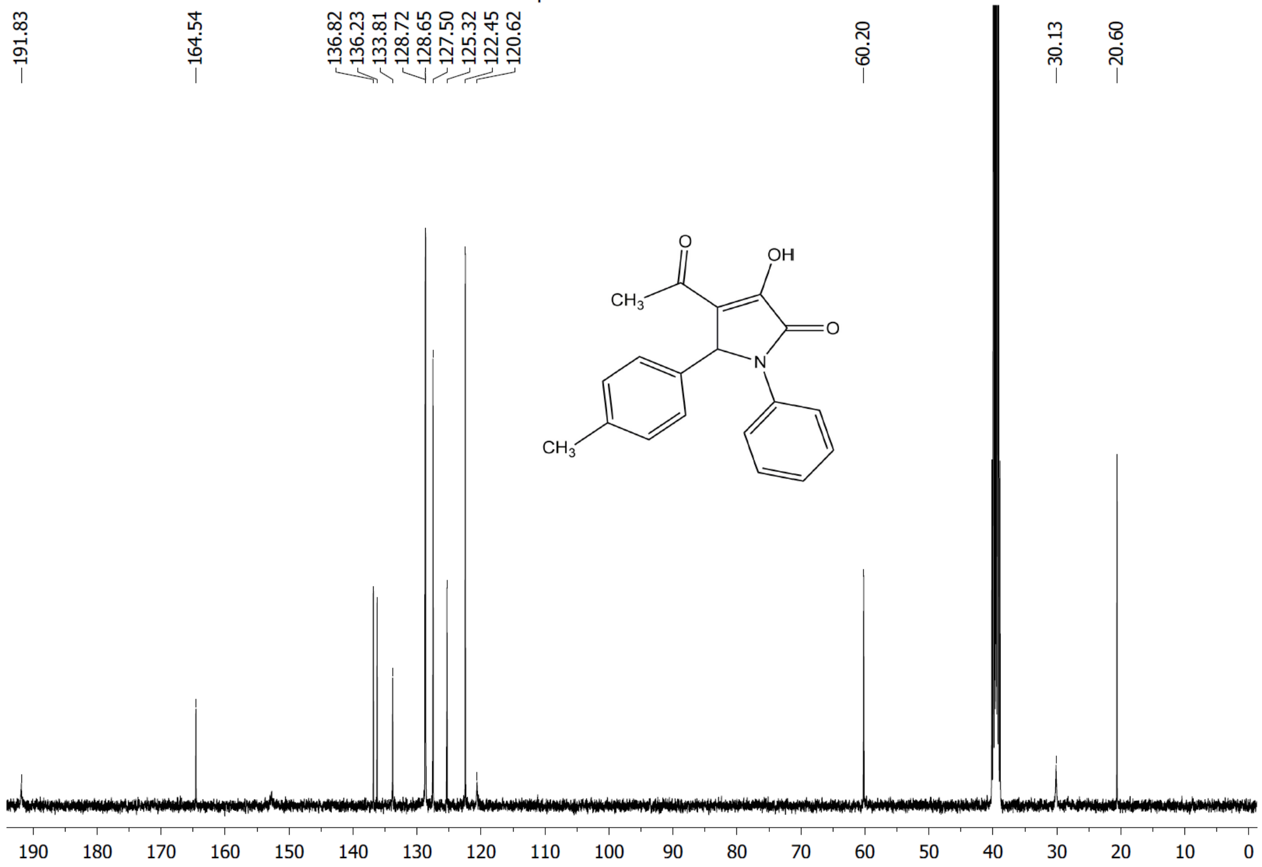

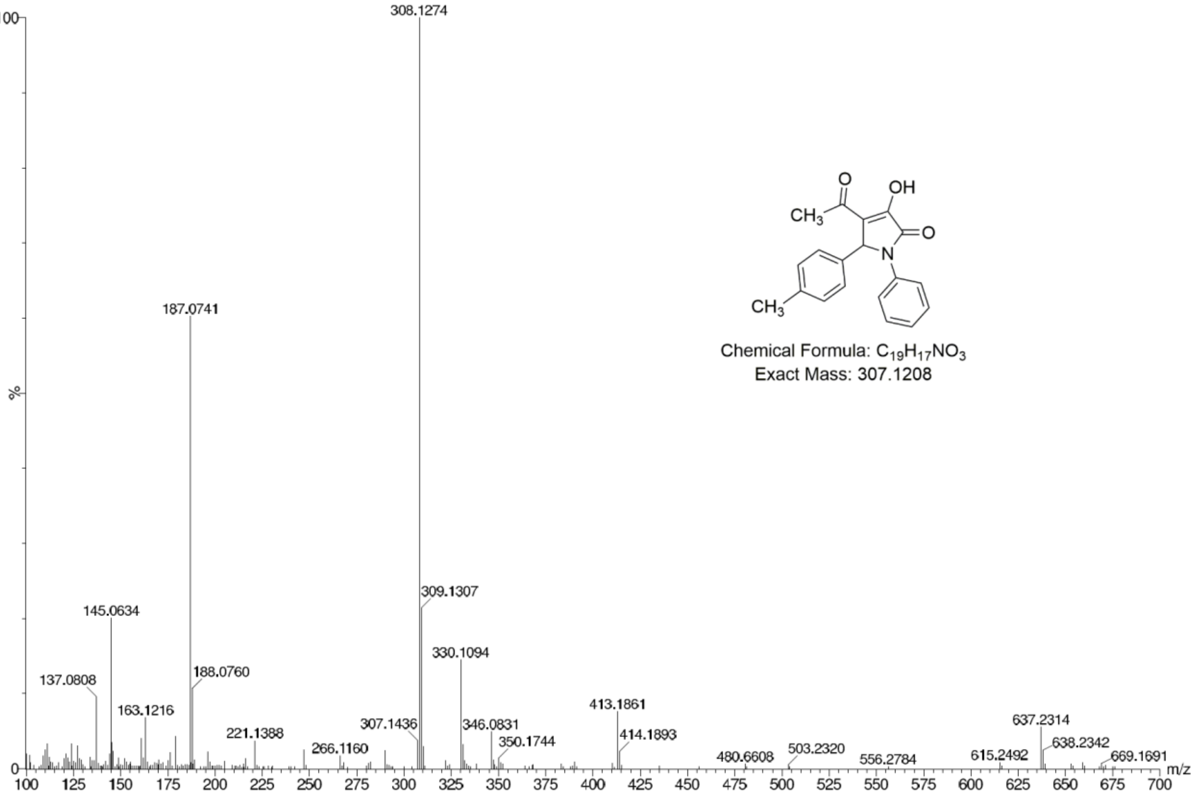

1H NMR spectrum of 4c in DMSO, 500MHz

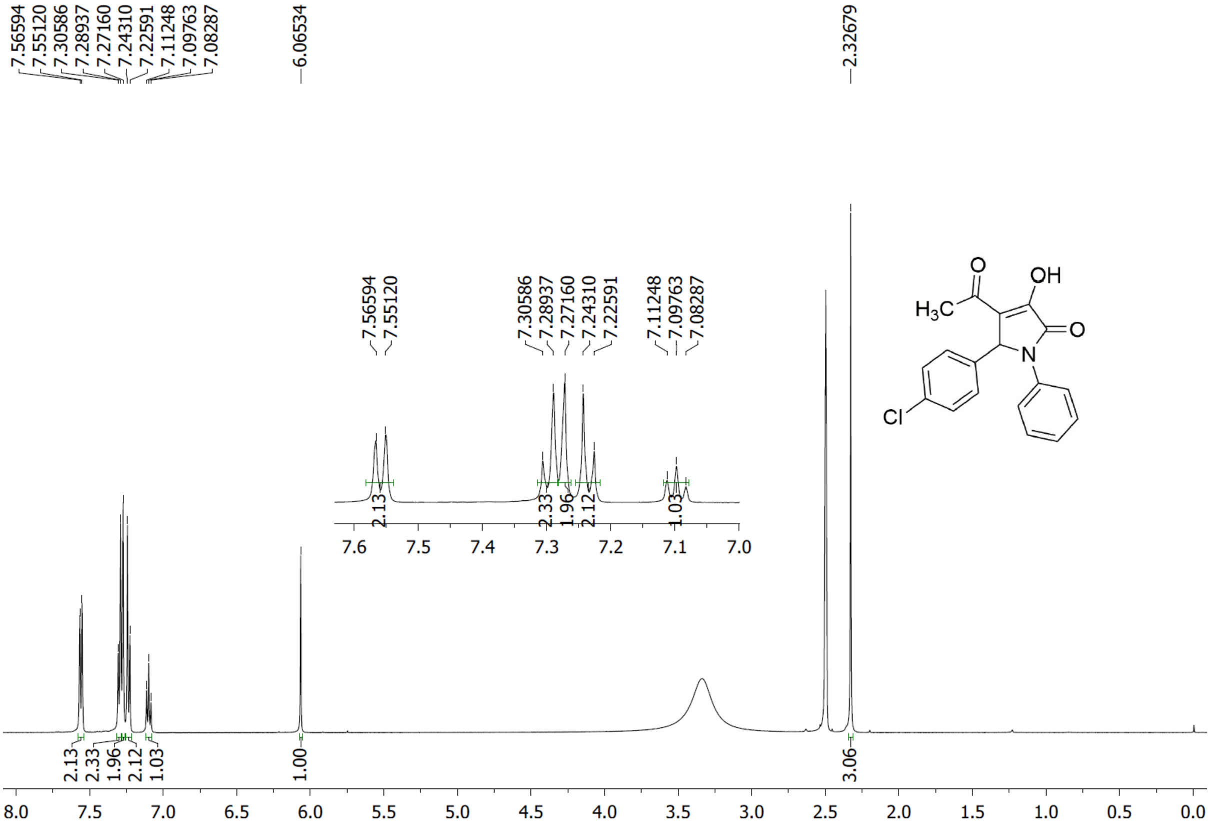

13C NMR spectrum of 4c in DMSO-d6

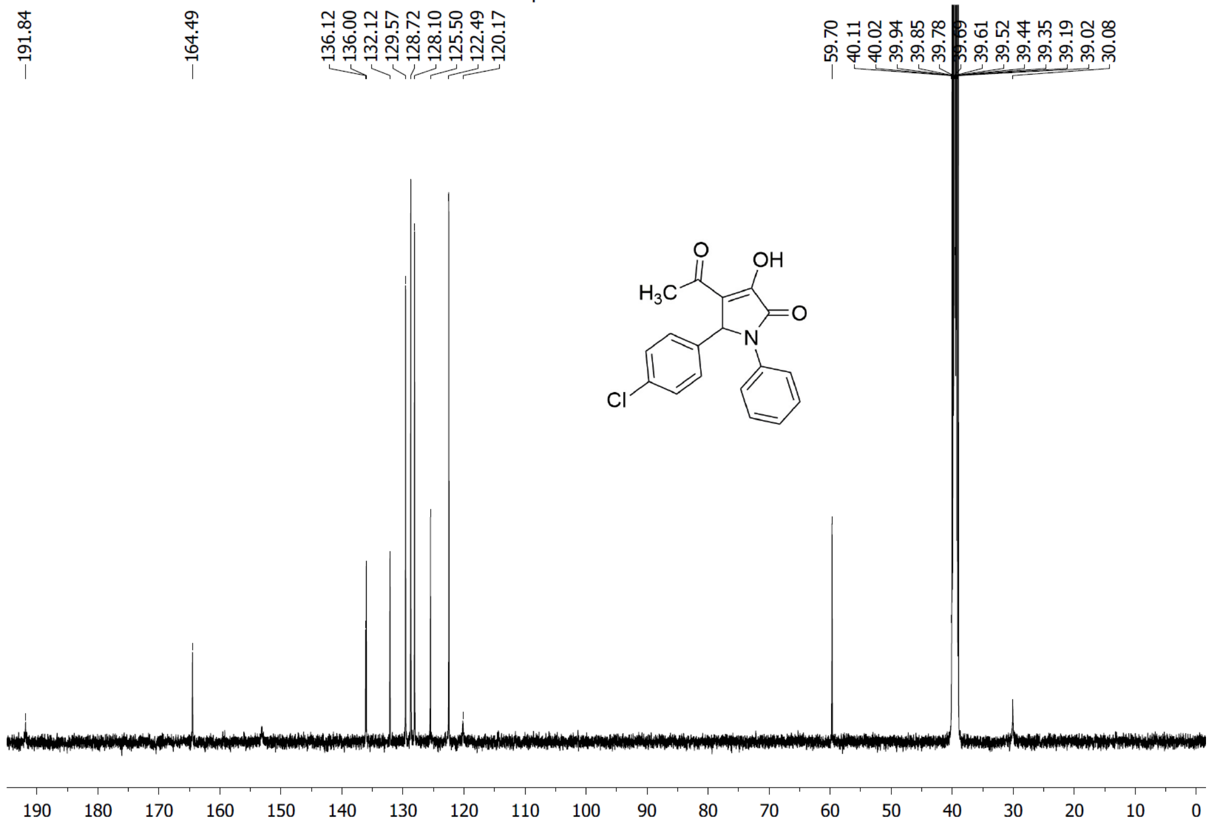

1H NMR spectrum of 4c in CDCl3, 500 MHz

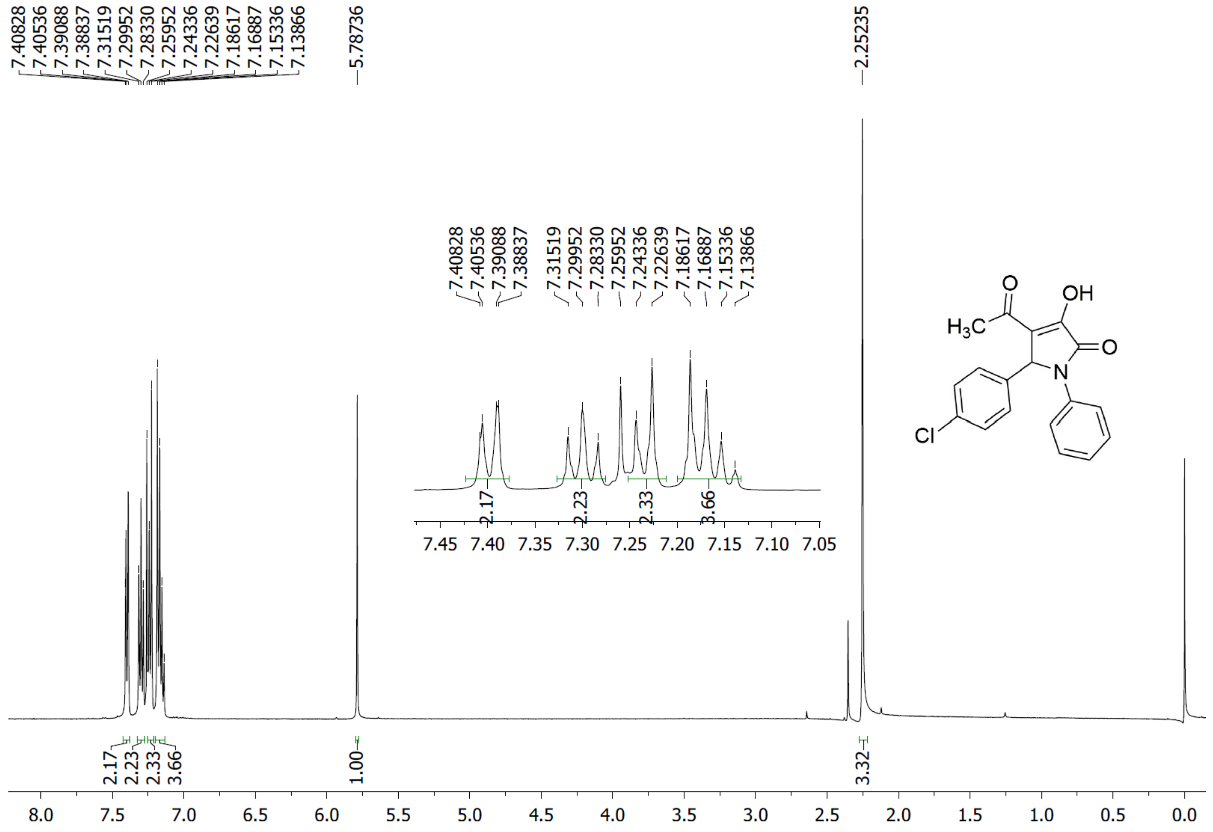

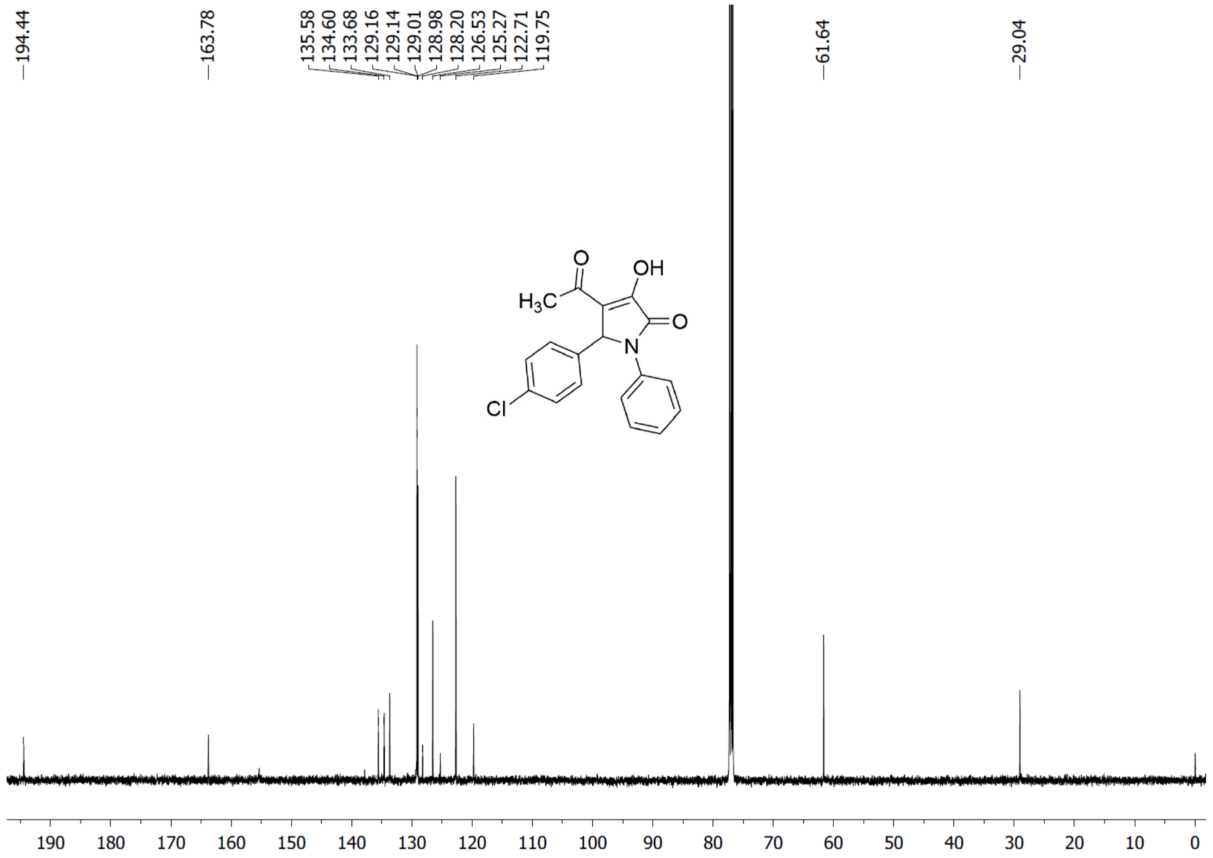

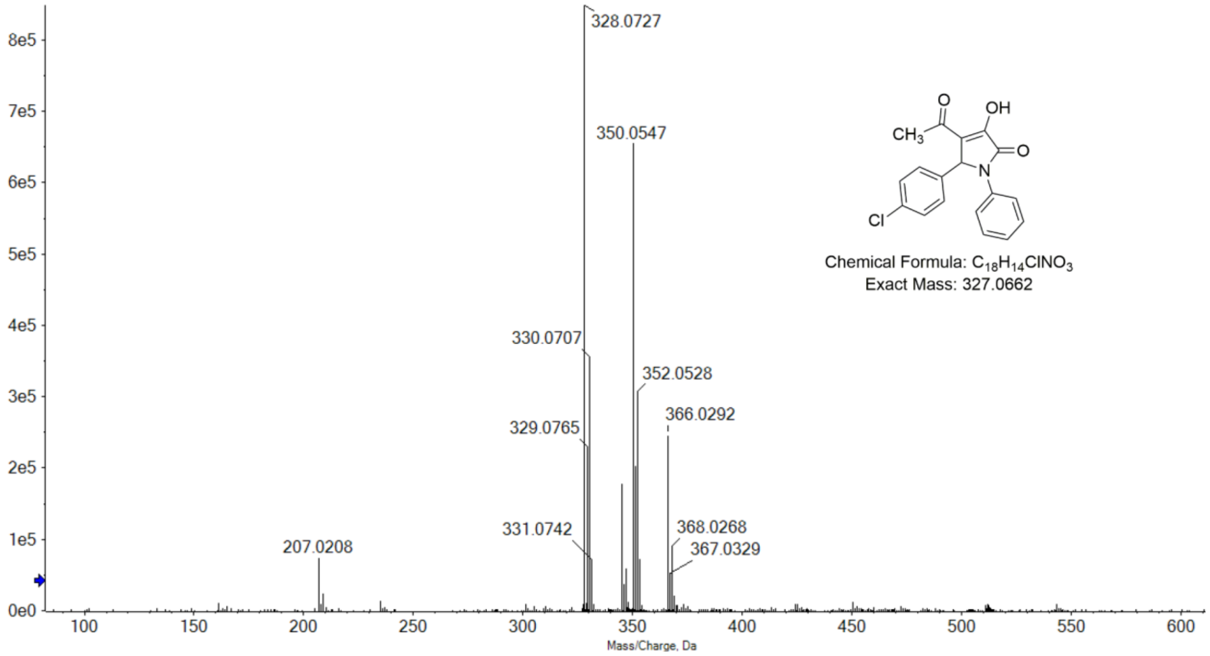

1H NMR spectrum of 4aa in CDCl3

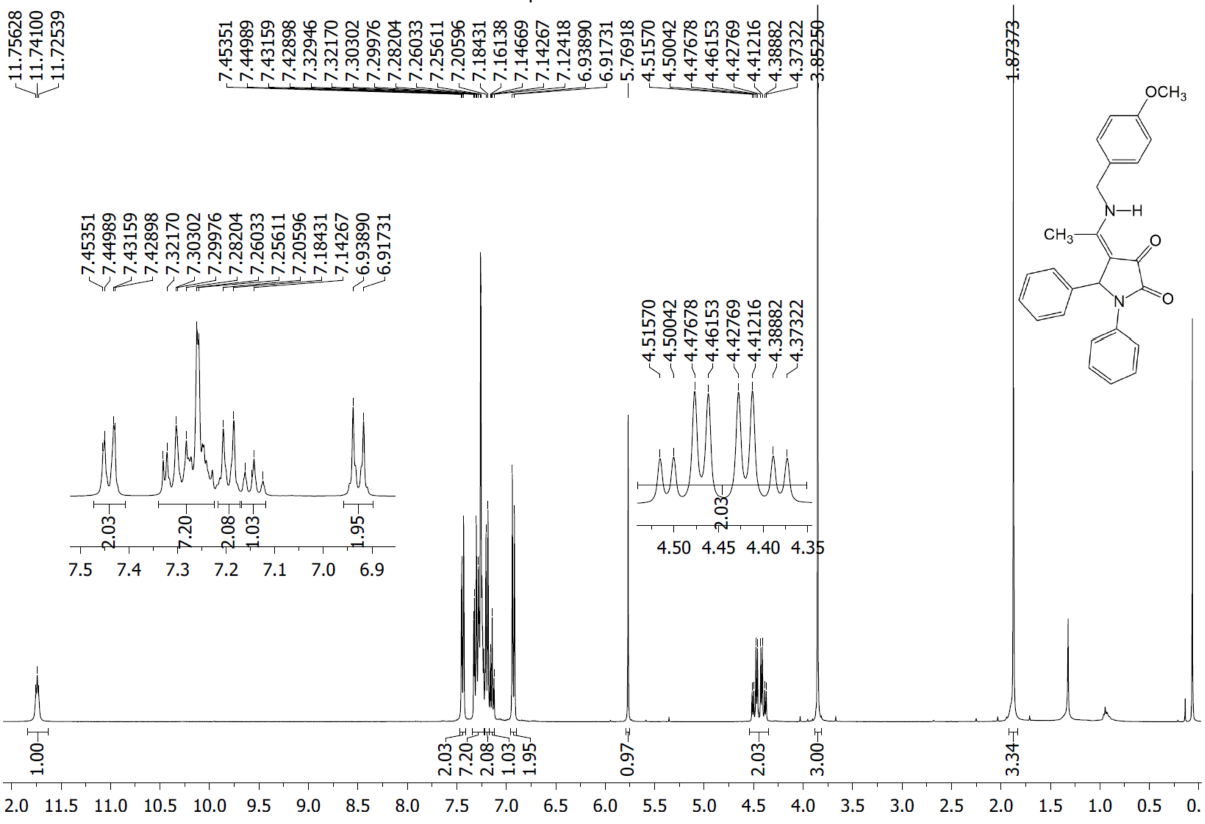

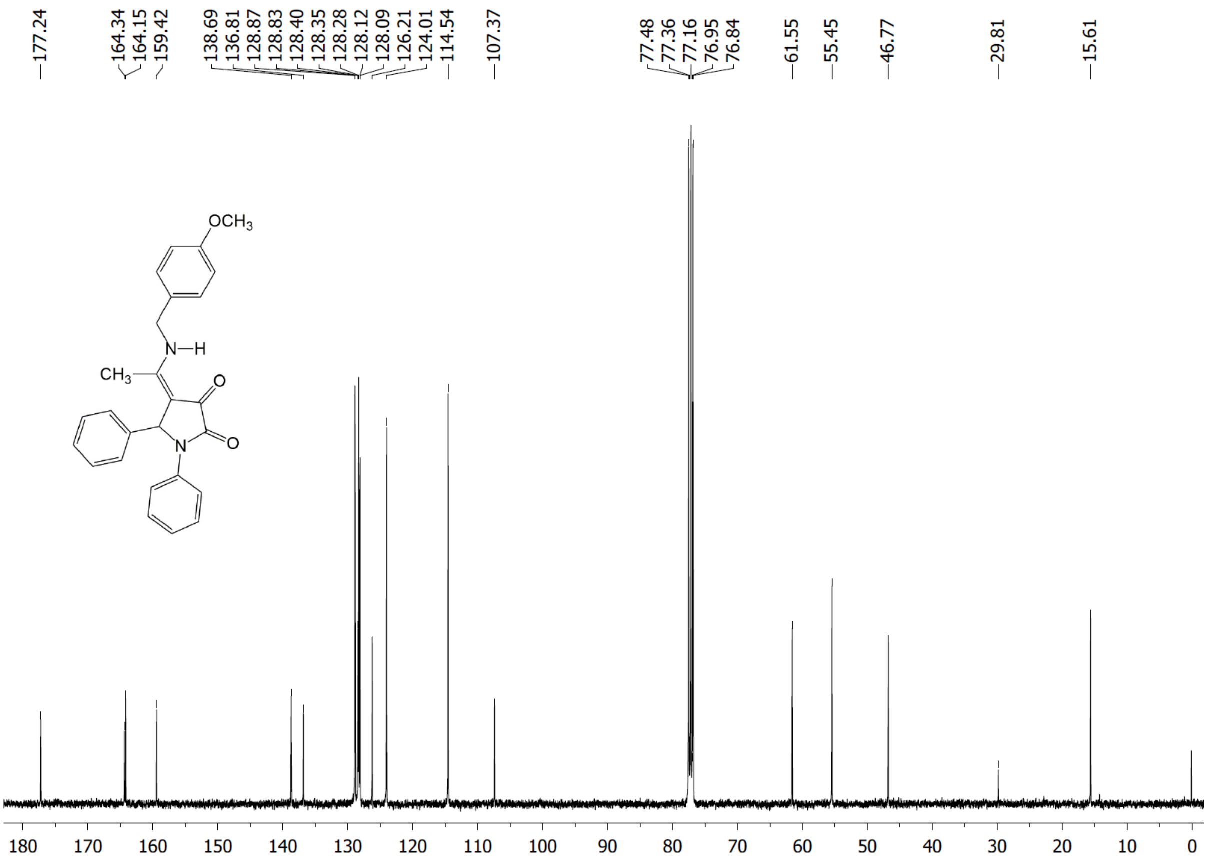

HSQC 2D NMR spectrum of 4aa

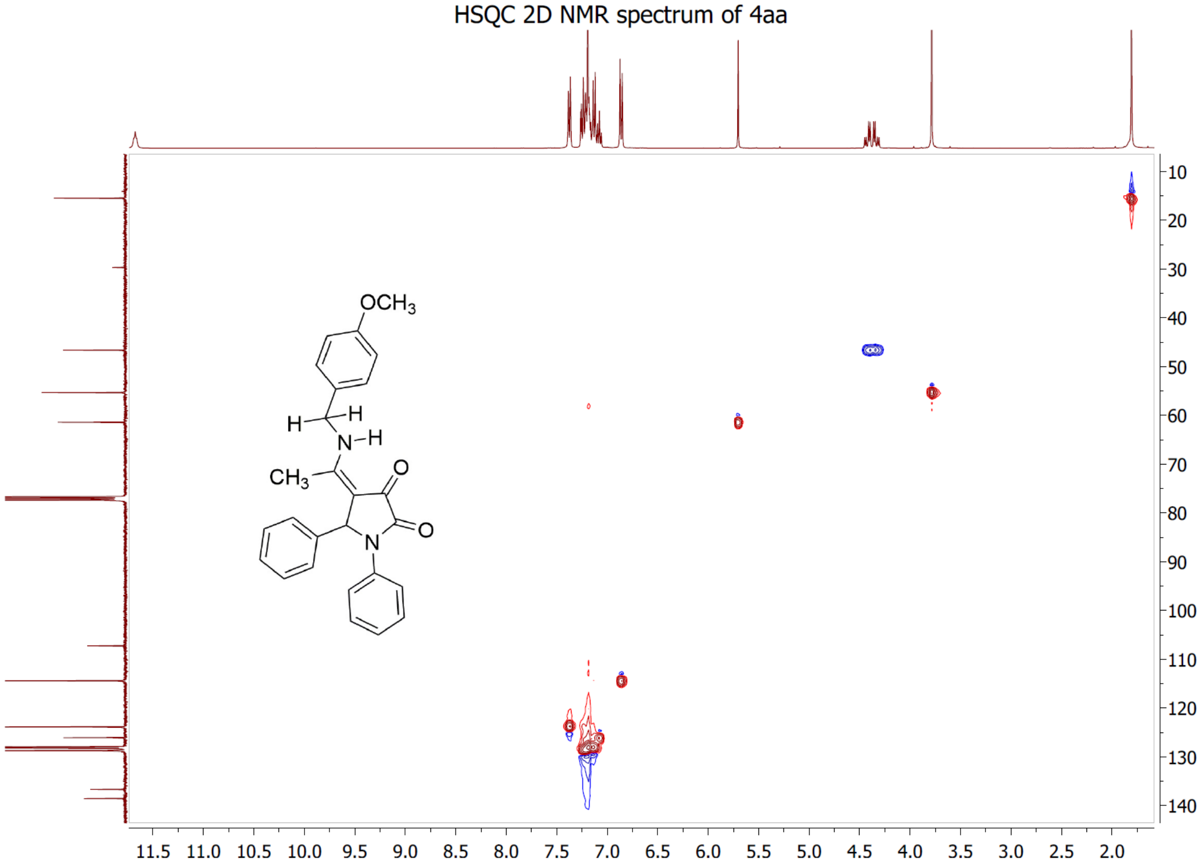

HMBC 2D NMR spectrum of 4aa

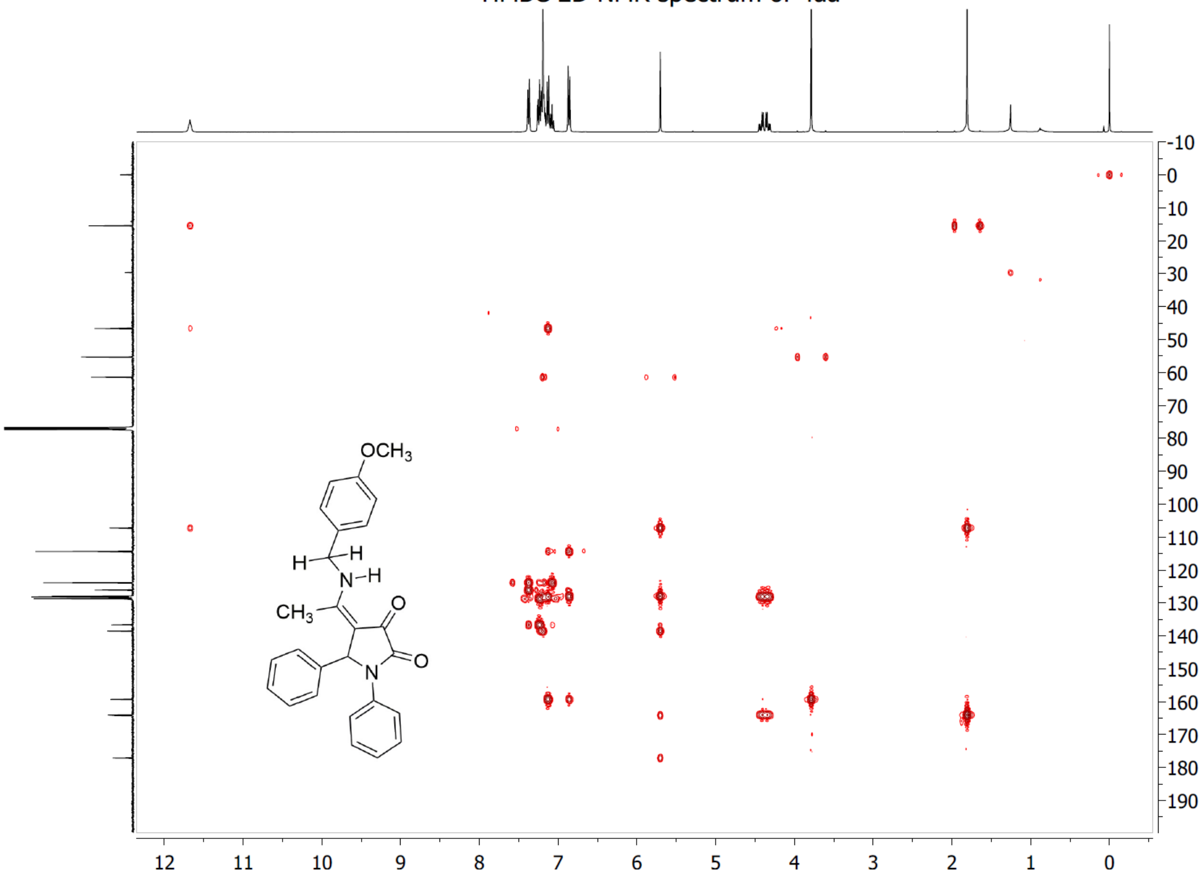

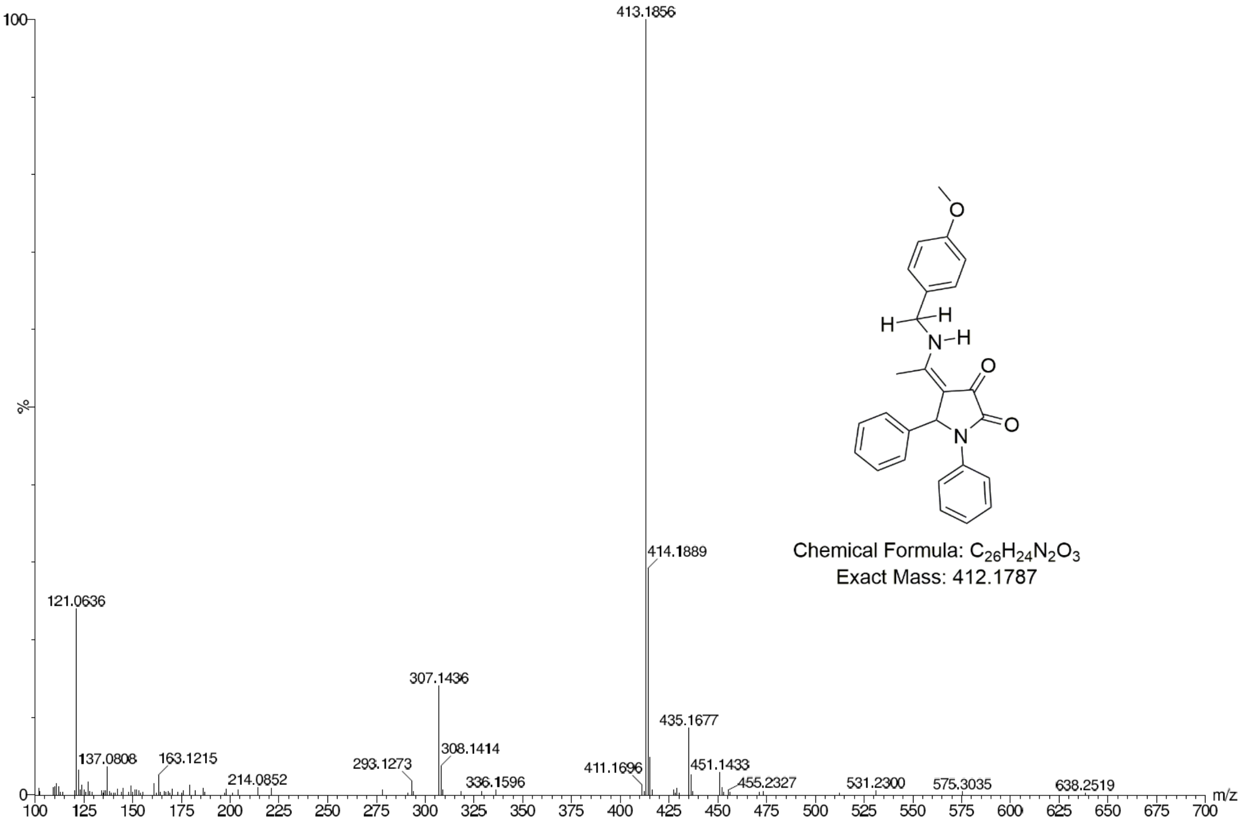

1H NMR spectrum of 4ab in CDCl3

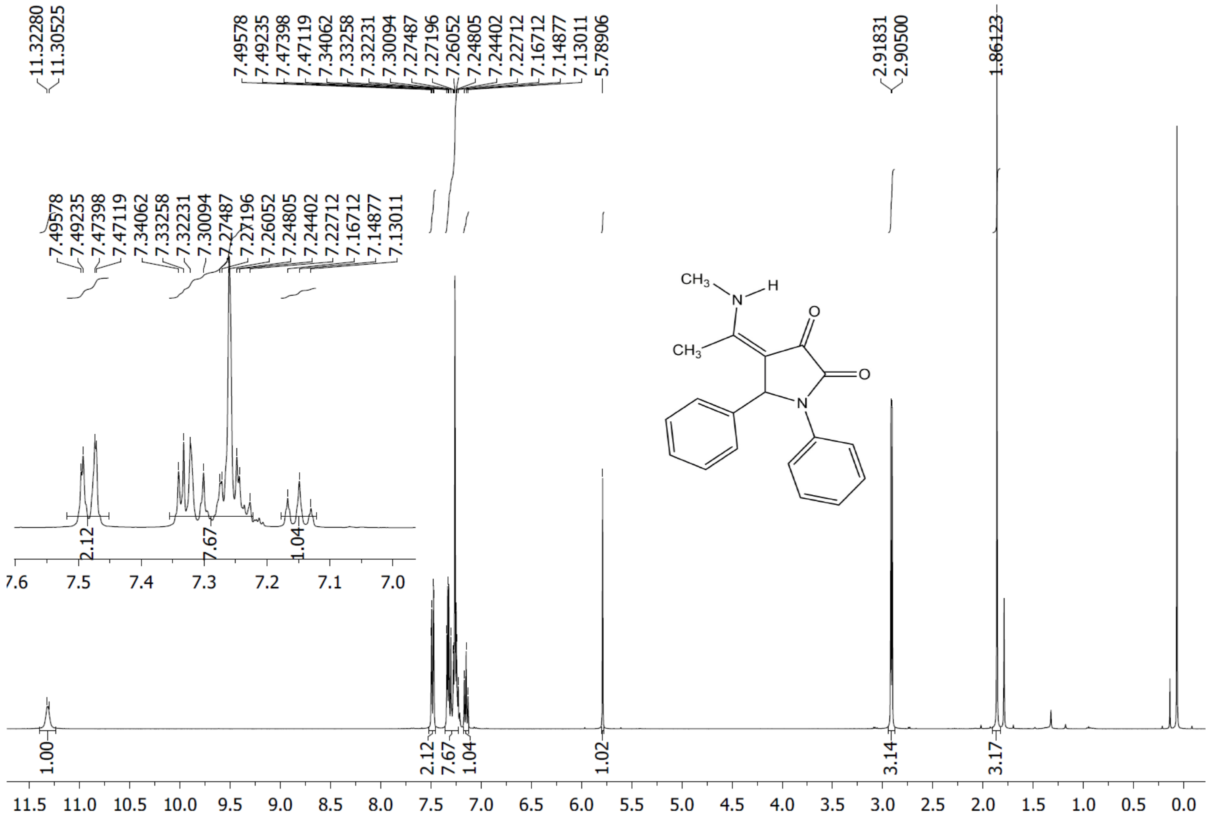

13C NMR spectrum of 4ab in CDCl3

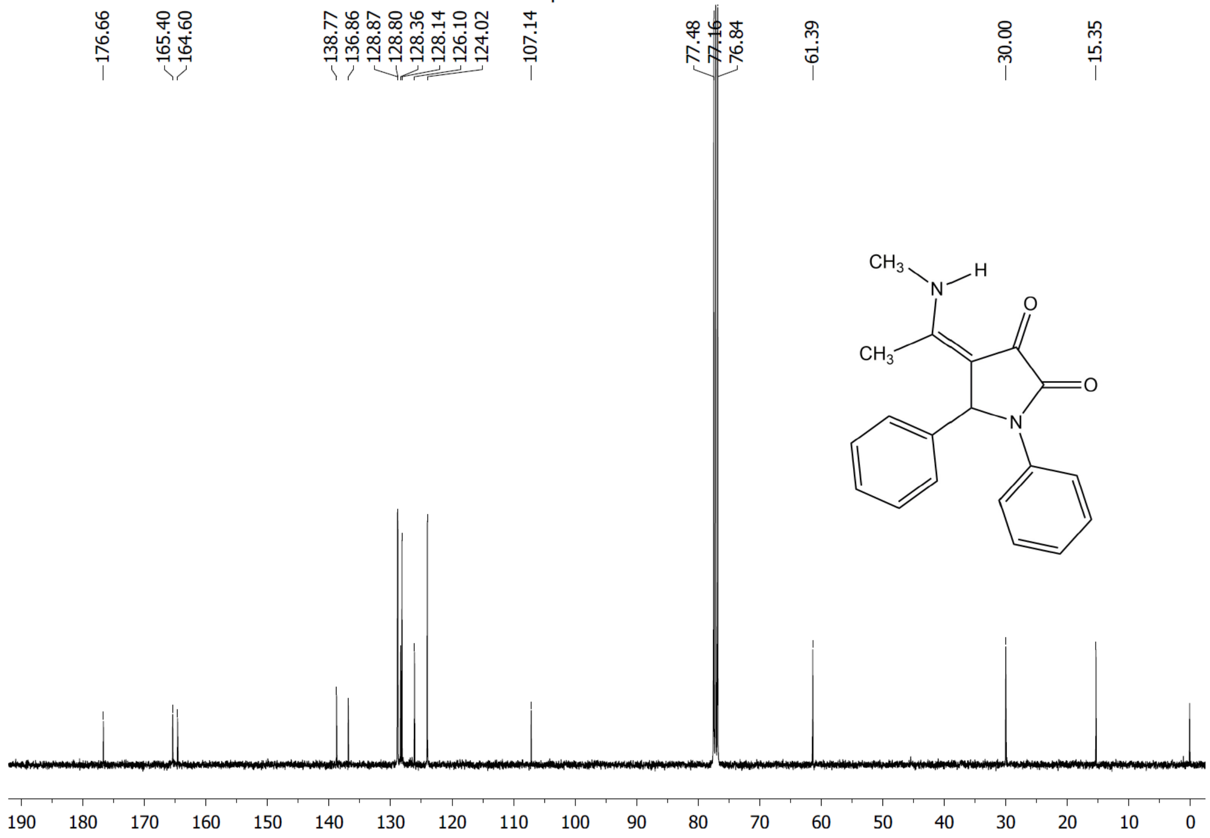

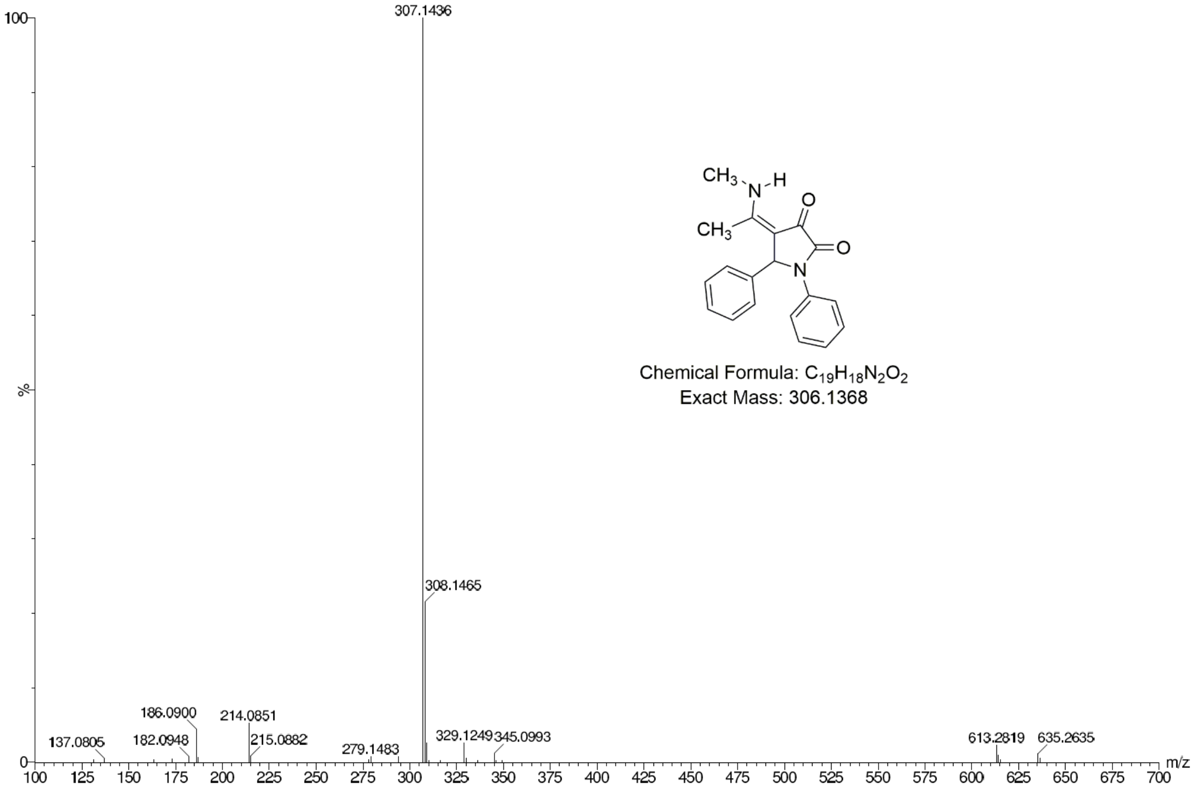

1H NMR spectrum of 4ac in CDCl3, 500 MHz

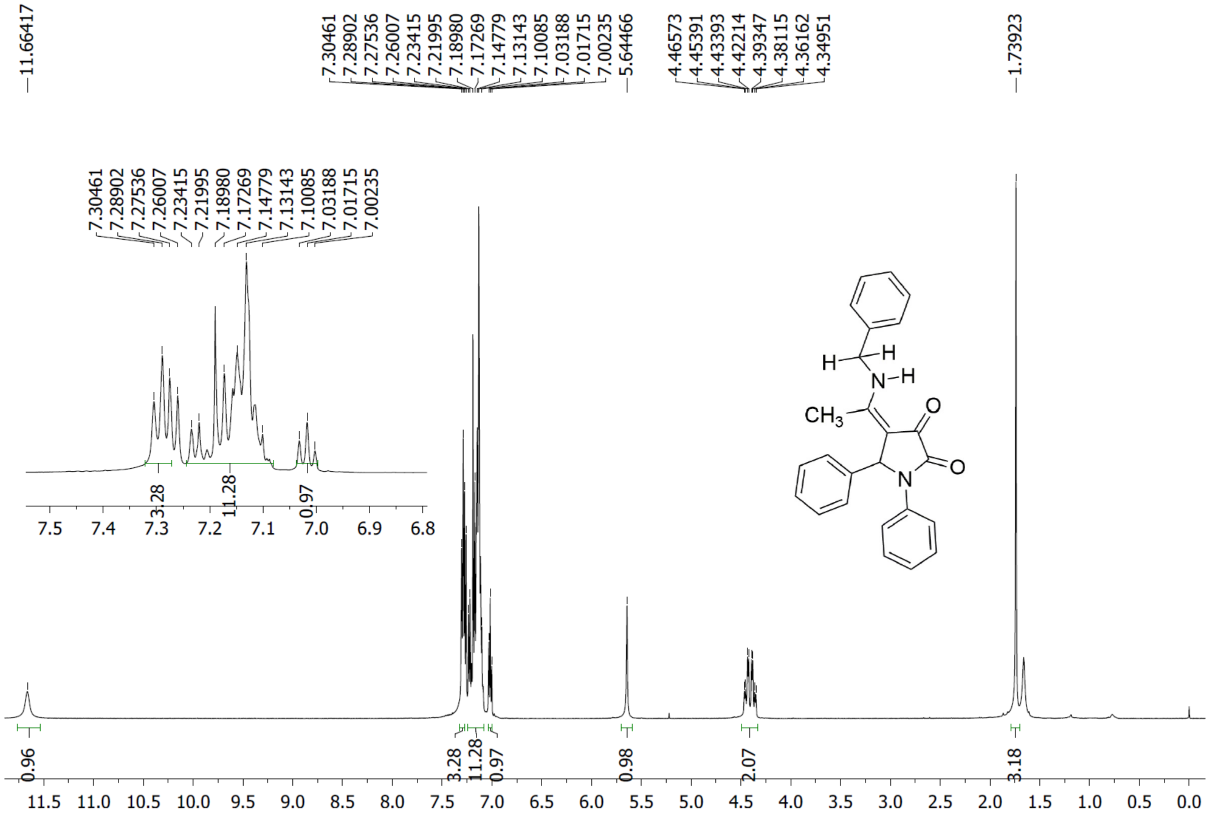

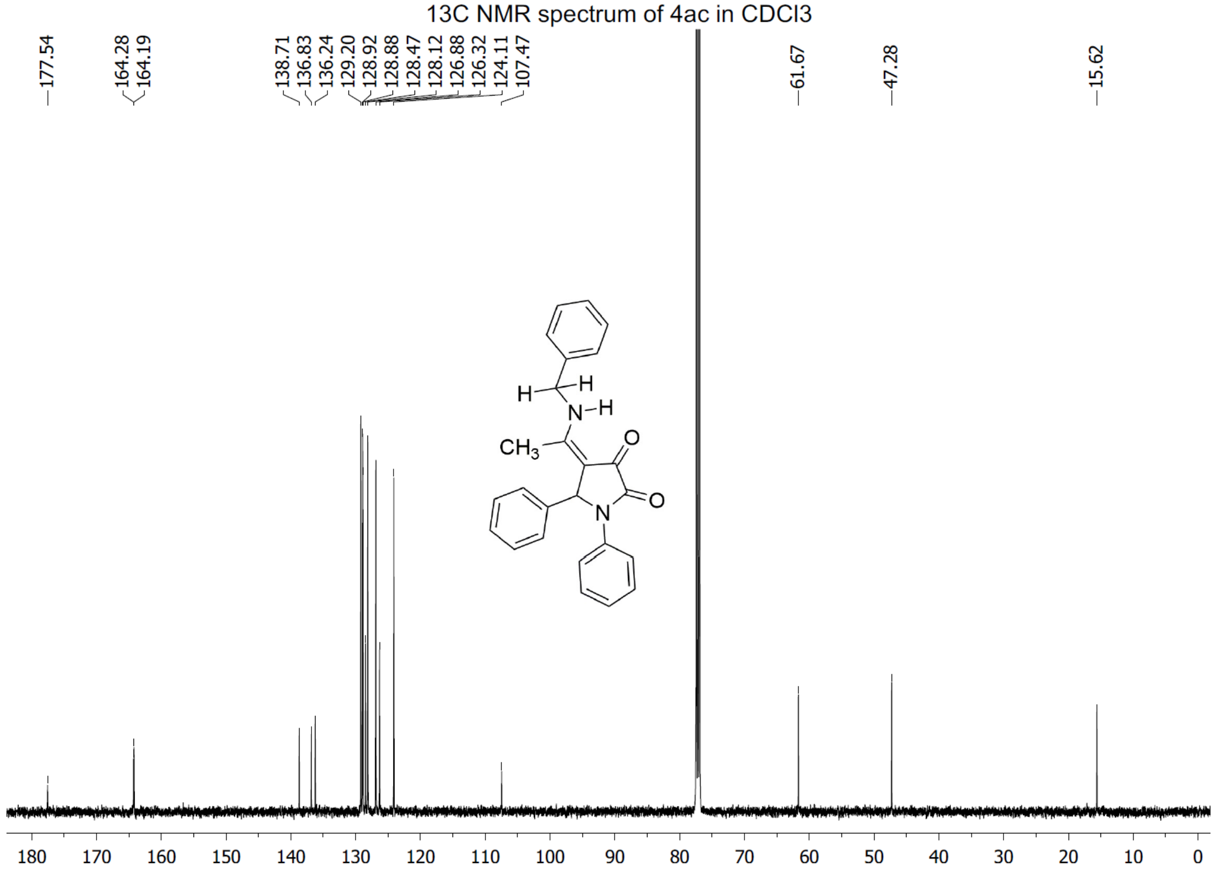
13C NMR spectrum of 4ac in CDCl3
177.54
164.28
164.19
138.71
136.83
136.24
129.20
128.92
128.88
128.47
128.12
126.88
126.32
124.11
107.47
61.67
47.28
15.62
H
H
N
H
CH3
O
O
N
180 170 160 150 140 130 120 110 100 90 80 70 60 50 40 30 20 10 0

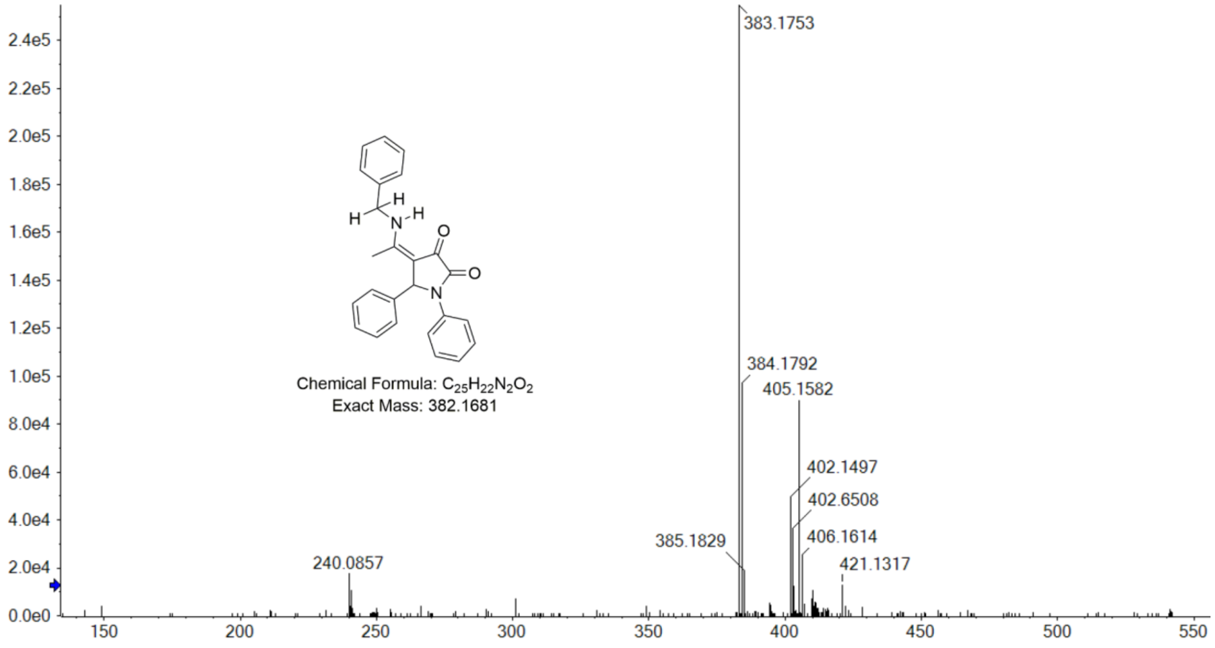
383.1753
384.1792
405.1582
402.1497
402.6508
406.1614
421.1317
385.1829
240.0857
Chemical Formula: C25H22N2O2
Exact Mass: 382.1681
2.4e5 2.2e5 2.0e5 1.8e5 1.6e5 1.4e5 1.2e5 1.0e5 8.0e4 6.0e4 4.0e4 2.0e4 0.0e0
150 200 250 300 350 400 450 500 550

1H NMR spectrum of 4ad in CDCl3

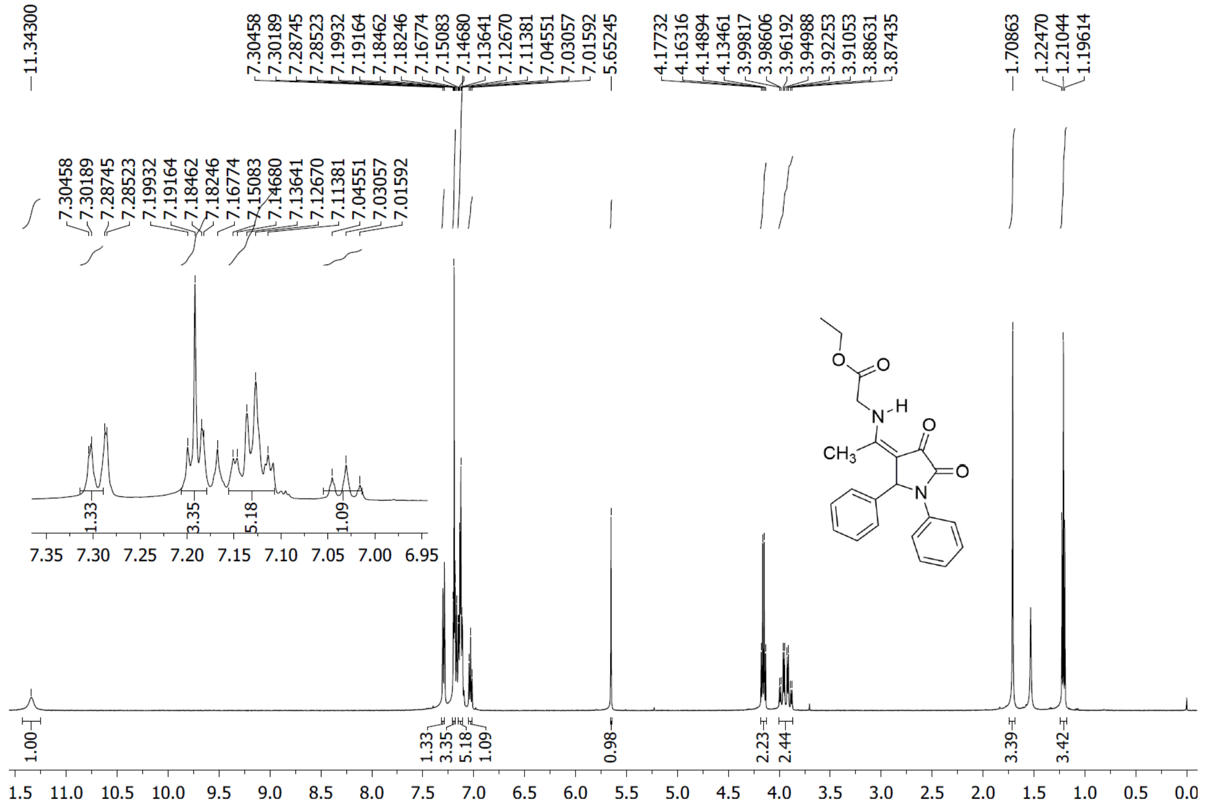

13C NMR spectrum of 4ad in CDCl3

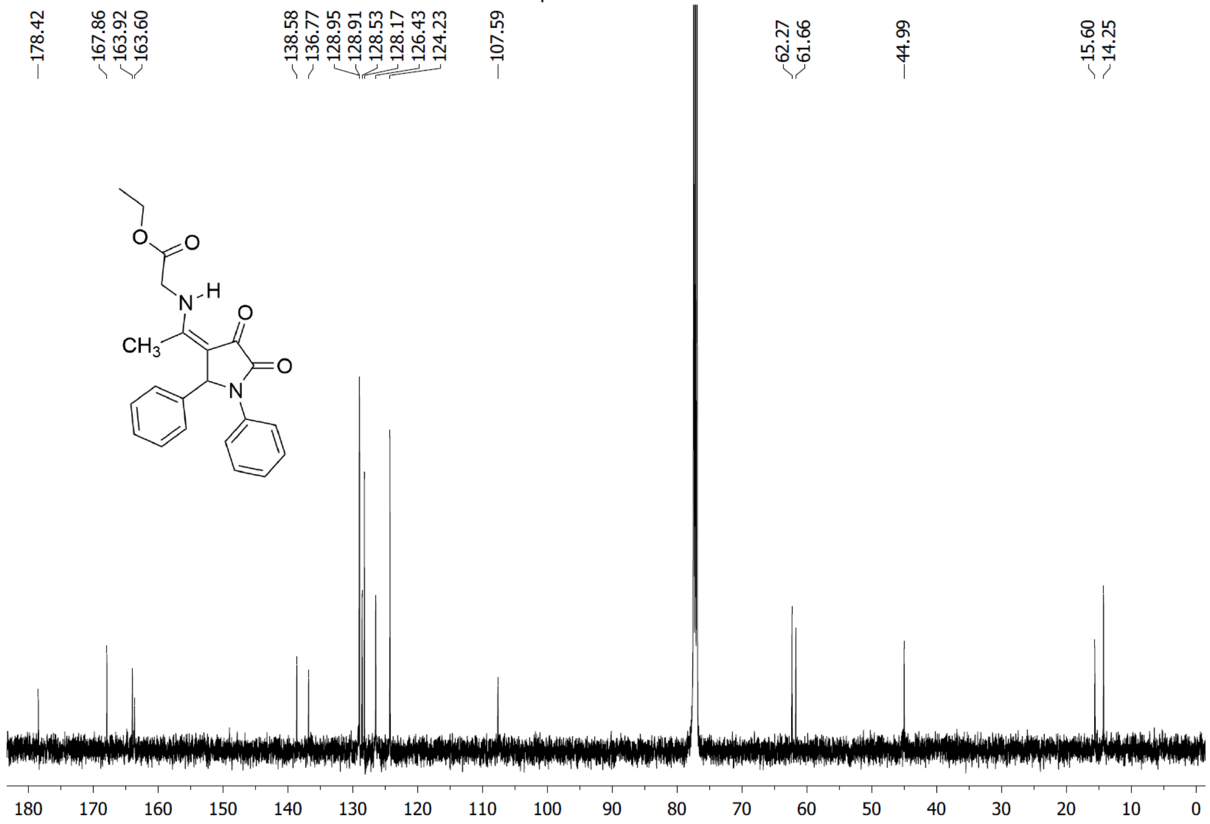

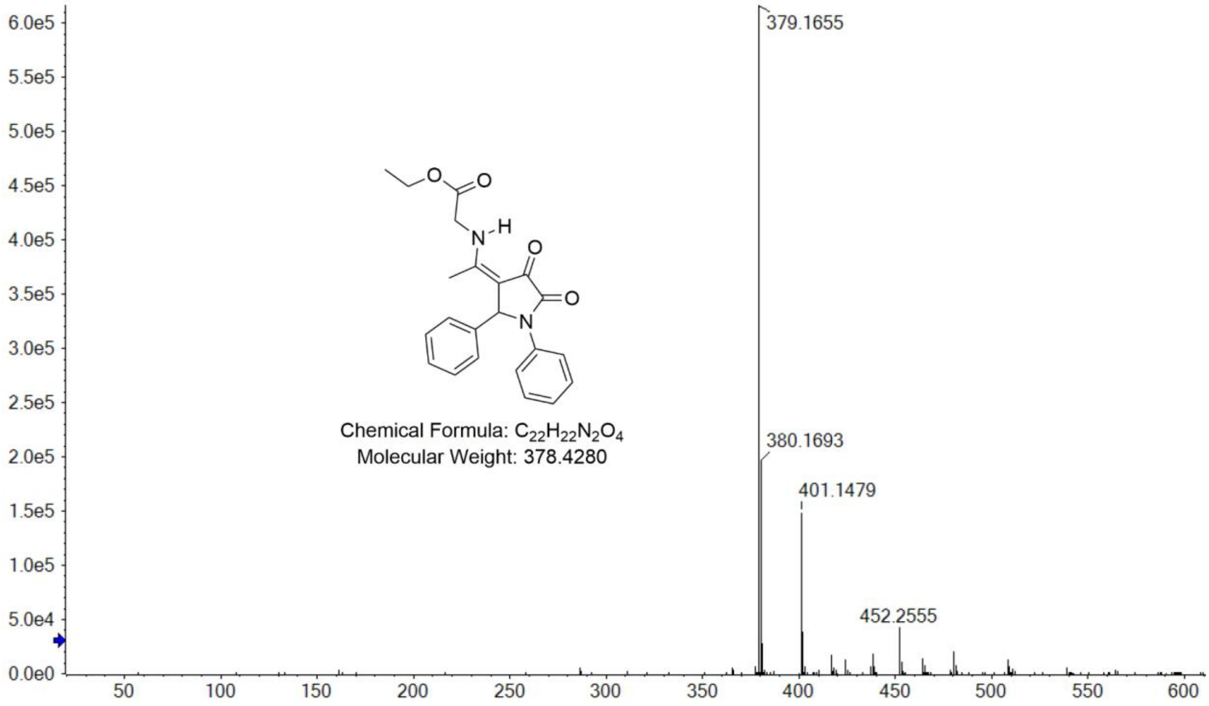

1H NMR spectrum of 4ba in CDCl3, 600 MHz

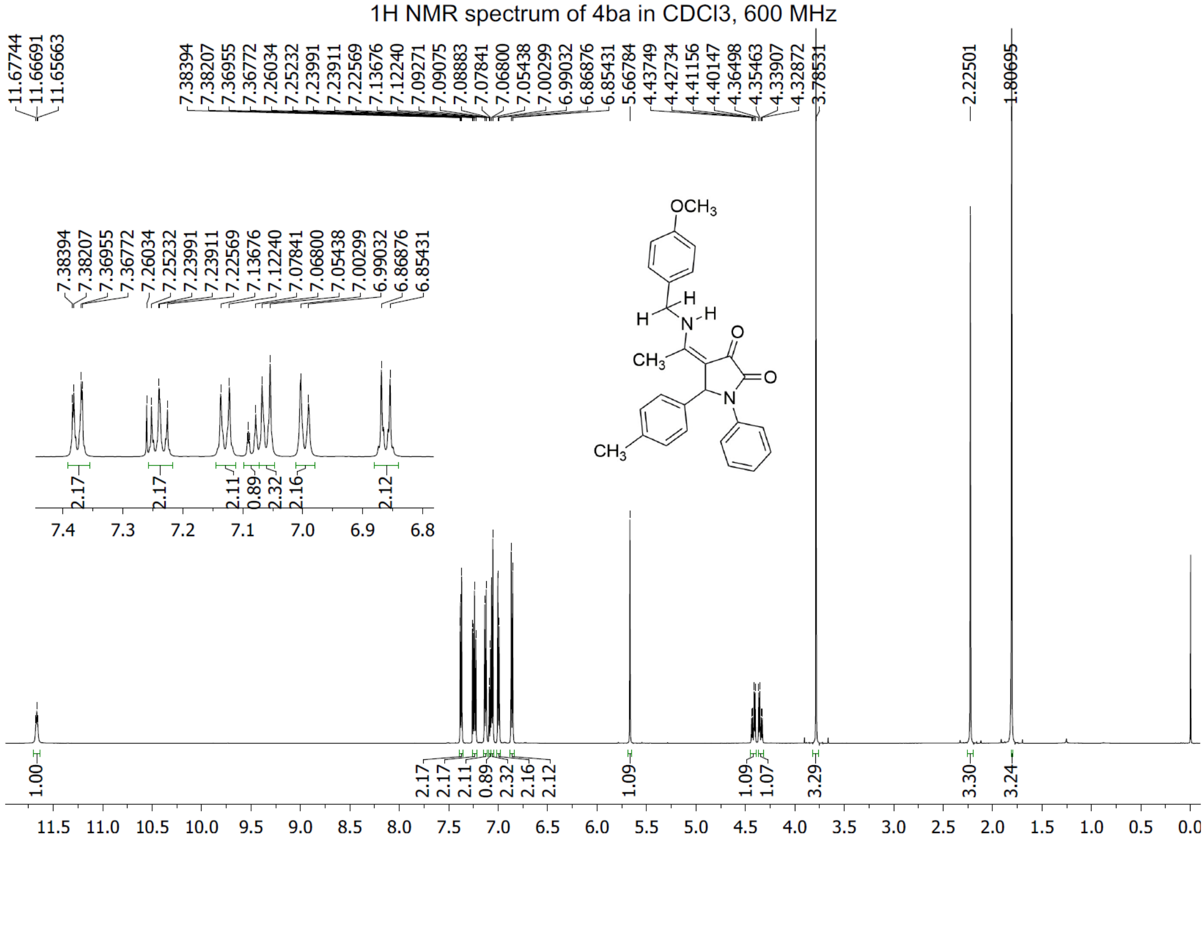

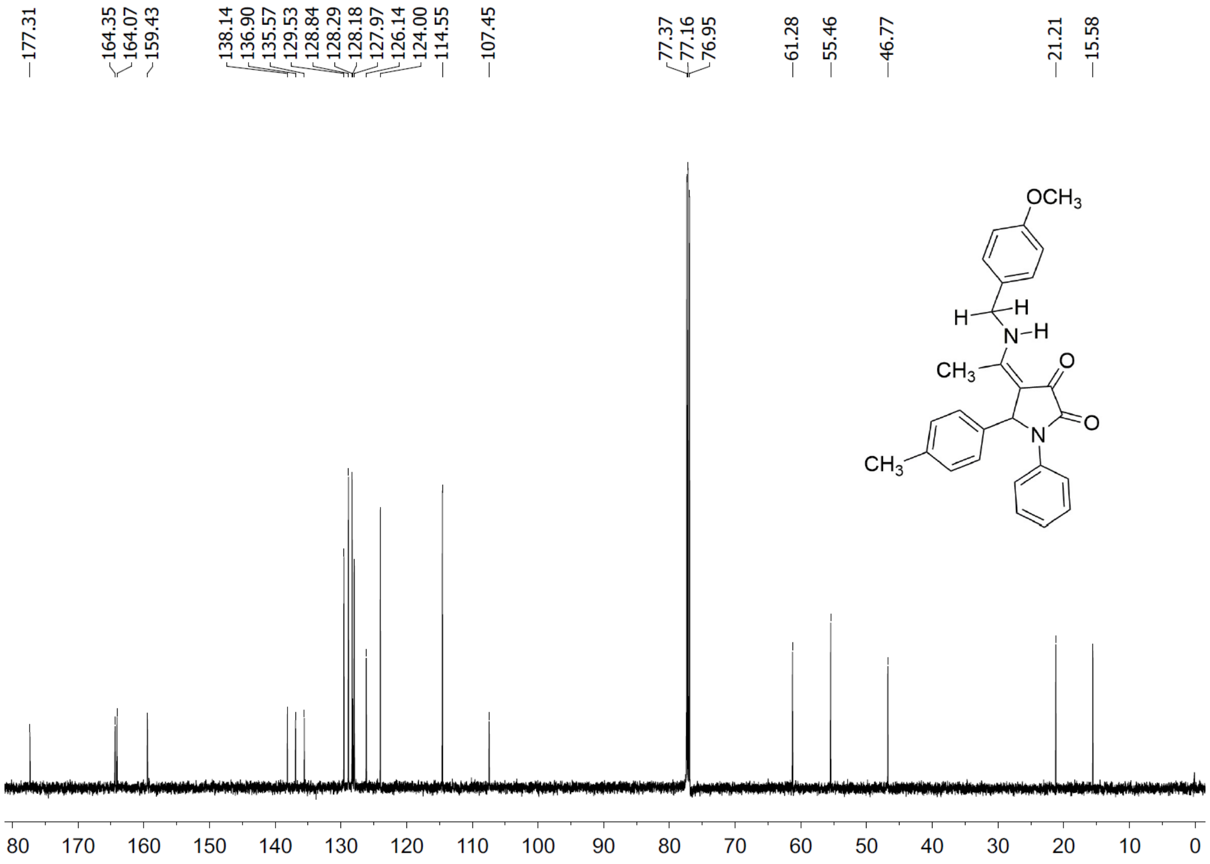
177.31
164.35
164.07
159.43
138.14
136.90
135.57
129.53
128.84
128.29
128.18
127.97
126.14
124.00
114.55
107.45
77.37
77.16
76.95
61.28
55.46
46.77
21.21
15.58
OCH3
H
H
N-H
CH3
O
O
N
CH3
80 170 160 150 140 130 120 110 100 90 80 70 60 50 40 30 20 10 0

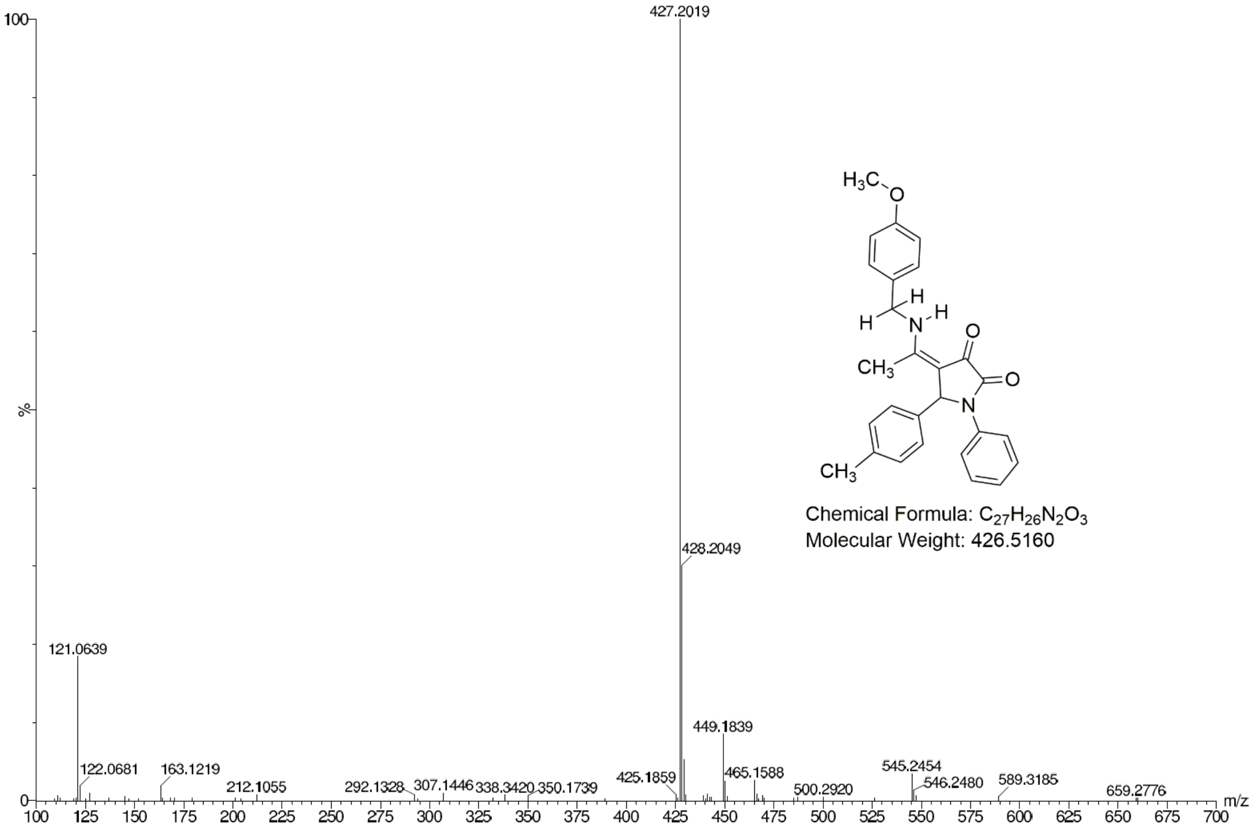
427.2019
100
%
0
428.2049
121.0639
449.1839
122.0681
163.1219
212.1055
292.1328
307.1446
338.3420
350.1739
425.1859
465.1588
500.2920
545.2454
546.2480
589.3185
659.2776
m/z
100 125 150 175 200 225 250 275 300 325 350 375 400 425 450 475 500 525 550 575 600 625 650 675 700
H3C
O
H
H
N
H
O
CH3
O
N
CH3
Chemical Formula: C27H26N2O3
Molecular Weight: 426.5160

1H NMR spectrum of 4bb in CDCl3

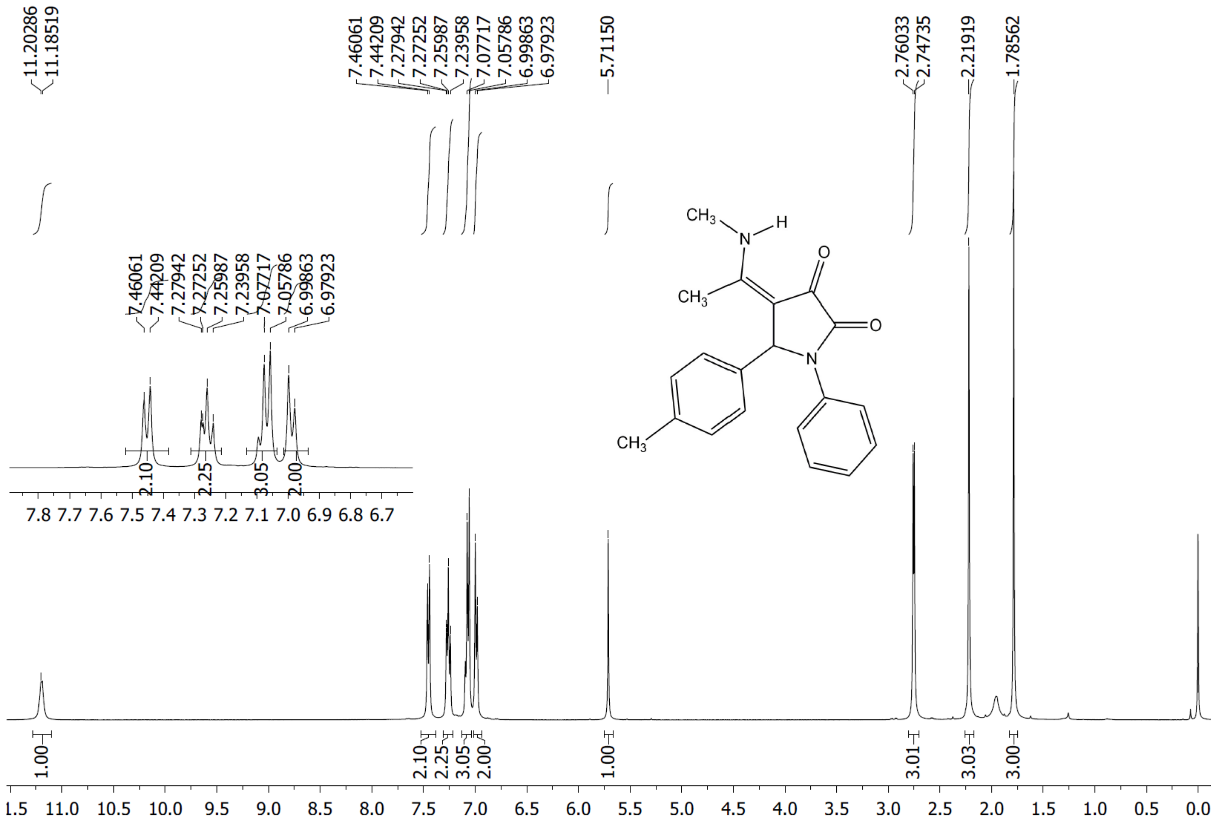

13 C NMR spectrum of 4bb in CDCl3

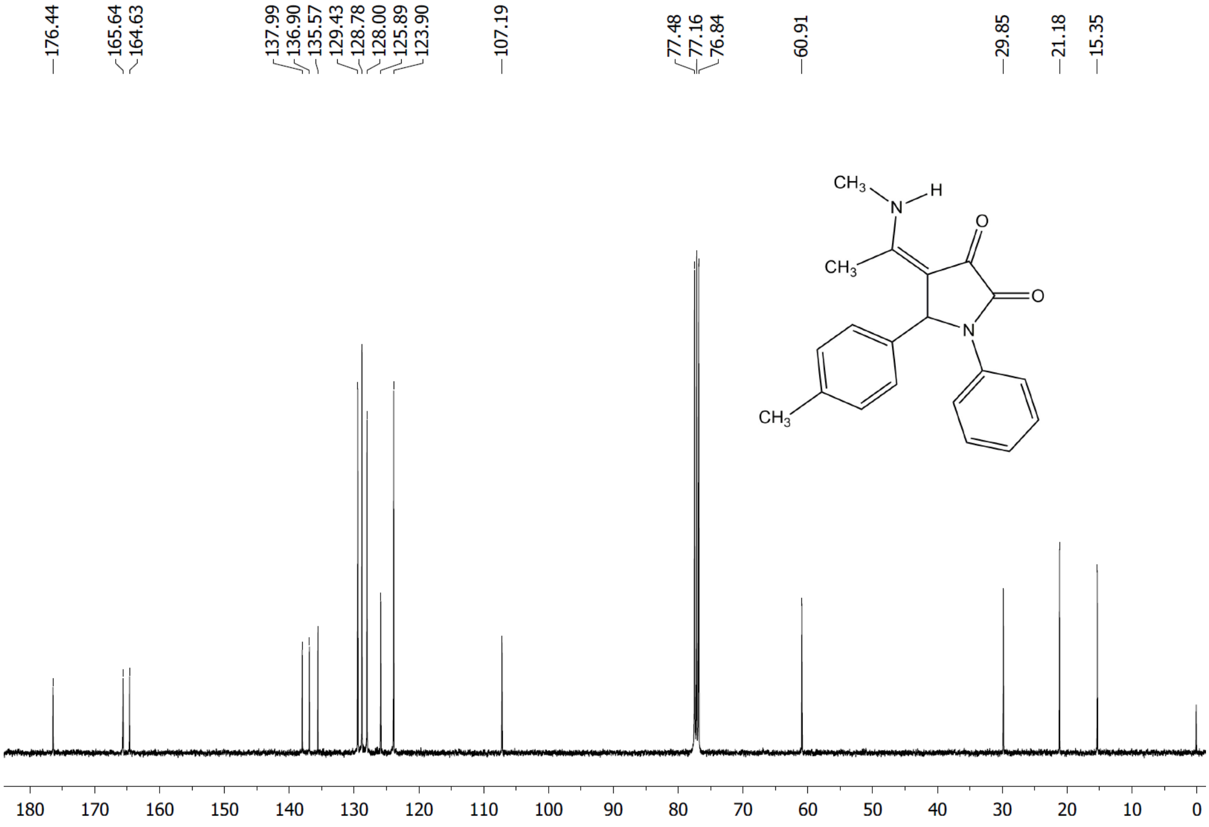

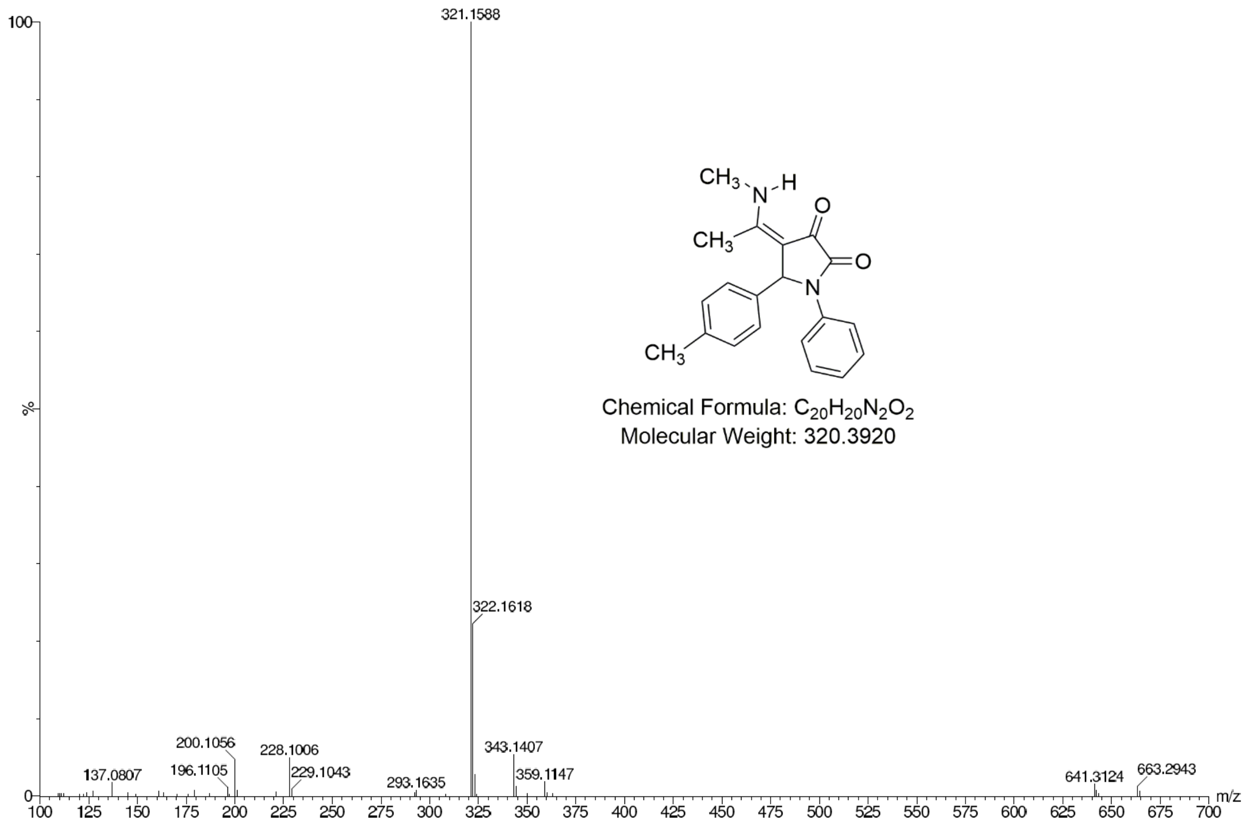

H NMR spectrum of 4bc in CDCl3, 500 MHz

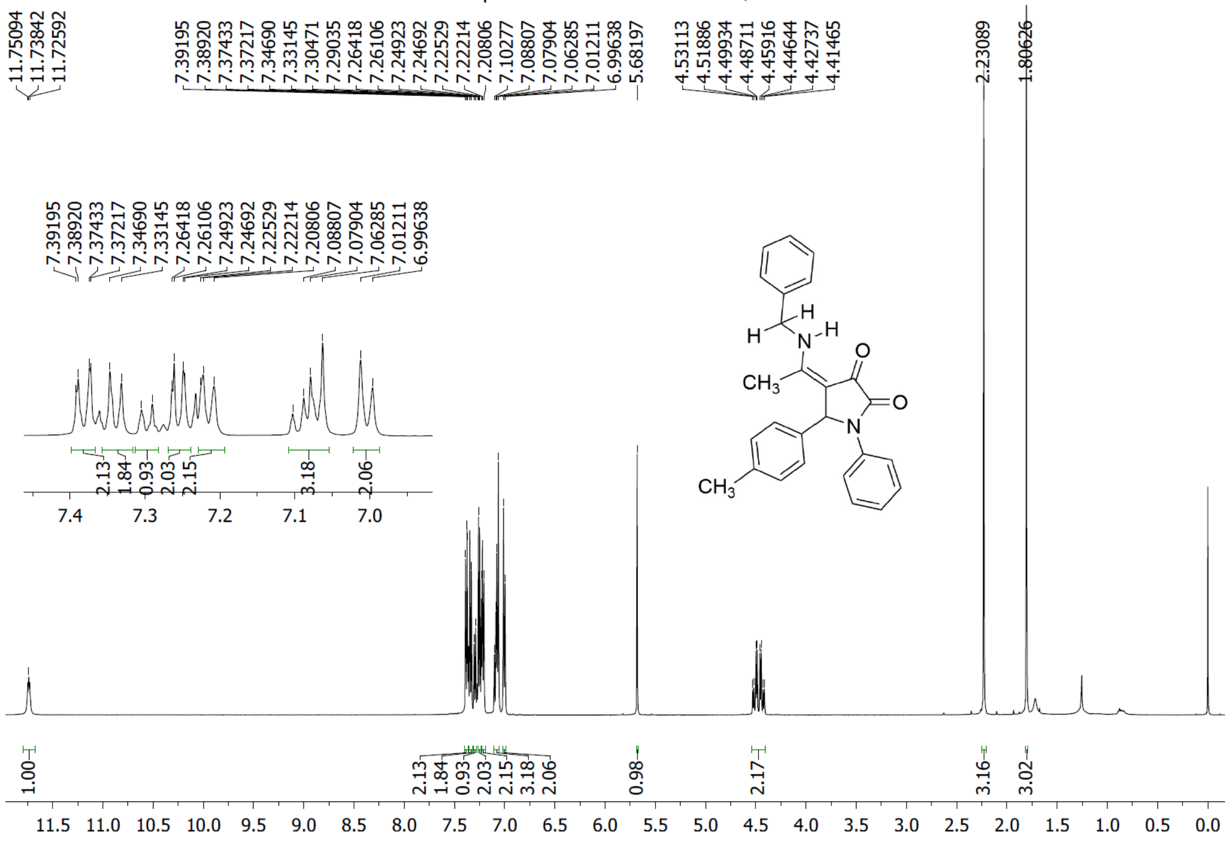

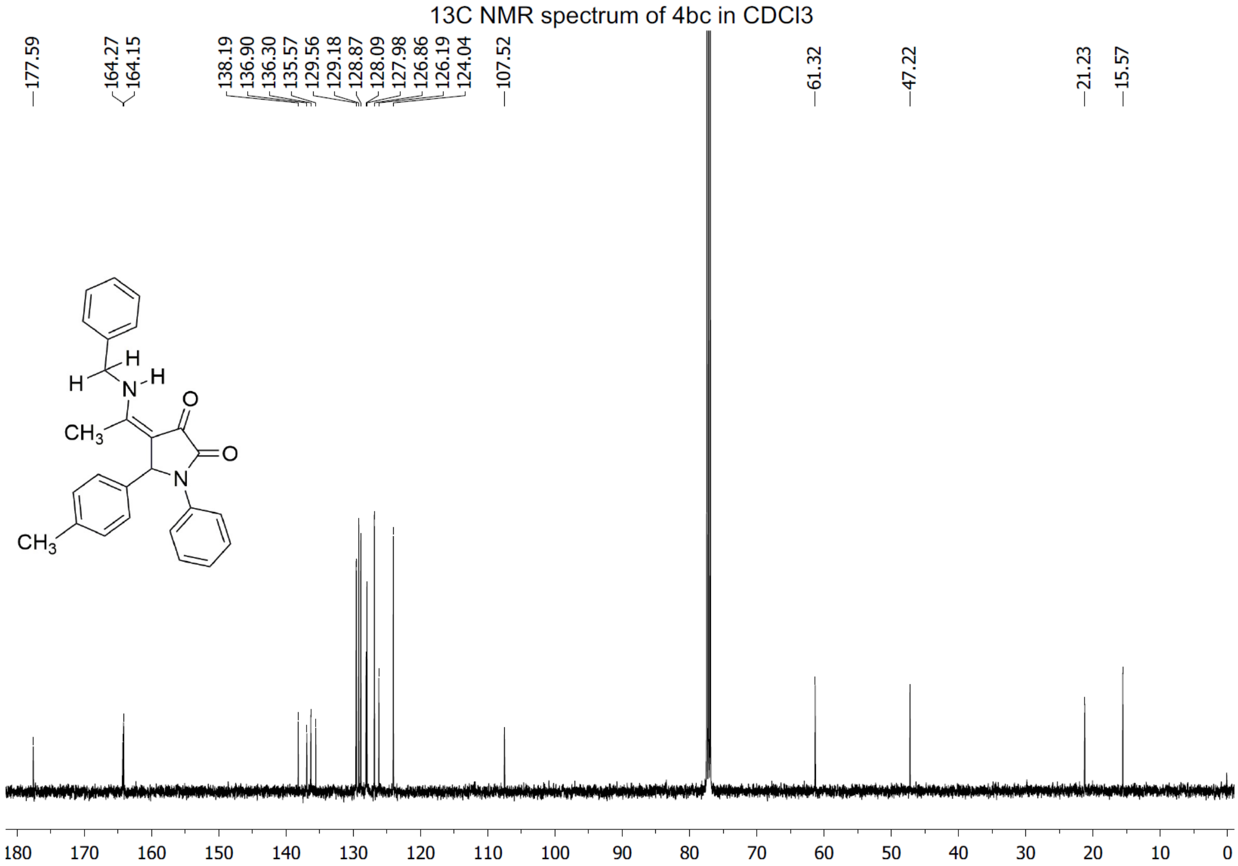
13C NMR spectrum of 4bc in CDCl3
177.59
164.27
164.15
138.19
136.90
136.30
135.57
129.56
129.18
128.87
128.09
127.98
126.86
126.19
124.04
107.52
61.32
47.22
21.23
15.57
180 170 160 150 140 130 120 110 100 90 80 70 60 50 40 30 20 10 0

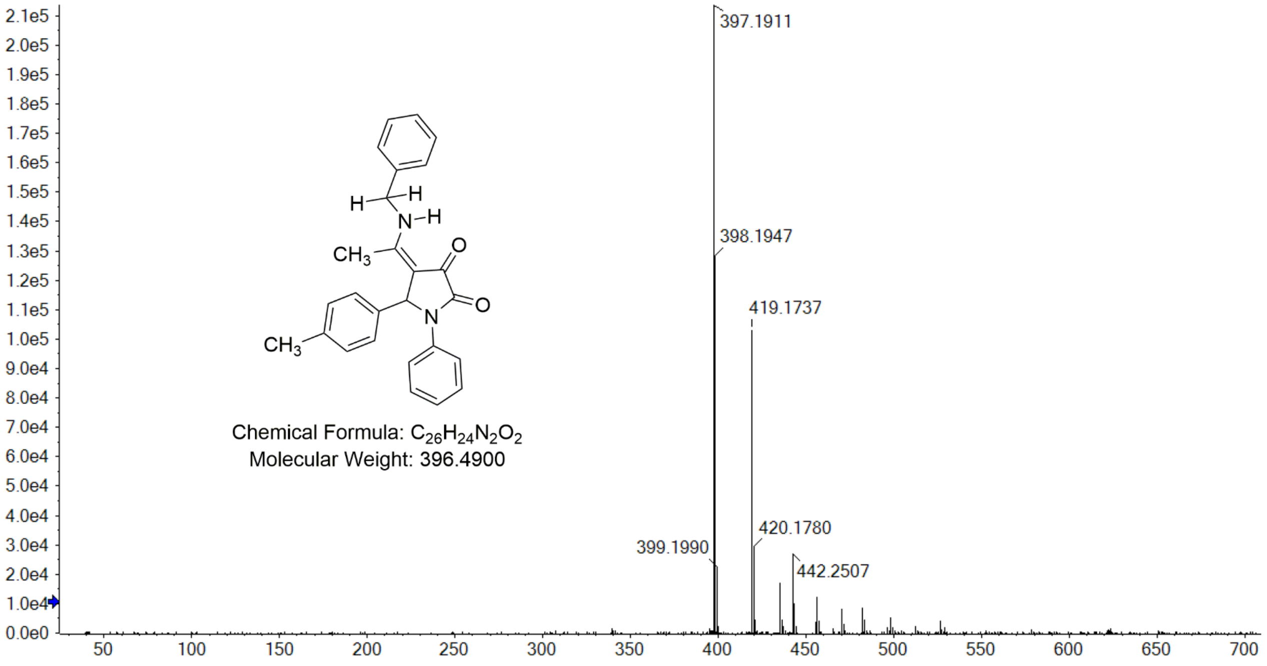
397.1911
398.1947
419.1737
420.1780
399.1990
442.2507
Chemical Formula: C26H24N2O2
Molecular Weight: 396.4900

1H NMR spectrum of 4bd in CDCl3

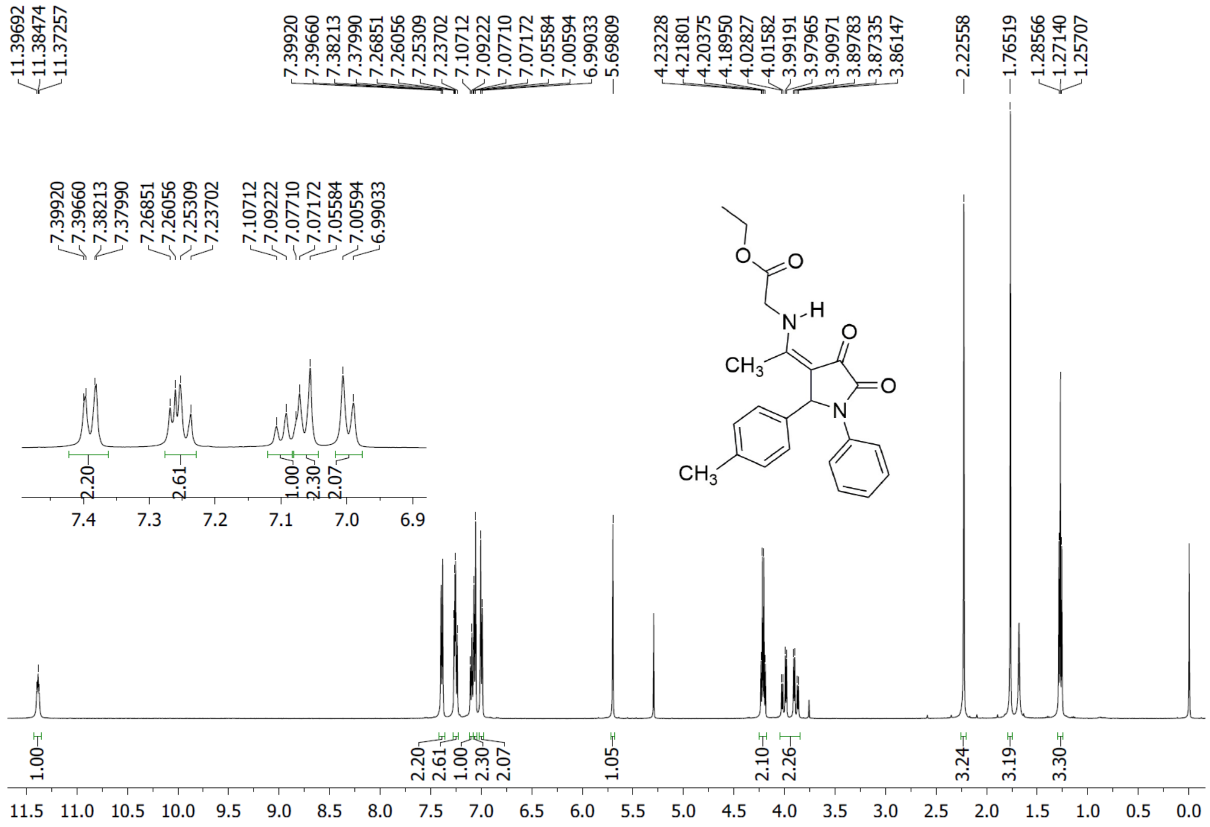

13C NMR spectrum of 4bd in CDCl3

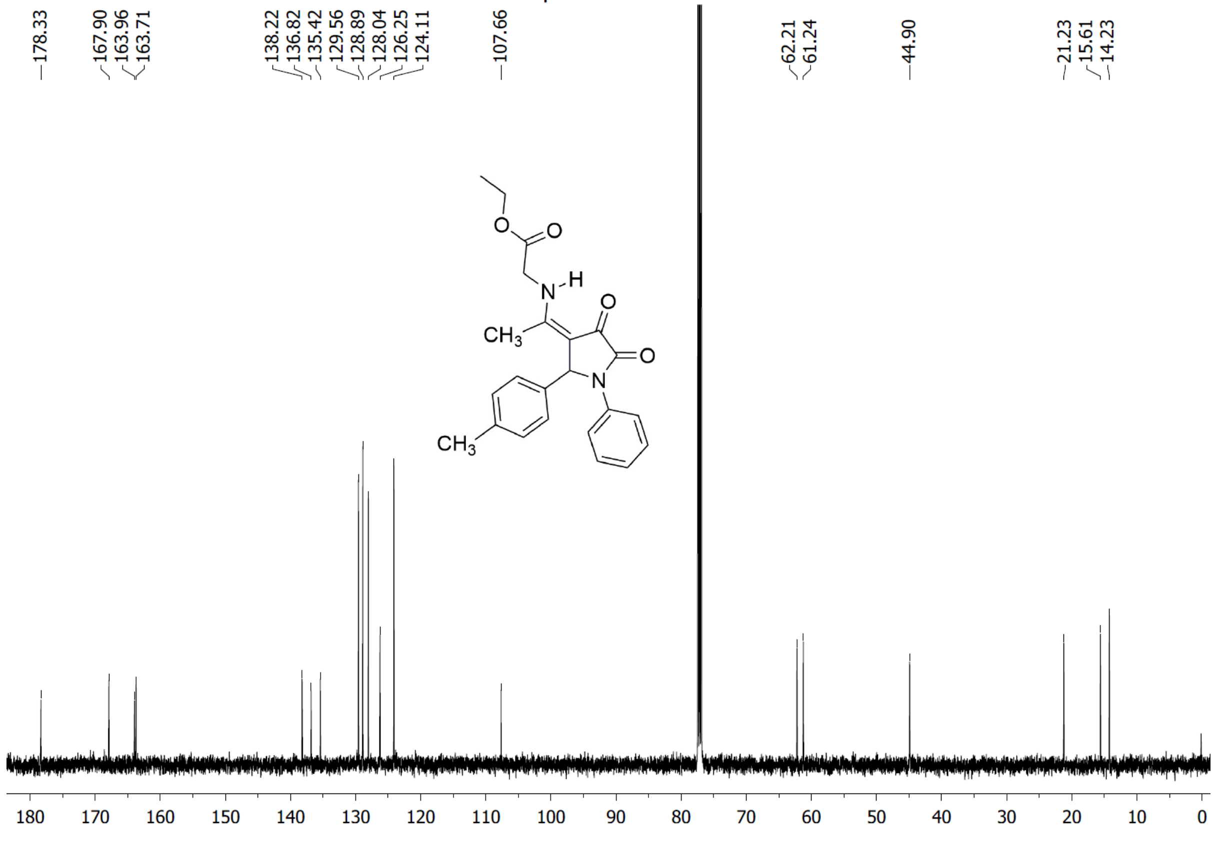

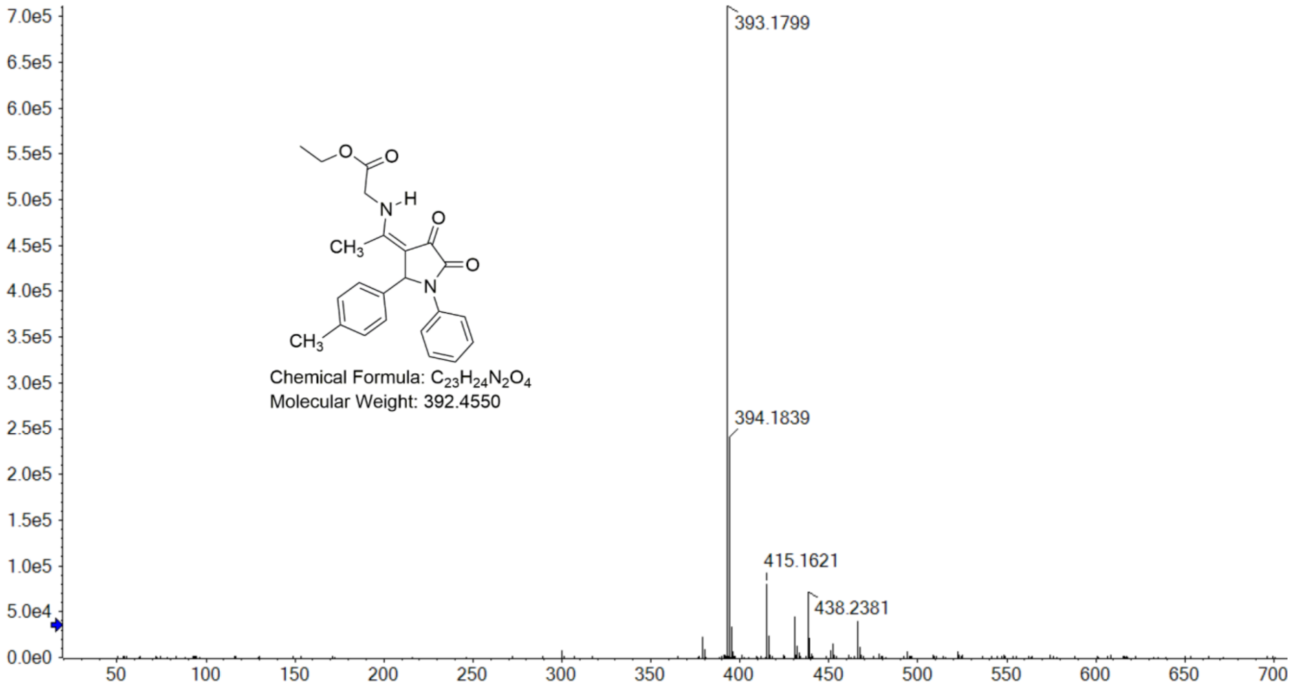

1H NMR spectrum of 4ca in CDCl3, 500MHz

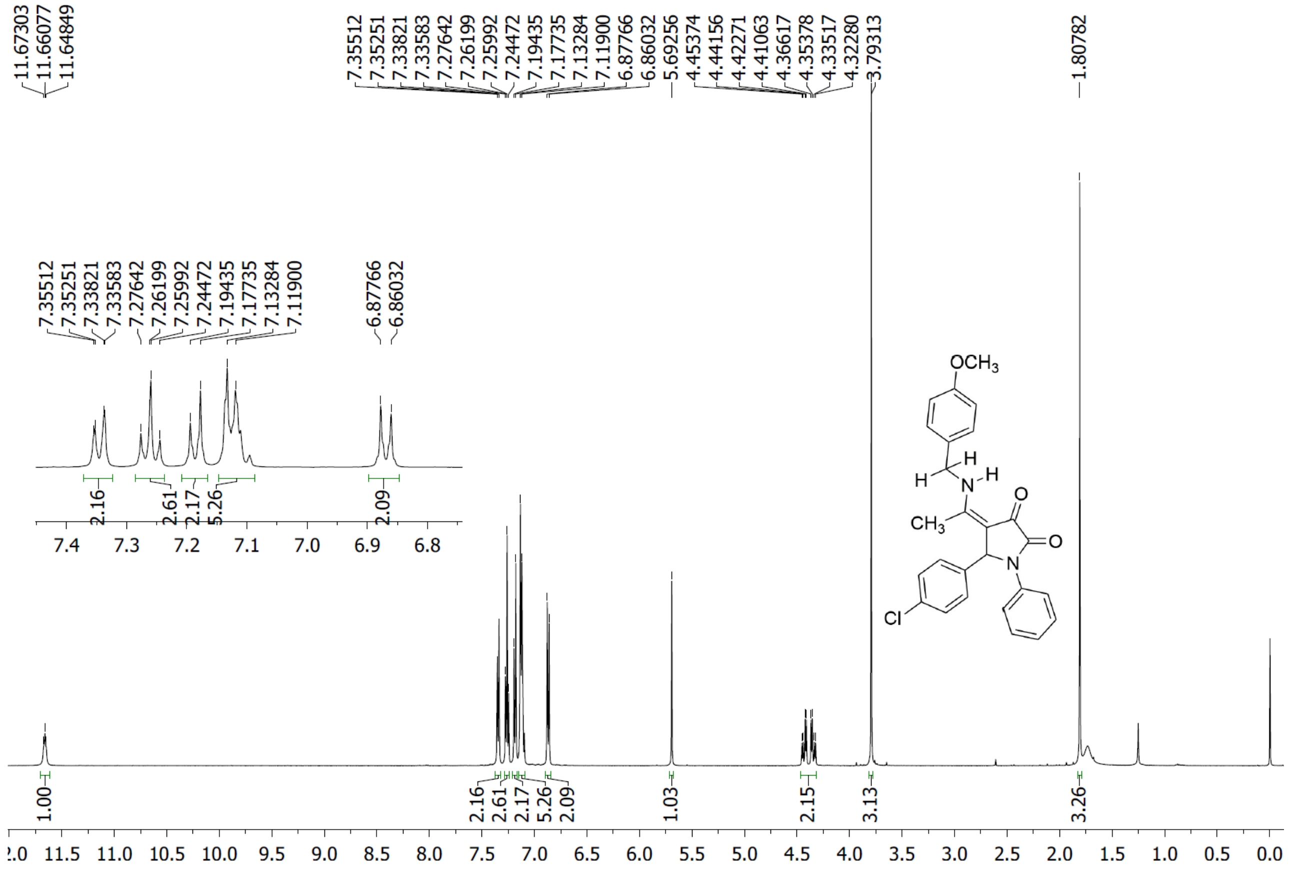

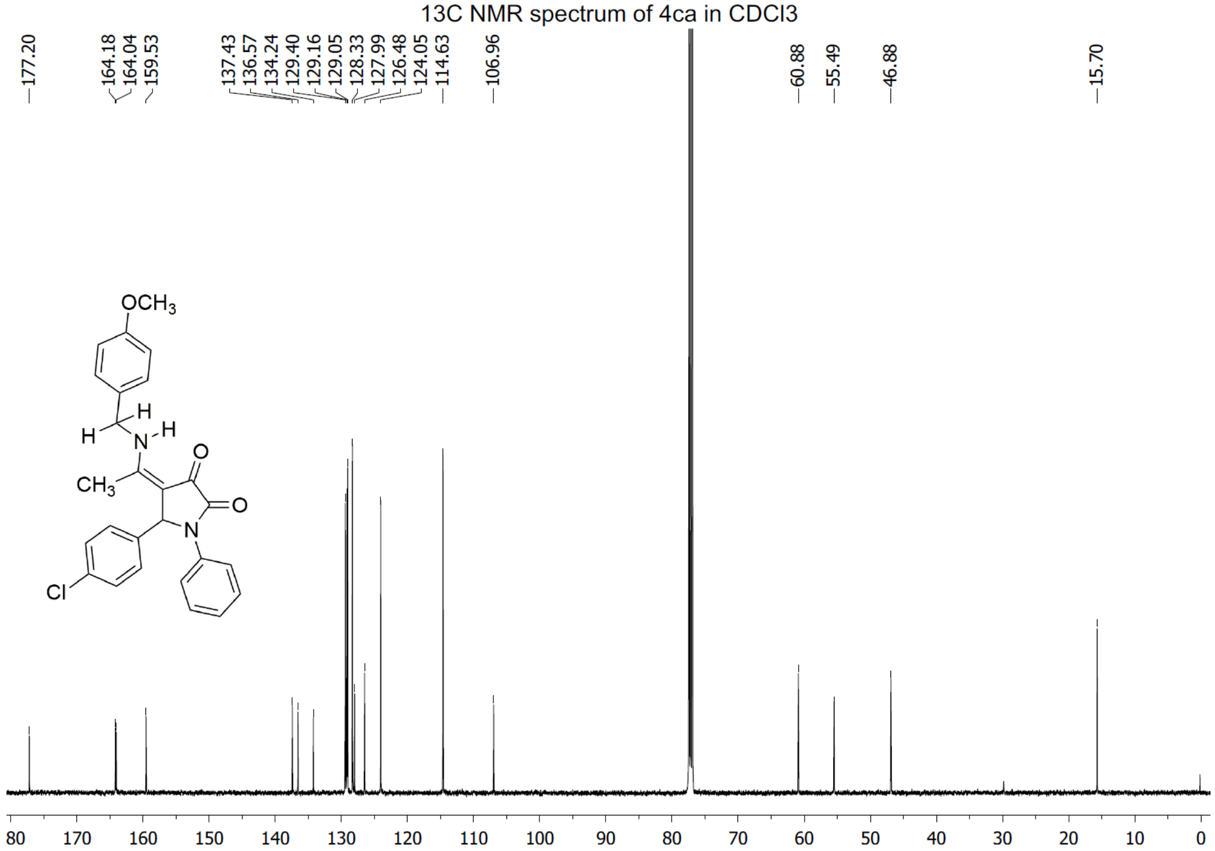
13C NMR spectrum of 4ca in CDCl3
177.20
164.18
164.04
159.53
137.43
136.57
134.24
129.40
129.16
129.05
128.33
127.99
126.48
124.05
114.63
106.96
60.88
55.49
46.88
15.70
OCH3
H
H
N
H
O
CH3
O
N
Cl
80
170
160
150
140
130
120
110
100
90
80
70
60
50
40
30
20
10
0

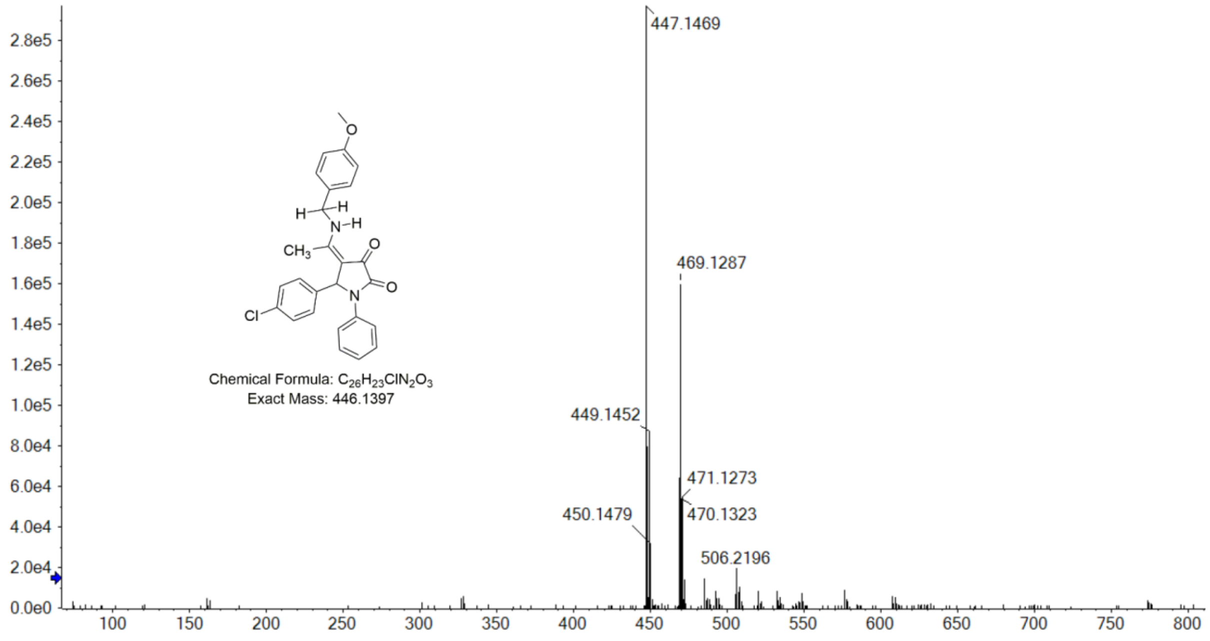
447.1469
469.1287
449.1452
471.1273
450.1479
470.1323
506.2196
Chemical Formula: C26H23ClN2O3
Exact Mass: 446.1397
2.8e5
2.6e5
2.4e5
2.2e5
2.0e5
1.8e5
1.6e5
1.4e5
1.2e5
1.0e5
8.0e4
6.0e4
4.0e4
2.0e4
0.0e0
100
150
200
250
300
350
400
450
500
550
600
650
700
750
800

1H NMR spectrum of 4cb in CDCl3, 500MHz

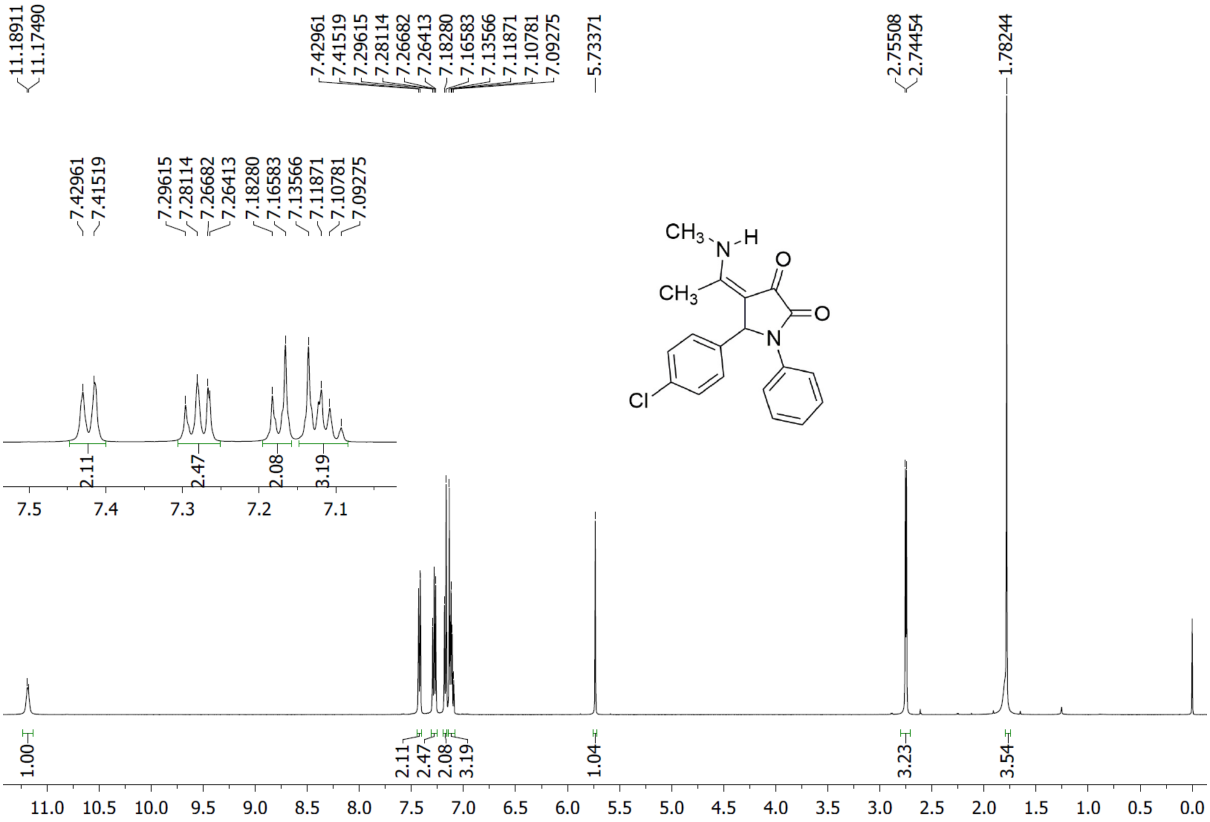

1H NMR spectrum of 4cb in CDCl3, 500MHz

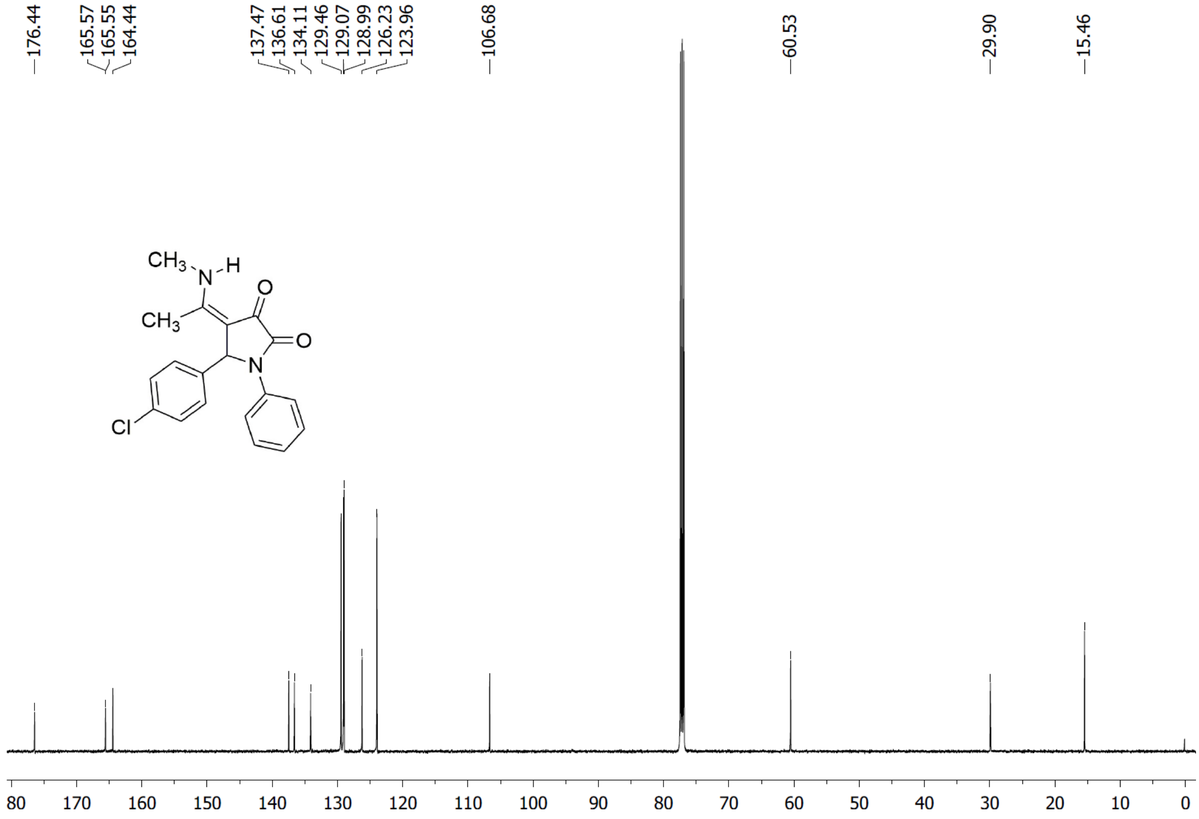

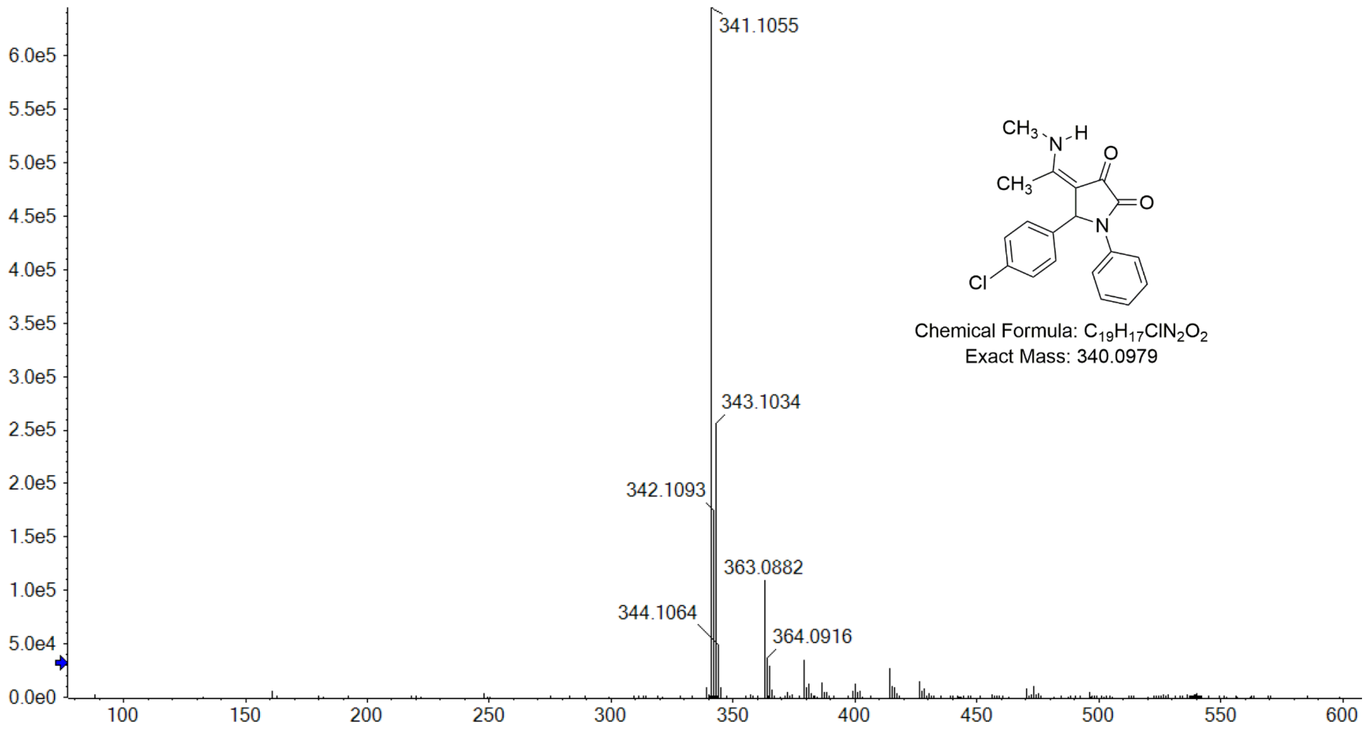

1H NMR spectrum of 4cc in CDCl3, 600MHz

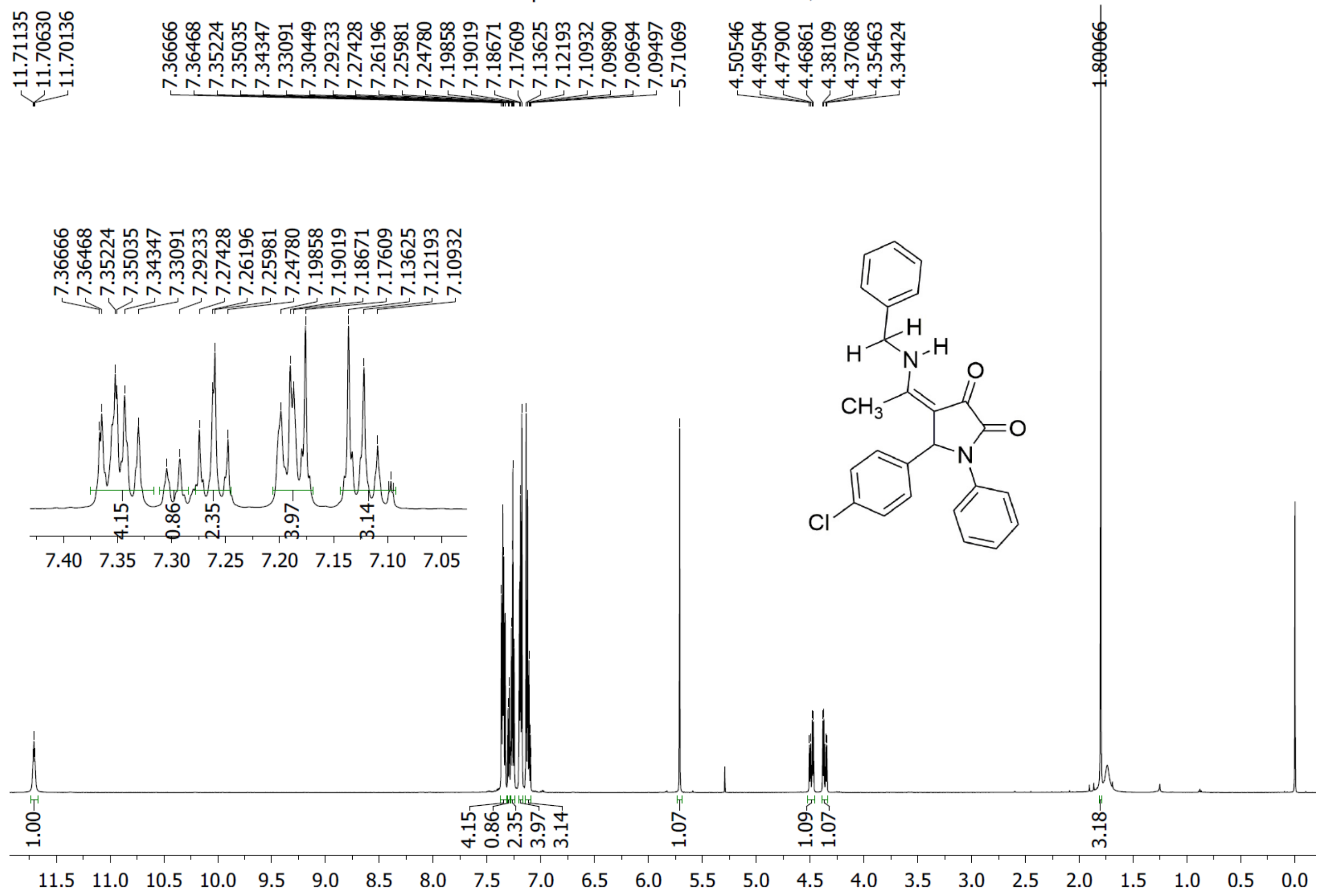

13C NMR spectrum of 4cc in CDCl3, 600 MHz

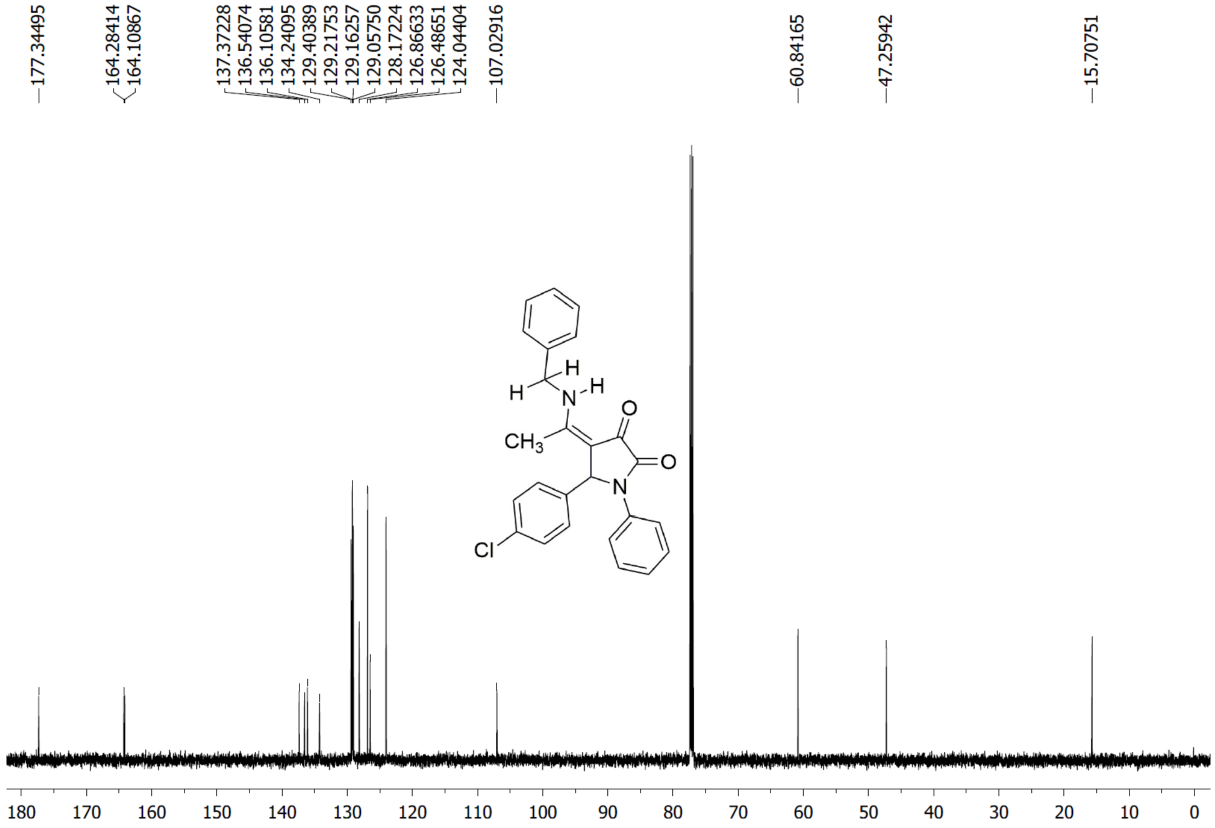

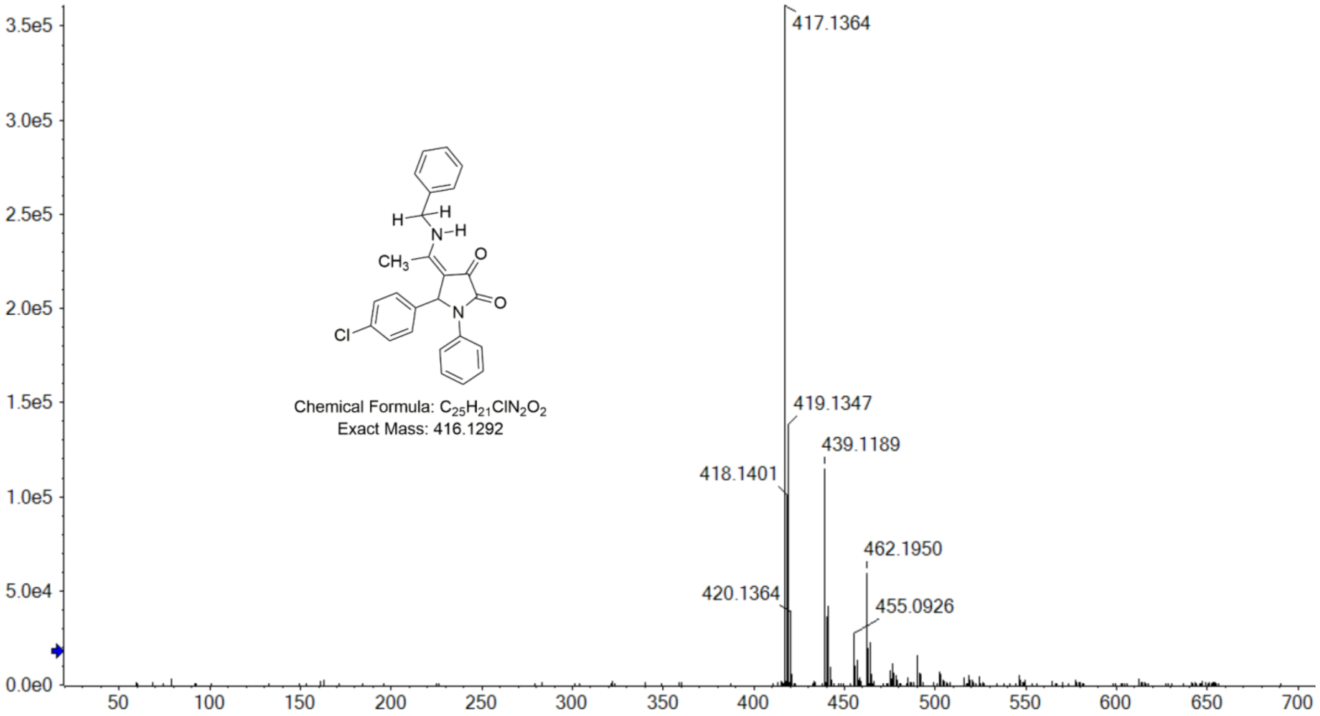

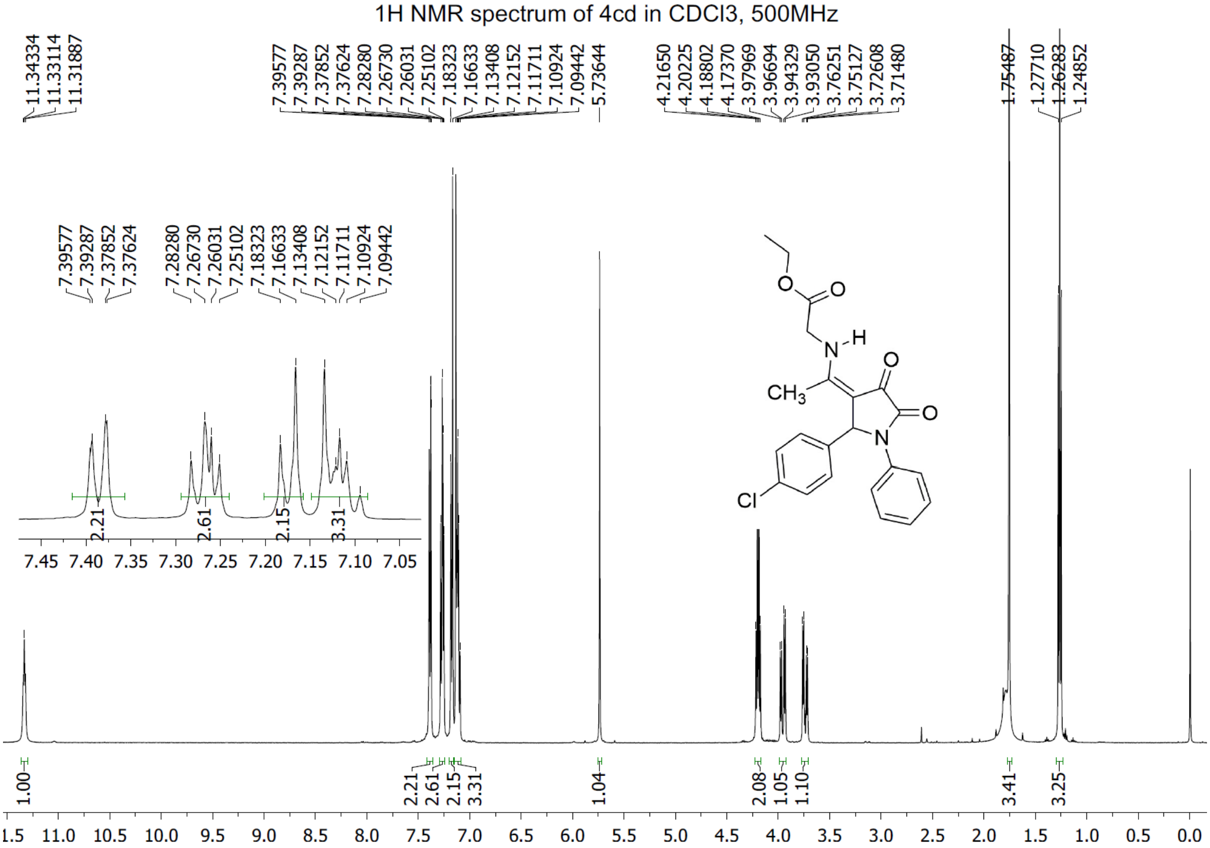
1H NMR spectrum of 4cd in CDCl3, 500MHz
11.34334
11.33114
11.31887
7.39577
7.39287
7.37852
7.37624
7.28280
7.26730
7.26031
7.25102
7.18323
7.16633
7.13408
7.12152
7.11711
7.10924
7.09442
5.73644
4.21650
4.20225
4.18802
4.17370
3.97969
3.96694
3.94329
3.93050
3.76251
3.75127
3.72608
3.71480
1.75487
1.27710
1.26283
1.24852
CH3
N
H
O
Cl
2.21
2.61
2.15
3.31
1.00
1.04
2.08
1.05
1.10
3.41
3.25

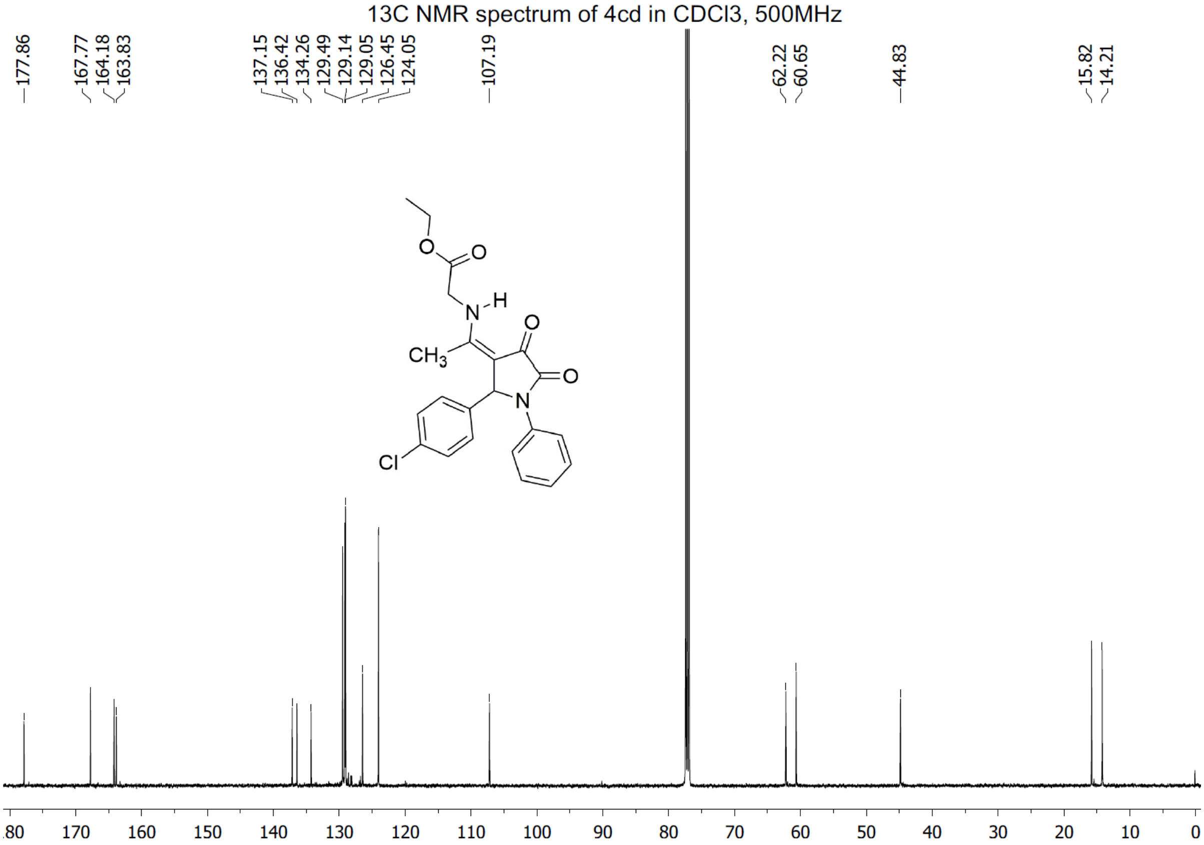
13C NMR spectrum of 4cd in CDCl3, 500MHz
177.86
167.77
164.18
163.83
137.15
136.42
134.26
129.49
129.14
129.05
126.45
124.05
107.19
62.22
60.65
44.83
15.82
14.21
CH3
N
H
O
Cl

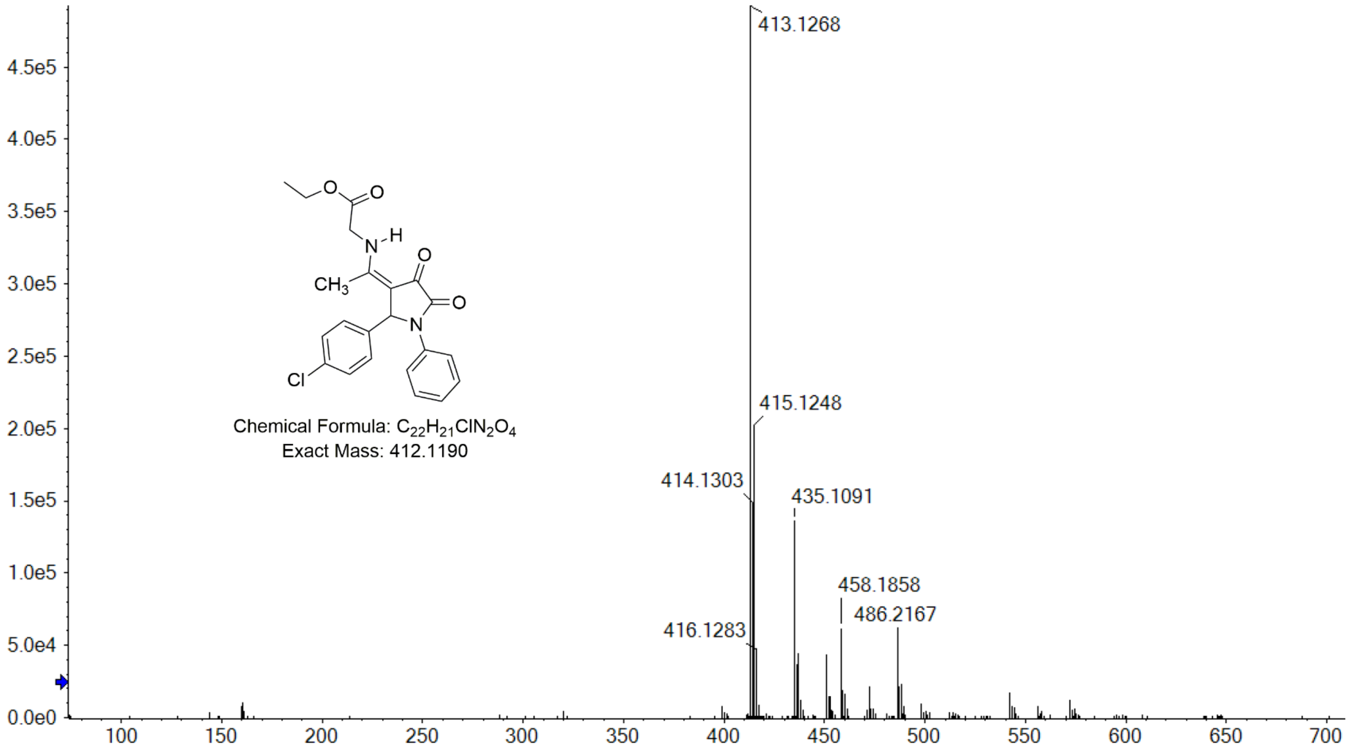
413.1268
415.1248
414.1303
435.1091
458.1858
486.2167
416.1283
Chemical Formula: C22H21ClN2O4
Exact Mass: 412.1190
4.5e5
4.0e5
3.5e5
3.0e5
2.5e5
2.0e5
1.5e5
1.0e5
5.0e4
0.0e0
100
150
200
250
300
350
400
450
500
550
600
650
700
